# Supplementary material for: Global understanding via local extraction for data clustering and visualization
Source: Patterns (N Y). 2025 May 19;6(9):101266. doi: 10.1016/j.patter.2025.101266 (PMC12485538; doi:10.1016/j.patter.2025.101266)
Supplement: Document S1. Figures S1 and S2, Tables S1–S10, and Notes S1–S5 [file mmc1.pdf]

Patterns, Volume *6*

Supplemental information

Global understanding via local extraction for data clustering and visualization

Zhenyue Zhang and Bingjie Li

Note S1. Compared Algorithms

The compared algorithms can be divided into six categories: K-means clustering, low-rank approximation, spectral method, subspace learning, agglomerative clustering, and deep networks.

K-means clustering. Lloyd's approach[1] of classic K-means on data points is widely used. It converges to a local optimum generally. The algorithm K-means++[2] uses a heuristic to choose the centroid seeds. It improves the running time and the solution of Lloyd's algorithm successfully,

Spectral methods. We compare four spectral methods: self-tuning spectral clustering (STSC)[3], sparse self-tuning spectral clustering (S-STSC)[4], landmark-based spectral clustering (LSC)[5], and self-constrained spectral clustering (SCSC)[6]. The STSC and S-STSC construct a dense self-tuned Gaussian graph and a sparse self-tuned Gaussian graph, respectively. The LSC choose p landmark points $\{u_1, \cdots, u_p\}$ with $p \ll n$ at first. Then represent each data point x_j as a p-dimensional weight vector $w_i = (w_{1i}, \cdots, w_{pj})^T$ with the normalized Gaussian weights

$$w_{ij} = \frac{g_{ij}}{\sum_k g_{kj}}, \quad g_{ij} = \begin{cases} \exp(-\frac{\|u_i - x_j\|^2}{2\sigma^2}), & i \in I_i; \\ 0, & i \notin I_i, \end{cases}$$

where I_j is the index set of r landmark points nearest to x_j and $\sigma^2 = \frac{1}{n^2}\sum_{ij}\|x_i - x_j\|^2$ is the mean of all the squared pair-wise distances of the data points. The two parameters $r \le p \ll n$ should be carefully turned for each data set. The p-dimensional points are further projected on the n_c-dimensional dominant subspace of the range space of $\{y_j\}$, and the clustering is obtained by K-means on the n_c-dimensional projected points $\{z_j\}$. Self-constrained spectral clustering (SCSC)[6] extends the traditional spectral clustering objective function with pairwise and label self-constrained terms. This approach simultaneously learns clustering results and constraints without prior information, effectively guiding the clustering process in unsupervised settings. An iterative method solves the resulting optimization problem, enabling the discovery of high-quality cluster structures in datasets.

Subspace learning. Assuming that the data points are approximately sampled from several subspaces, subspace learning aims to learn the latent subspaces by segmenting the data points into several classes corresponding to the subspaces. A self-expression approach is commonly adopted to linearly represent each point by other points that likely come from the same subspace. Hence, a similarity graph can be constructed by the representation weights. The Scalable Elastic Net Subspace Clustering (EnSC)[7] penalizes the coefficient vector in both the ℓ_1-norm and ℓ_2-norm in the self-expression, and the Efficient Dense Subspace Clustering (EDSC)[8] looks for the self-expression on a noise-free matrix of data points to decrease the interference of data noise.

Agglomerative clustering. The agglomerative clustering merges a pair of small clusters successively until all the small clusters are merged into K large ones. In[9], the initial clusters are chosen as the small k-NN neighborhoods with $k \le 2$. A direct Gaussian graph of a bit larger neighborhoods is also used to define the linkage degrees between clusters and merge the pair of current clusters having the largest linkage to a larger one. This algorithm is named as GDL (graph degree linkage), or GAL (graph average linkage) that uses the symmetric form of the graph.

Deep networks. Twenty four algorithms based on deep networks for unsupervised learning are compared: Joint Unsupervised Learning (JULE)[10], Deep Subspace Clustering Networks (DSC)[11], Deep Adversarial Subspace Clustering (DASC)[12], Discriminatively boosted clustering (DBC)[13], Deep discriminative model (DDM)[14], Graph convolutional autoencoder using Laplacian smoothing and sharpening (GALA)[15], Not Too Deep Clustering model (N2D)[16], Spectral clustering via ensemble deep autoencoder learning (EDAE)[17], Deep Subspace Image Clustering Network with Self-expression and Self-supervision (DSCNSS)[18], self-attention deep subspace clustering (SADSC)[19], deep multi-view clustering approach based on the reconstructed self-expressive matrix (DCRSM)[20], Over-complete Deep Subspace Clustering Networks (ODSC)[21], Wasserstein embedding clustering (WEC)[22], deep clustering with contractive representation learning and focal loss (DCCF)[23], Deep Fusion Clustering Network (DFCN)[24], Efficient Deep Embedded Subspace Clustering (EDESC)[25], Deep Clustering by Multi-level Feature fusion (DCMF)[26], Deep Embedded Clustering (DEC)[27], local-to-global deep clustering method based on approximate uniform manifold (LGC-AUM)[28], deep fuzzy -means clustering (RD-FKC)[29], Discriminative Pseudo Supervision Clustering (DPSC)[30], Deep Gaussian Mixture Model (DeepGMM)[31], Deep Structural Contrastive Subspace Clustering (DSCSC)[32], and deep subspace clustering via dual adversarial generative networks (DSC-DAG)[33]. As known, algorithms based on deep networks need to turn a lot of parameters involved in the network construction for each data set and cost much for computation. Generally, these deep network algorithms can achieve higher clustering accuracy than the classical methods.

Note S2. Data Information

All the tested databases reported mentioned in this paper can be downloaded online. Table S1-S2 lists the sources of these databases, together with the distance metric used to show the efficiency of the GULE in this paper. The real-world data sets come from six categories. Brief descriptions of these databases are provided below.

- Basehock[34]. This is a text dataset consisting of 1993 instances, each with 4862 features. It has 2 classes, where the maximum class size is 999, and the minimum is 994.
- COIL-20 and COIL-100[35]. These datasets were created at Columbia University. COIL-20 contains 1440 grayscale images of 20 objects, while COIL-100 has 7200 images of 100 objects. They are widely used for object recognition and pose estimation tasks. The images were taken at pose intervals of 5 degrees, resulting in 72 images per object.
- Control[36]. This is a set of time series of length 60, synthetically generated by the process described in Alcock's work . It contains 600 examples of six control charts: normal, cyclic, increasing trend, decreasing trend, upward shift, and downward shift. Each chart accounts for 100 samples.
- Fashion-MNIST[37]. This is a dataset of 28x28 grayscale images, created as a drop-in replacement for the original MNIST dataset by Zalando Research . It contains 70,000 examples of ten fashion item categories: t-shirt/top, trouser, pullover, dress, coat, sandal, shirt, sneaker, bag, and ankle boot. Each category accounts for 7,000 samples, evenly split into 6,000 training and 1,000 test images.
- HAR (Human Activity Recognition)[38]. This dataset was collected from smartphone accelerometer and gyroscope readings. It contains measurements from 30 subjects performing six activities (walking, walking upstairs, walking downstairs, sitting, standing, laying).
- Iris[39]. One of the oldest and most famous datasets in pattern recognition, the Iris dataset was introduced by Ronald Fisher in 1936. This data set has 3 classes of 50 instances each. Each class refers to a type of iris plant, and each sample is represented by a 4D attribute including sepal length, sepal width, petal length, and petal width.
- MNIST[40]. Created by Yann LeCun and colleagues, MNIST (Modified National Institute of Standards and Technology database) consists of 70,000 handwritten digit images, split into 60,000 training images and 10,000 test images. In this experiment, we use both the whole set (MNIST70K), the testing set(MNIST), and the preprocessed data set (MNIST-PT).
- Olive Face (ORL)[41].This is a set of facial images from the Olivetti faces database, originally compiled by AT&T Laboratories Cambridge. It comprises 400 grayscale images of 40 distinct subjects, with each subject represented by 10 samples. These samples capture various facial expressions, lighting conditions, and facial details (e.g., with/without glasses). In our experiment, we utilize two versions of this dataset: Olive and ORL, which differ only in resolution. The Olive version consists of 64x64 pixel images, while the ORL version maintains the original 92x112 pixel resolution. Both variants provide a standardized format for facial recognition tasks and algorithm development, allowing for comparative analysis across different image resolutions.
- Pendigit[42]. This is a set of 10992 16-dimensional vectors. Each is a pen-based trace of 8 temporally sampled (x, y)-coordinates of a handwritten digit from one of 44 writers. As a set of handwritten digits, it also has 10 classes.
- Pcmac[34]. This is a text classification dataset with 1943 instances and 3289 features divided into 2 classes, derived from documents related to PCs and Macs.
- PIE[43]. This data set has 2856 face images of 68 persons in the small size 32×32. Each person has 42 facial images under different lighting and illumination conditions.
- Relathe[34]. Another text classification dataset with 1427 instances and 4322 features divided into 2 classes. It is used for evaluating text classification algorithms.
- Satellite[44]. This is a set of 6435 multi-spectral points of seven soil cover states: red soil, cotton crop, grey soil, damp grey soil, soil with vegetation stubble, mixture class, and very damp grey soil. Each point is a 36-dimensional vector, consisting of 4 spectral values of each pixel in a 3×3 region of a satellite image.
- Seeds[45]. This is a data set of 210 wheat seeds divided into 3 classes. Each sample contains 7 attributes: area, perimeter, compactness, kernel length, kernel width, asymmetry coefficient, and length of kernel groove.

- Sports[46]. This is a text dataset of sports news articles, collected from various online sources. It contains 1,000 examples of two sports categories. The articles are in English and vary in length, with an average of approximately 400 words per article.
- UMist[47]. This data set contains 565 images of 20 persons. Each person covers a range of poses from profile to frontal views and has various numbers of images.
- USPS[48]. This is a set of normalized gray-scale images of handwritten digits in size 16×16, automatically scanned from envelopes by the U.S. Postal Service. It contains 7291 training examples and 2007 test examples. We use the whole set, with a total of 9298 images.

Note S3. Mathematical Analysis of Graph Perturbations and Theorems

Assume that we are given a neighbor set N_i for each vertex v_i, and the neighbors of v_i are likely class-consistent with the centroid v_i. Under this assumption, the block partition of A,

$$A = \begin{bmatrix} A_{11} & \cdots & A_{1K} \\ \vdots & \ddots & \vdots \\ A_{K1} & \cdots & A_{KK} \end{bmatrix}$$

has sparse and relatively small off-diagonal blocks $A_{k\ell}$ with $\ell \neq k$. That is, A approximates the block-diagonal matrix

$$A_0 = \mathrm{diag}(A_{11}, \dots, A_{KK}).$$

The diagonal blocks $\{A_{kk}\}$ may be not connected, that is, each A_{kk} can be further partitioned as $A_{kk} = \mathrm{diag}\big(A_1^{(k)}, \dots, A_{p_k}^{(k)}\big)$ without loss of generalities, where each $A_i^{(k)}$ is connected.

If the neighborhoods are small, the diagonal blocks $\{A_t^{(k)}\}$ of A, are sparse and the connection within blocks may be very weak. Diagonal rescaling such as $D^{-1/2}AD^{-1/2}$ in the normalized cutting can strengthen the small diagonal blocks in the spectral projection, where D is the diagonal matrix of the column vector

$$r(A) = (r_1(A), \dots, r_n(A))^T \quad \text{with} \quad r_i(A) = a_{i1} + \cdots + a_{in}.$$

However, if the neighborhoods are relatively large, each A_{kk} has a dominant block, say $A_1^{(k)}$, that covers most of class-consistent points. The diagonal rescaling may overly strengthen very small blocks and depress the dominant block. Based on the rescaled graph, the spectral method may result in a wrong projection.

To match the neighborhood size suitably, we suggest an adaptive scaling to the adjacency matrix A as

$$G = D^{-\beta/2}AD^{-\beta/2},$$

where the parameter $\beta \in [0, 1]$ tunes the diagonal scaling to match the neighborhood size: the larger the neighborhood size is, the smaller β is. Basically, β tunes the distributions of the K eigenvectors of the rescaled graph G as show below.

The property of the spectral projection on G will be exploited, utilizing a useful structure behind the block-partition of $G = (G_{k\ell})$ under the partition of $A = (A_{k\ell})$ and $A_k = [A_{k1}, \dots, A_{kK}]$, where

$$G_{k\ell} = D_k^{-\beta/2} A_{k\ell} D_\ell^{-\beta/2}, \quad D_k = \mathrm{diag}(r(A_k)),$$

and $r(A_k) = (r_1(A_k), \dots, r_{n_k}(A_k))^T$. The key ideal is to take G as an approximate of the block diagonal matrix

$$G_0 = \mathrm{diag}(\bar{G}_{11}, \dots, \bar{G}_{KK}), \text{ where } \bar{G}_{kk} = \bar{D}_k^{-\beta/2} A_{kk} \bar{D}_k^{-\beta/2}, \quad \bar{D}_k = \mathrm{diag}(r(A_{kk})),$$

due to the two observations: Good approximation of G to G_0, and spatial structures of the eigenvectors U_0 of G_0 corresponding to the K largest eigenvector. These two properties help us to exploit the class information from the K largest eigenvectors (the eigenvectors corresponding to the largest eigenvalues) of the graph G, via a special clustering approach that will be given in the later section. Below, we will show these propositions.

Approximation

The following term will be used in the error estimation.

$$A_k^c = [A_{k1}, \dots, A_{k,k-1},\ 0,\ A_{k,k+1}, \dots, A_{kK}],$$

$$\rho = (\rho_1; \dots; \rho_K), \quad \rho_k = \Big(\frac{r_1(A_k^c)}{r_1(A_{kk})}; \dots; \frac{r_{n_k}(A_k^c)}{r_{n_k}(A_{kk})}\Big),$$

$$\rho^{(\beta)} = \big(\rho_1^{(\beta)}; \dots; \rho_K^{(\beta)}\big), \quad \rho_k^{(\beta)} = \Big(\frac{r_1(A_k^c)}{r_1^\beta(A_{kk})}; \dots; \frac{r_{n_k}(A_k^c)}{r_{n_k}^\beta(A_{kk})}\Big).$$

where the semicolon means a column link. Clearly, $\rho_k^{(\beta)} = \rho_k$ when $\beta = 1$; or $\rho_k^{(\beta)} = (r_1(A_k^c); \ldots; r_{n_k}(A_k^c))$ if $\beta = 0$. Generally, $\rho_k^{(\beta)}$ and ρ_k are small if the class-consistent neighborhoods are estimated well.

Lemma 1. *The error matrix* $E = G - G_0$ *is bounded as* $\|E\|_2 \le (1+\beta)\|\rho^{(\beta)}\|_\infty$.

Proof. Representing the vector $r(A_k)$ as $r(A_k) = r(A_{kk}) + r(A_{kk}^c) = r(A_{kk}) \odot (1 + \rho_k)$,

$$D_k = \bar{D}_k J_k, \quad J_k = I + \mathrm{diag}(\rho_k).$$

and $G_{kk} = J_k^{-\beta/2}\bar{G}_{kk}J_k^{-\beta/2}$. Write $J_k^{-\beta/2} = I - \Delta_k$ with $\Delta_k = I - J_k^{-\beta/2}$ for simplicity. Then,

$$G_{kk} - \bar{G}_{kk} = (I - \Delta_k)\bar{G}_{kk}J_k^{-\beta/2} - \bar{G}_{kk} = \bar{G}_{kk}(J_k^{-\beta/2} - I) - \Delta_k\bar{G}_{kk}J_k^{-\beta/2} = -\bar{G}_{kk}\Delta_k - \Delta_k\bar{G}_{kk}J_k^{-\beta/2}.$$

Hence, by $\|J_k^{-\beta/2}\|_2 \le 1$ and using $\lambda_1(AB) = \lambda_1(BA)$ and $\lambda_1(C) \le \|r(C)\|_\infty$, we see that

$$\begin{aligned}\|G_{kk} - \bar{G}_{kk}\|_2 &\le 2\|\Delta_k\bar{G}_{kk}\|_2 = 2\|\Delta_k\bar{D}^{-\beta/2}A_{kk}\bar{D}^{-\beta/2}\|_2 \\ &= 2\lambda_1\big(\Delta_k\bar{D}^{-\beta/2}A_{kk}\bar{D}^{-\beta/2}\big) = 2\lambda_1\big(\Delta_k\bar{D}^{-\beta}A_{kk}\big) \le 2\|r(\Delta_k\bar{D}^{-\beta}A_{kk})\|_\infty \\ &= 2\|\big(I - (I + \mathrm{diag}(\rho_k))^{-\beta/2}\big)\mathrm{diag}(r(A_{kk}))^{-\beta}r(A_{kk})\|_\infty.\end{aligned}$$

By $0 \le 1 - (1+t)^{-\beta/2} \le \frac{\beta}{2}t$, it follows that

$$\|G_{kk} - \bar{G}_{kk}\|_2 \le \beta\max_i(\rho_k)_i(r_i(A_{kk}))^{1-\beta} = \beta\max_i(\rho_k^{(\beta)})_i = \beta\|\rho_k^{(\beta)}\|_\infty.$$

Hence, the block diagonal part E_0 of E is bounded as

$$\|E_0\|_2 = \max_k\|G_{kk} - \bar{G}_{kk}\|_2 \le \beta\|\rho^{(\beta)}\|_\infty$$

We also have the estimation on the off-block diagonal part E_0^c:

$$\|E_0^c\|_2 = \|D^{-\beta/2}[A_1^c; \cdots; A_K^c]D^{-\beta/2}\|_2 = \lambda_1(D^{-\beta}[A_1^c; \cdots; A_K^c]) \le \max_{k,i}\frac{r_i(A_k^c)}{r_i^\beta(A_k)} = \|\rho^{(\beta)}\|_\infty$$

Therefore, $\|E\|_2 \le \|E_0\|_2 + \|E_0^c\|_2 \le (1+\beta)\|\rho^{(\beta)}\|_\infty$. □

Structure of the eigenvectors of G

The block-diagonal G_0 has K sparse and nonnegative eigenvectors. Practically, since $\bar{G}_{kk}$ is nonnegative, by Perron-Frobenius theorem[49], it has a nonnegative eigenvector $\bar{u}_k$ corresponding to its largest eigenvalue $\lambda_1(\bar{G}_{kk})$. The eigenvector can be positive when $\bar{G}_{kk}$ is connected. Hence, let $u_k^{(0)}$ be the the sparse vector taking $\bar{u}_k$ as its nonzero piece with the index set as that of A_{kk} in A. Then $\{(\lambda_1(\bar{G}_{kk}), u_k^{(0)})\}$ are K eigen-pairs of G. Hence, $U_0 = [u_1^{(0)}, \ldots, u_K^{(0)}]$ has K row-blocks $\{u_k^{(0)}e_k^T\}$, where e_k is the k-th column of the identity matrix of order K.

For the K eigenvectors U of G corresponding to the K largest eigenvalues, the following lemma shows that there is an orthogonal matrix Q such that UQ has the structure as U_0 approximately.

Lemma 2. *Let* $U = [u_1, \ldots, u_K]$ *be the* K *eigenvectors of* G *corresponding to the* K *largest eigenvalues. Then there is an orthogonal matrix* Q *such that*

$$\|U_0 - UQ\|_F \le \frac{2\sqrt{2}\min\{\sqrt{K}\|E\|_2, \|E\|_F\}}{\lambda_K(G_0) - \lambda_{K+1}(G_0)}. \tag{1}$$

Generally, if $\min_k\lambda_1(\bar{G}_{kk}) > \max_k\lambda_2(\bar{G}_{kk})$, then

$$\lambda_K(G_0) - \lambda_{K+1}(G_0) = \min_k\lambda_1(\bar{G}_{kk}) - \max_k\lambda_2(\bar{G}_{kk}).$$

We omit the proof since it is an application of the perturbation theorem of invariant subspace given in[50] for symmetric matrices.

Clustering and accuracy

If each A_{kk} is connected, all the entries of $\bar{u}_k$ are positive. Hence, the ground-truth classes $\{C_k^*\}$ can be exactly separated according to the labeling

$$\ell_i^* = \arg\max_k u_{ik}^{(0)}.$$

That is, $C_k^* = \{i : \ \ell_i^* = k\}$. Thus, as soon as Q is available, the true classes can also be well estimated as $C_k = \{i : \ \ell_i = k\}$ according to the clustering rule

$$\ell_i = \arg\max_k |(UQ)_{ik}|, \quad i = 1, \cdots, n. \tag{2}$$

The following theorem gives an estimation to the accuracy according to above clustering rule.

Theorem 1. *Let $\varepsilon = \beta\|\rho\|_F + \tau^{\beta/2}\|\rho^{(\beta)}\|_F$, $\{\eta_p^2\}$ are the ordered squared entries of UQ in ascending order, and*

$$p = \arg\max\Big\{p : \ \eta_1^2 + \cdots + \eta_p^2 \le \frac{8\varepsilon^2}{\delta_K^2(G_0)}\Big\}. \tag{3}$$

If each $\bar{G}_{kk}$ is connected and $\delta(G_0) > 0$, then there are at most p points miss-clustered according to the clustering rule (2),

Proof. Let $UQ = (\tilde{u}_{ik})$ and $\mathcal{I}_k = \big\{i \in C_k^* : \ |\tilde{u}_{ik}| \le \max_{\ell\neq k}|\tilde{u}_{i\ell}|\big\}$. Only those vertices with indices in $\mathcal{I} = \cup_k \mathcal{I}_k$ may be miss-labeld by (2). By definition,

$$\sum_k \sum_{i\in\mathcal{J}_k} \tilde{u}_{ik}^2 \le \|U_0 - UQ\|_F^2 \le \frac{8\varepsilon^2}{\delta_K^2(G_0)}.$$

Since only the smallest p squared entries have a sum not lager than $\frac{8\varepsilon^2}{\delta_K^2(G_0)}$, we conclude that $|\mathcal{J}| = \sum_k |\mathcal{I}_k| \le p$, completing the proof. ☐

Note S4. Evaluation metrics

Clustering Evaluation metrics

We used three commonly used clustering accuracy metrics, including Accuracy (ACC) , Adjusted Rand Index (ARI)[51], and Normalized Mutual Information (NMI)[52]. These metrics assess the similarity between the clustering results and the real label of each dataset.

Given a set S of n elements, and two clusters or partitions of these elements, namely $X = \{X_1, \cdots, X_r\}$ and $Y = \{Y_1, \cdots, Y_s\}$ with $r \ge s$. We defined $n_{ij} = \text{card}\{X_i \bigcap Y_j\}$, $n_{i\cdot} = \sum_{j=1}^s n_{ij} = \text{card}\{X_i\}$, and $n_{\cdot j} = \sum_{i=1}^r n_{ij} = \text{card}\{Y_j\}$, where card$\{\cdot\}$ denotes the size of a set. The ACC measures the proportion of correctly assigned data points and is calculated as the average of the maximum number of data points assigned to the same cluster:

$$\text{ACC} = \frac{1}{n}\sum_{i=1}^r \max_j(n_{ij}). \tag{4}$$

The calculation of the Adjusted Rand Index (ARI) involves the use of the combination number $\binom{n}{2}$, which represents the total number of possible pairs that can be formed from a set of n data points. Specifically, the ARI is calculated as:

$$\text{ARI} = \frac{\sum_{i=1}^r \sum_{j=1}^s \binom{n_{ij}}{2} - \left[\sum_{i=1}^r \binom{n_{i\cdot}}{2} \sum_{j=1}^s \binom{n_{\cdot j}}{2}\right] / \binom{n}{2}}{\frac{1}{2}\left[\sum_{i=1}^r \binom{n_{i\cdot}}{2} + \sum_{j=1}^s \binom{n_{\cdot j}}{2}\right] - \left[\sum_{i=1}^r \binom{n_{i\cdot}}{2} \sum_{j=1}^s \binom{n_{\cdot j}}{2}\right] / \binom{n}{2}}. \tag{5}$$

Finally, the definition of NMI is the normalization of Mutual Information(MI). NMI takes into account the entropy between partitions to improve the accuracy of the comparison as

$$H(X) = -\frac{1}{n}\sum_{i=1}^r n_{i\cdot}\log\left(\frac{n_{i\cdot}}{n}\right) \quad H(Y) = -\frac{1}{n}\sum_{j=1}^s n_{\cdot j}\log\left(\frac{n_{\cdot j}}{n}\right), \tag{6}$$

and the definition of NMI is

$$\text{NMI} = \frac{2\sum_{i=1}^r \sum_{j=1}^s n_{ij}\left[\log(nn_{ij}) - \log(n_{i\cdot}n_{\cdot j})\right]}{n(H(X) + H(Y))}. \tag{7}$$

Visualization Evaluation metrics

To assess the quality of dimensionality reduction and visualization techniques, we employed the Silhouette Coefficient[53]. While primarily used for clustering evaluation, SC can also assess the quality of dimensionality reduction.

The Silhouette Coefficient for a single sample is given as:

$$s(i) = \frac{b(i) - a(i)}{\max\{a(i), b(i)\}} \tag{8}$$

where $a(i)$ is the mean distance between a sample and all other points in the same class (intra-class distance), and $b(i)$ is the mean distance between a sample and all other points in the next nearest cluster (nearest-neighbor inter-class distance).

The Silhouette Coefficient for a set of samples is given as the mean of the Silhouette Coefficient for each sample:

$$SC = \frac{1}{n} \sum_{i=1}^{n} s(i) \tag{9}$$

where n is the total number of samples.

The Silhouette Coefficient ranges from -1 to 1, where a higher value indicates that the object is well-matched to its own cluster and poorly-matched to neighboring clusters. This metric provides insight into both the cohesion within clusters and the separation between clusters in the low-dimensional representation.

Graph quality metrics

Graph score is a metric for evaluating the quality of weighted graph structures, combining precision and recall to measure the consistency between graph connections, their weights, and node categories. For a weighted graph $G(V, E, A)$, where V is the set of nodes, E is the set of edges, A is the set of edge weights, and C is the set of node categories, the graph score is defined as:

$$\text{Score}(G) = \text{Precision}(G) \odot \text{Recall}(G)$$

where $\odot$ represents element-wise multiplication (Hadamard product),

$$\text{Precision}(G)_i = \frac{\left[\sum_j A_{ij} \cdot \mathbb{I}(C(i) = C(j))\right]}{\left[\sum_j A_{ij}\right]}$$

$$\text{Recall}(G)_i = \frac{\left[\sum_j A_{ij} \cdot \mathbb{I}(C(i) = C(j))\right]}{\left[\sum_j \mathbb{I}(C(i) = C(j))\right]}$$

Here, A_{ij} is the element in the adjacency matrix representing the weight of edge (i, j), $\mathbb{I}(\cdot)$ is the indicator function, $C(i)$ represents the category of node i, and $[\ ]/[\ ]$ denotes element-wise division. Score(G) is a vector where each element corresponds to the score of a node.

Graph score is a metric for detecting the quality of weighted graphs, assessing the consistency between the graph structure and node category labels. A high score indicates that the graph's connection patterns highly align with the distribution of node categories: nodes of the same category tend to be closely connected by high-weight edges, while connections between different categories are weaker or absent. This metric combines precision (category consistency of existing connections) and recall (completeness of intra-category connections), providing a comprehensive measure of the graph's overall quality. It is particularly suitable for evaluating graphs with evident community structures or for validating the effectiveness of graph construction algorithms.

Note S5. RNA-seq data sets

GTEx Brain data set

Below is a description of the eight anatomical classes, with each paragraph discussing the role of the regions included in that class. The original data were labeled with 13 anatomical regions, but these regions were

reclassified into 8 broader categories. Note that the cells in the original 13 anatomical regions may originate from different donors. Some regions, such as parts of the cortex, overlap with areas grouped under other categories. Similarly, there is overlap between the Cerebellar Hemisphere and the Cerebellum. Due to these overlaps, making a strict separation between such regions is often not possible and may not be meaningful in the context of single-cell analysis.

- **Cortex:** This group includes regions such as the Anterior Cingulate Cortex and the Frontal Cortex. These cortical areas are fundamental for high-level cognitive processes including decision making, emotional regulation, attention, and conflict monitoring. The anterior cingulate cortex is especially known for its role in error detection and regulating emotional responses, while the frontal cortex is pivotal for planning, executive control, and social behavior[54].
- **Basal Ganglia:** In this group, key regions include the Putamen (basal ganglia) along with other dorsal striatal structures such as the Caudate. The basal ganglia are central to the initiation and control of voluntary movements, habit formation, and aspects of reward processing. Although these regions share similar cell types (mainly GABAergic medium spiny neurons), the Putamen is particularly involved in motor planning and execution. It differs from regions like the nucleus accumbens (not included in this group) by its primary role in motor function rather than in reward and motivational processing[55,56].
- **Cerebellum:** This class comprises both the Cerebellar Hemisphere and the Cerebellum proper. The cerebellum is traditionally recognized for coordinating movement, balance, and motor learning, and it also contributes to cognitive processes such as timing and error correction. The cerebellar hemisphere is mainly associated with lateralized motor and cognitive functions, while the midline structures play a key role in regulating axial and postural control[57].
- **Amygdala:** The Amygdala is a core limbic structure essential for processing emotional stimuli, particularly fear and anxiety, and for forming emotional memories. It interacts closely with cortical and hippocampal networks to assign affective significance to sensory inputs and to guide behavior based on emotional cues[58].
- **Hippocampus:** The Hippocampus is vital for the formation and consolidation of new memories and plays an essential role in spatial navigation. Its complex circuitry—including subfields such as CA1, CA3, and the dentate gyrus—underpins its capacity for neuroplasticity, which is fundamental to learning and memory processes[59].
- **Hypothalamus:** The Hypothalamus regulates a variety of homeostatic processes including hunger, thirst, body temperature, and circadian rhythms. It is also critical for controlling the sleep-wake cycle through its coordination of autonomic and endocrine responses. Various hypothalamic nuclei work together to maintain the body's internal balance and modulate responses to stress and environmental changes[60].
- **Spinal Cord:** Although not part of the brain, the Spinal Cord is an essential component of the central nervous system. It acts as the primary pathway for transmitting motor commands from the brain to the body and relaying sensory information upward. It plays a key role in reflex actions and in the execution of coordinated movement patterns.
- **Substantia Nigra:** The Substantia Nigra, located in the midbrain, is a major source of dopamine for the basal ganglia circuitry. Its dopaminergic neurons are critical for modulating motor control, influencing both movement initiation and execution. Dysfunction in this region is closely associated with movement disorders such as Parkinson's disease[61].

The Zeisel Dataset

The Zeisel dataset, as reported in[62], contains transcriptomic profiles for 3005 single cells isolated from the mouse cortex and hippocampus. In this study, the cells were classified into seven distinct cell types based on their gene expression profiles. Below is a description of each of these seven classes and the roles they play:

- **astrocytes_ependymal:** This class includes both astrocytes and ependymal cells. Astrocytes provide metabolic support, modulate synaptic activity, and help maintain the extracellular environment, while ependymal cells are involved in the production and circulation of cerebrospinal fluid.
- **endothelial-mural:** This group is composed of endothelial cells along with associated mural cells (such as pericytes and smooth muscle cells). These cells are essential for forming and maintaining the blood-brain barrier and regulating cerebral blood flow.

- **interneurons:** Interneurons are primarily inhibitory neurons that regulate local circuit activity. They play a critical role in balancing excitation and inhibition within neural networks, thereby ensuring proper signal modulation.
- **microglia:** Microglia are the resident immune cells of the central nervous system. They act as the primary mediators of immune responses in the brain by clearing debris, pathogens, and damaged cells, and by modulating inflammatory processes.
- **oligodendrocytes:** Oligodendrocytes are responsible for myelinating axons in the central nervous system. Myelination increases the speed and efficiency of electrical signal conduction along neuronal processes.
- **pyramidal CA1:** This class represents the excitatory pyramidal neurons found in the CA1 region of the hippocampus. These neurons are critical for memory formation and spatial navigation, as they integrate and transmit information within hippocampal circuits.
- **pyramidal SS:** Pyramidal neurons in this group are located in the somatosensory (SS) cortex. They play an important role in processing sensory information and are key components of cortical excitatory circuits.

The Darmanis Dataset

The Darmanis dataset, as described in Darmanis et al. (2015)[63], comprises transcriptomic profiles for 466 single cells isolated from the human brain. In this study, the cells were classified into nine distinct cell types. Below is a description of each cell type and their general functional roles:

- **OPC:** Oligodendrocyte Progenitor Cells (OPCs) are precursor cells that give rise to oligodendrocytes. They play a critical role in the development and maintenance of myelination in the brain.
- **astrocytes:** Astrocytes are glial cells that provide metabolic and structural support to neurons, regulate extracellular ion balance, and modulate synaptic transmission and plasticity.
- **endothelial:** Endothelial cells form the lining of blood vessels and are crucial for maintaining the blood-brain barrier, thus controlling the passage of substances between the bloodstream and the brain.
- **fetal_quiescent:** This category includes fetal brain cells that are in a quiescent (non-dividing) state. These cells are thought to represent a more mature, differentiated state within the developing brain.
- **fetal_replicating:** In contrast, the fetal_replicating cells are actively dividing. They represent a proliferative population in the fetal brain, contributing to brain development.
- **hybrid:** The hybrid class comprises cells that exhibit mixed or intermediate gene expression profiles. These cells may represent transitional states between defined cell types.
- **microglia:** Microglia are the resident immune cells of the brain. They monitor the environment for signs of infection or injury and play key roles in inflammation and tissue repair.
- **neurons:** Neurons are the primary excitatory cells in the brain responsible for transmitting electrical signals, processing information, and forming complex neural networks.
- **oligodendrocytes:** Oligodendrocytes are specialized glial cells responsible for producing myelin, the insulating sheath that increases the speed and efficiency of electrical signal transmission along axons.

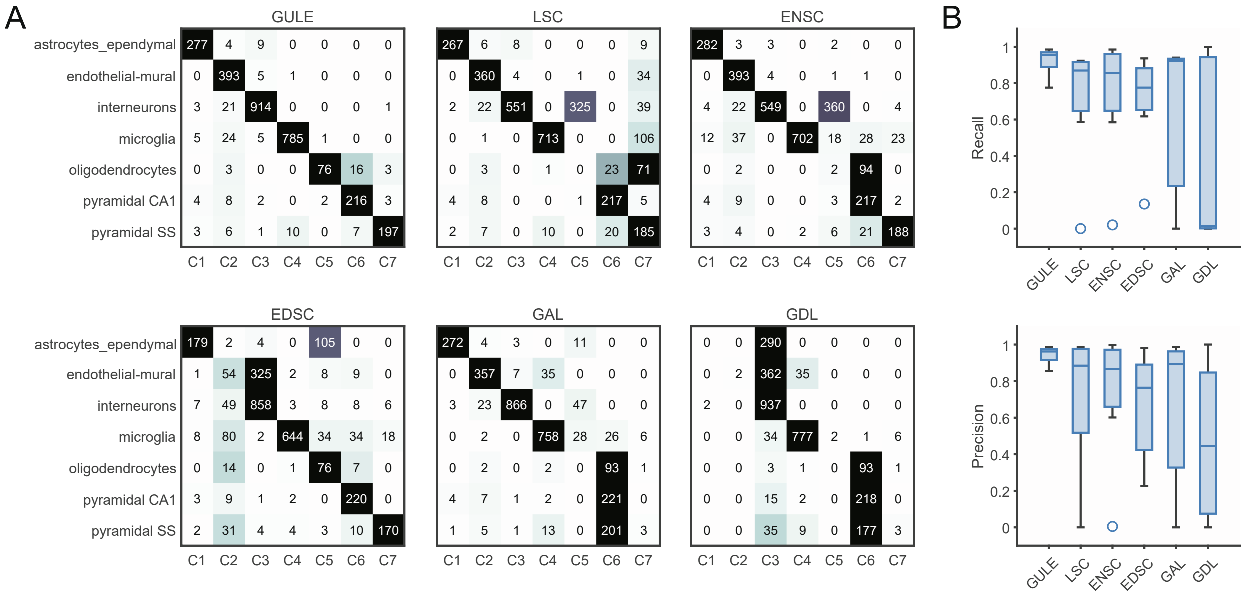

Figure S1: **Performance comparison of brain cell clustering methods on the Zeisel mouse brain dataset.** (A). Confusion matrices of different clustering methods on the Zeisel mouse brain dataset containing 3005 cells from seven major cell types. The rows represent true cell types and columns represent predicted clusters. (B) Recall and precision of each method.

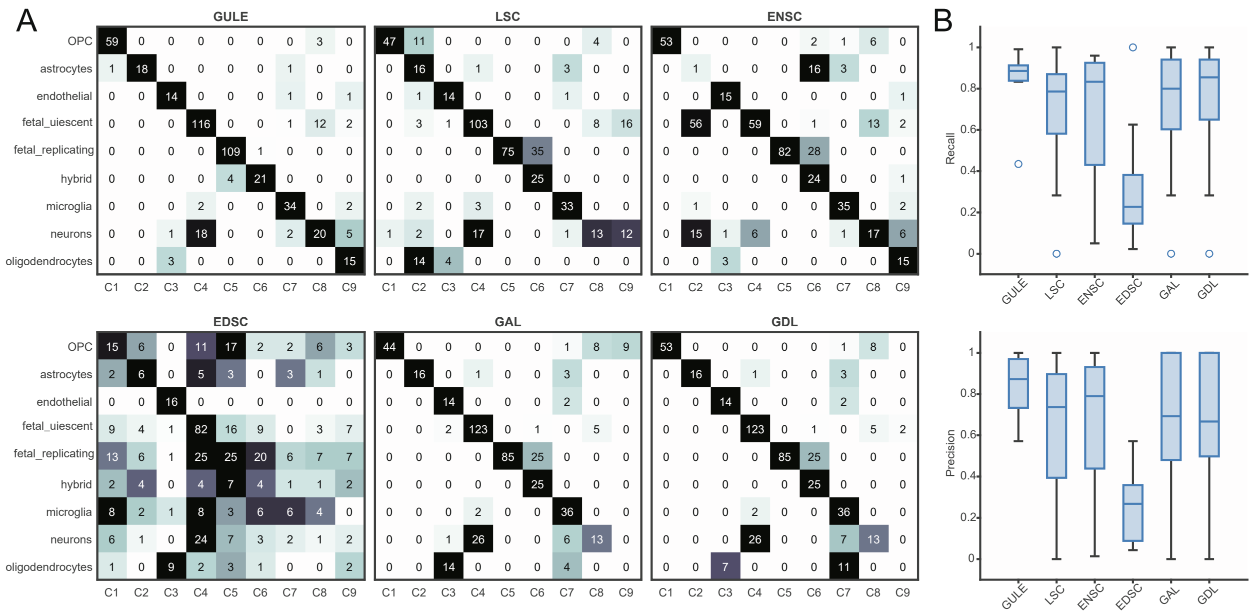

Figure S2: **Performance comparison of brain cell clustering methods on the Darmanis dataset.** (A). Confusion matrices of different clustering methods on the Darmanis human brain dataset containing 466 cells from nine major cell types. The rows represent true cell types and columns represent predicted clusters. (B) Recall and precision of each method.

Table S1: Characteristics of Datasets Used in Experimental Evaluation. Max CS = Maximum class size, Min CS = Minimum class size.

Data Set	Field	# Instance	# Features	# Classes	Max CS	Min CS	Distance Metric
Basehock	Text	1993	4862	2	999	994	Euclidean
COIL100	Object Image	7200	1024	100	72	72	Cityblock
COIL20	Object Image	1440	1024	20	72	72	Cityblock
Compounded	Synthetic	142	2	2	92	50	Cityblock
Control	Time seris	600	60	6	100	100	Euclidean
Entangled	Synthetic	312	2	3	106	101	Cityblock
Fashion	Fashion Image	70000	784	10	7000	7000	Spearman
HAR	Activity Recognition	10299	561	6	1944	1406	Euclidean
Iris	Plant	150	4	3	50	50	Euclidean
MNIST	Handwritten Digit	10000	784	10	1135	892	Cosine
MNIST70K	Handwritten Digit	70000	784	10	7877	6313	Cosine
MNIST-PT	Handwritten Digit	70000	500	10	7877	6313	Cosine
Olive	Face	400	4096	40	10	10	Euclidean
ORL	Face	400	10304	40	10	10	Euclidean
Pcmac	Text	1943	3289	2	982	961	Euclidean
Pendigit	Text	10992	16	10	1144	1055	Cityblock
PIE	Face	2856	1024	68	42	42	Cosine
Relathe	Text	1427	4322	2	779	648	Cityblock
Rounded	Synthetic	300	2	3	110	93	Cityblock
Satilite	remote sensing	6435	36	6	1533	626	Euclidean
Seed	Plant	210	7	3	70	70	Euclidean
Sports	Text	1000	59	2	635	365	Euclidean
UMist	Face	565	644	20	48	19	Cityblock
USPS	Handwritten Digit	9298	256	10	1553	708	Euclidean

Table S2: Web Sources of Datasets Used in Experimental Evaluation

Data Set	Website
Basehock	https://jundongl.github.io/scikit-feature/algorithms.html
COIL100	https://www.cs.columbia.edu/CAVE/software/softlib/coil-100.php
COIL20	https://www.cs.columbia.edu/CAVE/software/softlib/coil-20.php
Compounded	http://cs.joensuu.fi/sipu/datasets/
Control	https://archive.ics.uci.edu/ml/datasets/
Fashion	https://github.com/zalandoresearch/fashion-mnist
Entangled	http://cs.joensuu.fi/sipu/datasets/
HAR	https://archive.ics.uci.edu/ml/datasets/
Iris	https://archive.ics.uci.edu/ml/datasets/
MNIST	http://yann.lecun.com/exdb/mnist/
MNIST-P	http://vision.jhu.edu/code/
Olive	http://cam-orl.co.uk/facedatabase.html
ORL	http://cam-orl.co.uk/facedatabase.html
Pcmac	https://jundongl.github.io/scikit-feature/algorithms.html
Pendigit	https://archive.ics.uci.edu/ml/datasets/
PIE	http://www.cs.cmu.edu/afs/cs/project/PIE/MultiPi/Multi-Pie/Home.html
Relathe	https://jundongl.github.io/scikit-feature/algorithms.html
Rounded	http://cs.joensuu.fi/sipu/datasets/
Satilite	https://archive.ics.uci.edu/ml/datasets/
Seed	https://archive.ics.uci.edu/ml/datasets/
Sports	https://archive.ics.uci.edu/ml/datasets/
UMist	https://cs.nyu.edu/\textasciitilde roweis/data.html
USPS	https://web.stanford.edu/\textasciitilde hastie/StatLearnSparsity_files/DATA/zipcode.html

Table S3: Clustering performance (measured by ACC) of GULE and compared methods

Data Sets	GULE	SCSC	GAL	GDL	EDSC	EnSC	LSC	S-STSC	STSC	kmeans
Basehock	96.3	50.1	50.5	50.3	50.1	50.5	59.2	95.5	53.8	62.8
COIL100	94.3	73.2	69.1	70.5	47.0	57.3	50.7	66.1	46.1	52.0
COIL20	100.0	92.9	86.2	79.9	77.6	78.8	80.8	84.9	50.6	66.0
Control	97.5	49.7	83.3	92.3	53.2	52.7	79.3	66.0	55.5	56.8
HAR	76.3	64.3	55.2	18.9	35.6	67.5	71.7	63.6	51.0	70.6
Iris	96.0	86.7	90.0	69.3	52.7	97.3	89.3	96.7	90.7	96.7
MNIST	94.3	78.9	79.7	85.0	31.8	55.9	79.4	67.3	40.4	56.5
Olive	75.0	64.8	66.3	58.3	67.8	68.8	67.8	69.3	67.8	56.3
ORL	91.8	74.5	80.0	73.3	21.0	74.8	73.8	81.0	77.8	63.8
Pcmac	84.5	50.6	50.7	50.6	57.3	54.3	56.5	58.4	50.0	55.4
Pendigit	89.8	87.2	73.2	39.1	84.0	73.5	87.5	88.9	84.3	71.7
PIE	100.0	52.8	39.0	49.7	83.4	83.4	58.7	75.1	23.9	23.3
Relathe	77.9	54.4	54.7	54.7	54.7	54.7	55.3	81.7	51.1	57.7
Satilite	80.2	62.5	79.8	81.8	53.6	61.1	64.6	63.3	71.0	69.2
Seed	89.5	91.9	88.6	81.9	61.4	87.6	82.9	87.6	86.7	89.5
Sports	76.6	78.3	74.7	63.3	50.6	71.7	66.9	76.0	68.2	75.6
UMist	96.3	66.0	61.2	73.8	55.4	45.7	56.3	53.1	40.5	48.1
USPS	97.0	74.4	77.2	66.0	58.5	60.3	78.0	66.7	46.6	67.6
Average	89.6	69.6	70.0	64.4	55.3	66.4	69.9	74.5	58.7	63.3

Table S4: Clustering performance (measured by NMI) of GULE and compared methods

Data Sets	GULE	SCSC	GAL	GDL	EDSC	EnSC	LSC	S-STSC	STSC	kmeans
Basehock	77.3	0.1	0.4	0.2	0.1	0.2	9.1	75.3	0.4	5.0
COIL100	97.7	88.8	87.1	90.2	73.6	81.4	75.3	86.1	72.9	76.3
COIL20	100.0	95.6	91.3	90.7	86.2	91.2	88.4	91.4	68.8	78.8
Control	92.6	58.1	76.6	87.1	63.4	63.2	72.4	70.5	70.2	74.2
HAR	71.1	65.6	59.8	0.1	33.2	71.5	73.7	65.5	56.7	67.6
Iris	87.0	74.6	78.7	59.7	57.9	91.3	74.0	89.8	77.9	89.8
MNIST	87.8	79.8	76.0	81.8	24.4	61.3	74.7	73.4	41.5	54.2
Olive	86.4	80.4	81.6	77.8	83.3	83.9	81.6	81.7	82.1	71.6
ORL	94.9	86.1	91.4	88.7	38.3	88.0	88.1	90.5	89.2	82.3
Pcmac	38.0	0.1	0.2	0.1	1.5	3.2	1.8	7.4	0.2	1.7
Pendigit	87.6	83.8	77.4	39.8	76.8	74.5	82.8	85.2	79.3	67.5
PIE	100.0	65.9	61.9	71.7	94.8	96.1	79.1	88.3	48.1	51.9
Relathe	23.7	0.1	0.0	0.2	0.2	0.0	2.5	33.9	0.0	1.2
Satilite	64.9	61.8	64.3	64.8	41.7	55.5	61.4	64.7	58.6	61.1
Seed	69.1	73.3	69.5	60.7	27.2	65.4	58.3	65.4	65.4	70.0
Sports	22.2	24.6	17.5	0.1	0.0	19.4	11.4	23.2	16.3	17.6
UMist	96.4	79.3	78.7	86.5	71.2	66.2	77.7	75.5	64.9	70.2
USPS	92.1	75.6	81.7	70.2	63.5	69.4	79.9	81.5	52.5	61.3
Average	77.2	60.7	60.8	53.9	46.5	60.1	60.7	69.4	52.5	55.7

Table S5: Clustering performance (measured by ARI) of GULE and compared methods

Data Sets	GULE	SCSC	GAL	GDL	EDSC	EnSC	LSC	S-STSC	STSC	kmeans
Basehock	85.9	0.0	0.0	0.0	0.0	0.0	3.3	82.9	0.5	6.5
COIL100	92.6	62.9	64.6	66.9	38.2	51.7	43.0	59.7	38.6	46.2
COIL20	100.0	90.4	82.6	72.2	72.7	77.4	75.1	81.3	42.3	61.8
Control	94.1	44.1	68.3	83.9	51.4	49.3	61.6	58.6	59.4	61.7
HAR	61.9	57.4	52.0	0.0	23.1	60.7	66.7	59.3	39.4	62.8
Iris	88.6	68.4	74.6	56.1	44.3	92.2	72.9	90.4	75.8	90.4
MNIST	88.0	73.5	71.2	79.2	12.4	45.2	68.3	61.9	26.1	42.2
Olive	65.7	50.1	53.5	43.8	56.7	59.3	53.9	57.4	56.0	37.0
ORL	85.8	61.6	75.3	67.8	5.9	67.9	67.6	74.0	71.3	54.2
Pcmac	47.6	0.0	0.0	0.0	2.1	0.7	1.7	2.8	0.0	1.1
Pendigit	81.5	75.3	62.3	20.2	70.9	61.7	76.9	79.3	70.1	57.0
PIE	100.0	21.8	22.6	34.4	82.9	86.1	49.7	68.7	13.4	13.9
Relathe	31.1	0.0	0.1	0.1	0.1	0.0	0.8	40.1	0.0	2.2
Satilite	65.8	53.1	66.9	68.0	36.8	49.0	51.5	54.1	49.4	53.6
Seed	72.1	77.7	70.2	56.6	27.5	67.6	55.7	67.0	65.0	71.7
Sports	28.2	31.9	24.2	0.0	0.0	18.7	11.3	27.0	12.9	25.9
UMist	93.7	52.8	56.2	69.8	41.0	35.9	48.5	47.7	31.9	40.4
USPS	94.1	62.4	73.3	63.9	47.8	50.1	73.2	67.7	34.7	53.6
Average	76.5	49.1	51.0	43.5	34.1	48.5	49.0	60.0	38.2	43.5

Table S6: Clustering accuracy ACC (%) of GULE and the deep networks methods. Blank in the table indicate that the dataset was not tested by the corresponding method.

Method	COIL-20	COIL-100	PIE	ORL	UMist	USPS	MNIST-T	Pendigit	MNIST	Fashion
JULE	100.0	91.6	100.0		80.9	95.0	96.1			
DSC	94.9	69.0		86.0						
DBC	79.3	77.5				74.3				
DDM			85.8			97.9	87.1		96.9	
GALA	82.3						74.3			
N2D						95.8	94.8	88.5		
DASC	96.4			88.3	76.9					
EDAE						87.3		87.3		
DSCNSS	96.3	71.4		89.2						
SADSC	97.5	84.5		91.3			88.3			
DCRSM	81.2	77.0								69.7
ODSC	97.5			88.0			81.2			
WEC	84.0								96.7	62.3
DCCF						85.5			97.4	62.1
DFCN						79.5				
EDESC										63.1
DCMF						79.7	87.8		90.2	61.7
DEC									84.3	60.0
LGC-AUM						97.5			98.7	65.4
RD-FKC	73.5		37.7	65.4		76.1				61.2
DPSC			82.4			80.0	97.6		97.6	
DeepGMM	94.6	86.3	95.7			98.5			98.7	63.5
DSCSC	97.9			90.8	81.8				85.1	
DSC-DAG	97.3	72.1		91.1	78.1					
GULE	100.0	96.0	100.0	93.0	96.5	97.1	94.5	89.8	95.6	66.4

Table S7: Stability of parameter k_0 on clustering accuracy

Data Sets	$k_0 = 0$	1	2	3	4	5	6	7	8	9	10
Basehock	95.6	95.9	96.2	96.6	96.7	96.3	96.6	96.8	96.8	96.5	96.9
COIL100	94.5	94.8	93.4	94.8	95.6	94.3	96.8	94.8	89.9	96.3	83.7
COIL20	99.7	100.0	93.5	100.0	100.0	100.0	100.0	100.0	100.0	99.9	100.0
Compounded	85.2	99.3	100.0	61.3	100.0	100.0	100.0	100.0	100.0	100.0	100.0
Control	67.0	76.7	69.3	90.0	96.3	97.5	97.7	98.3	98.0	97.8	97.2
Entangled	100.0	100.0	100.0	100.0	100.0	100.0	100.0	100.0	100.0	100.0	95.2
HAR	81.4	80.4	81.9	79.7	78.1	76.3	76.5	76.4	75.2	74.2	72.4
Iris	96.0	96.0	96.0	96.0	96.0	96.0	96.7	90.7	90.0	96.7	96.0
MNIST	93.6	93.8	93.9	93.9	94.2	94.3	94.4	93.0	91.4	90.2	90.4
Olive	65.5	71.5	70.0	73.3	75.0	75.0	74.0	72.8	73.0	72.5	74.0
ORL	73.8	83.3	84.5	88.0	89.0	91.8	92.0	86.5	87.3	87.5	87.5
Pcmac	81.7	83.4	84.7	85.3	84.4	84.5	85.3	85.7	85.9	86.3	86.1
Pendigit	89.6	89.7	89.6	89.8	89.9	89.8	89.9	89.8	90.0	89.8	89.8
PIE	94.5	95.1	97.5	99.2	99.9	100.0	100.0	100.0	100.0	100.0	98.5
Relathe	78.8	78.4	78.8	77.8	77.6	77.9	77.7	78.0	77.4	77.2	77.2
Rounded	71.0	98.3	90.3	82.3	72.0	99.3	88.7	88.7	88.7	88.7	88.7
Satilite	60.1	62.1	78.4	75.3	81.2	80.2	79.3	80.9	79.9	69.2	65.7
Seed	79.5	77.1	87.6	87.6	88.6	89.5	89.5	89.5	88.6	87.6	88.1
Sports	76.2	76.3	76.2	76.6	76.6	76.6	76.7	76.5	76.0	76.1	76.4
UMist	82.3	86.5	88.7	87.1	90.3	96.3	97.2	93.5	95.9	93.5	96.5
USPS	96.8	96.7	96.8	97.0	97.0	97.0	96.9	96.9	96.8	96.8	96.9
Average	83.9	87.4	88.0	87.2	89.4	91.1	90.8	89.9	89.6	89.4	88.4

Table S8: Stability of parameter α on clustering accuracy

Data Sets	$\alpha = 0$	1	2	3	4	5	6	7	8	9	10	11	12	13	14	15
Basehock	95.9	96.0	96.2	95.9	97.1	97.1	96.3	94.8	86.7	67.8	57.0	51.9	50.4	52.9	51.0	50.7
COIL100	68.2	66.7	69.9	74.6	87.1	91.4	94.7	95.9	94.3	94.3	96.0	87.3	78.0	59.7	44.0	24.3
COIL20	84.5	84.4	86.5	88.4	92.4	100.0	100.0	100.0	100.0	100.0	100.0	87.1	99.4	86.9	73.9	65.9
Compounded	57.0	57.0	57.7	57.0	56.3	100.0	100.0	100.0	100.0	100.0	100.0	100.0	100.0	98.6	97.9	98.6
Control	54.3	70.2	71.2	71.0	73.5	80.7	83.2	90.2	98.5	97.5	35.0	43.5	35.5	30.7	31.2	33.5
Entangled	38.8	46.8	65.7	67.6	76.3	100.0	100.0	100.0	100.0	100.0	100.0	100.0	100.0	100.0	100.0	99.7
HAR	70.4	70.3	70.6	72.3	74.8	76.0	76.3	78.2	75.6	61.1	42.0	50.4	39.1	41.4	45.3	31.7
Iris	90.0	90.0	90.7	90.7	92.7	96.0	96.0	96.0	96.0	96.0	96.0	96.0	96.0	64.7	75.3	73.3
MNIST	81.9	80.6	87.4	70.2	78.9	83.4	94.3	94.7	92.9	91.3	35.0	24.3	24.6	23.9	24.5	24.4
Olive	67.0	67.3	69.3	70.5	72.3	73.8	75.3	75.5	75.5	75.0	68.0	56.3	56.0	54.0	53.0	53.3
ORL	78.3	79.5	81.3	83.0	84.3	87.0	88.5	88.8	88.3	91.8	78.8	62.5	64.3	65.3	61.0	57.5
Pcmac	72.7	73.5	79.7	83.2	88.3	86.4	84.5	82.9	74.9	73.3	51.3	50.3	51.0	51.2	51.0	50.0
Pendigit	88.5	89.0	88.8	89.6	89.4	89.5	89.8	89.8	89.6	89.7	88.7	74.1	47.0	43.5	36.5	26.6
PIE	98.9	99.1	99.2	99.8	100.0	100.0	100.0	100.0	100.0	100.0	96.5	99.4	99.7	82.5	53.2	40.2
Relathe	77.6	78.8	80.2	80.7	81.0	80.5	77.9	73.4	59.1	78.8	54.2	50.1	51.2	51.4	50.7	51.2
Rounded	80.0	80.0	80.7	84.3	99.0	96.0	98.3	98.7	99.0	99.3	98.0	98.0	98.3	98.0	98.0	88.3
Satilite	65.7	65.4	65.6	66.4	61.5	61.5	80.2	56.5	71.1	63.5	55.5	40.6	34.7	34.2	32.2	38.8
Seed	92.9	92.4	92.4	91.9	91.4	91.0	90.0	89.5	89.5	89.5	89.5	89.5	71.0	66.2	44.8	53.8
Sports	76.5	76.2	76.2	76.0	76.5	76.3	76.6	76.8	75.4	74.5	73.4	56.6	53.3	53.7	51.6	51.3
UMist	56.8	58.8	63.0	72.0	82.8	83.4	88.7	90.6	91.0	96.3	95.4	93.8	80.5	69.2	48.7	45.3
USPS	78.6	78.6	78.8	79.8	80.8	96.7	97.0	96.7	80.0	96.4	69.6	35.6	31.9	31.4	35.2	27.7
Average	75.0	76.2	78.6	79.3	82.7	87.9	89.9	89.0	87.5	87.4	75.2	68.9	64.9	60.0	55.2	51.7

Table S9: Stability of parameter β on clustering accuracy

Data Sets	$\beta = 0$	0.1	0.2	0.3	0.4	0.5	0.6	0.7	0.8	0.9	1
Basehock	96.3	96.3	96.3	96.3	96.3	96.3	96.3	96.3	96.3	96.3	96.4
COIL100	94.3	94.6	94.7	94.6	94.8	94.8	94.4	94.0	92.9	92.2	89.4
COIL20	100.0	100.0	100.0	100.0	100.0	100.0	100.0	100.0	100.0	100.0	100.0
Compounded	100.0	100.0	100.0	100.0	100.0	100.0	100.0	100.0	100.0	100.0	100.0
Control	97.5	97.5	97.5	97.3	97.3	97.3	97.5	97.5	97.5	97.5	97.5
Entangled	100.0	100.0	100.0	100.0	100.0	100.0	100.0	100.0	100.0	100.0	100.0
HAR	76.3	76.2	76.2	76.2	76.1	76.1	76.1	76.1	76.0	73.1	52.8
Heart	71.6	71.6	71.6	71.6	71.6	71.6	71.6	71.6	71.6	71.6	54.8
Iris	96.0	96.0	96.0	96.0	96.0	96.0	96.0	96.0	96.0	96.0	96.0
MNIST	94.3	94.2	94.2	94.3	94.2	94.3	94.2	94.3	94.3	90.8	84.6
Olive	75.0	75.0	75.0	76.3	75.3	75.3	73.5	73.3	73.3	73.0	72.5
ORL	91.8	91.8	91.8	91.8	91.0	91.0	91.0	89.0	89.0	89.0	89.0
Pcmac	84.5	84.5	84.5	84.5	84.5	84.5	84.6	84.6	84.6	84.6	84.6
Pendigit	89.8	89.8	89.8	89.8	89.8	89.8	89.8	89.8	89.8	90.0	73.3
PIE	100.0	100.0	100.0	100.0	100.0	100.0	100.0	100.0	100.0	100.0	100.0
Relathe	78.0	78.0	78.1	78.1	78.1	78.1	78.1	78.1	78.3	78.3	78.3
Rounded	99.3	99.3	99.3	99.3	99.3	99.3	99.3	99.3	99.3	78.3	78.3
Satilite	80.2	80.3	80.3	68.5	68.5	68.4	68.4	67.7	56.1	55.7	56.2
Seed	89.5	89.5	89.5	89.5	89.5	89.5	89.5	89.5	89.5	89.5	89.5
Sports	76.6	76.6	76.6	76.6	76.6	76.6	76.5	76.5	76.6	76.6	76.6
UMist	95.6	95.6	95.8	95.8	95.8	95.8	95.8	92.4	92.4	92.4	92.4
USPS	97.0	97.0	97.0	97.0	97.0	97.0	97.0	97.0	97.0	97.0	97.0
Average	90.2	90.2	90.2	89.7	89.6	89.6	89.5	89.2	88.7	87.4	84.5

Table S10: Performance of distance selection. NSE = normalized squared Euclidean. NE = normalized Euclidean.

Data	Euclidean	NSE	Cityblock	Spearman	NE	Best Accuracy	Selected Accuracy	Gap to Best	Selected Distance
COIL100	0.86	0.86	0.94	0.81	0.88	0.94	0.94	0	Cityblock
COIL20	0.73	0.9	1	0.76	0.92	1	1	0	Cityblock
Control	0.98	0.75	0.92	0.66	0.98	0.98	0.98	0	Euclidean
HAR	0.65	0.76	0.47	0.46	0.76	0.76	0.76	0	NE
Iris	0.96	0.85	0.96	0.79	0.86	0.96	0.96	0	Euclidean
MNIST	0.88	N/A	0.92	0.92	0.94	0.94	0.94	0	NE
Olive	0.69	0.75	0.73	0.72	0.68	0.75	0.69	0.07	Euclidean
ORL	0.92	0.81	0.87	0.83	0.81	0.92	0.92	0	Euclidean
Pendigit	0.89	0.87	0.9	0.85	0.89	0.9	0.89	0.01	Euclidean
PIE	0.77	1	0.66	0.96	1	1	1	0	NSE
Satilite	0.8	0.57	0.68	0.45	0.5	0.8	0.8	0	Euclidean
Seed	0.81	0.9	0.8	0.61	0.88	0.9	0.9	0	NSE
UMist	0.87	0.86	0.96	0.88	0.79	0.96	0.87	0.09	Euclidean
USPS	0.97	0.96	0.8	0.79	0.97	0.97	0.97	0	Euclidean
Average	0.84	0.83	0.83	0.75	0.85	0.91	0.9	0.01	

References

1. Lloyd, S. (1982). Least squares quantization in pcm. IEEE transactions on information theory *28*, 129–137. https://doi.org/10.1109/TIT.1982.1056489.
2. Arthur, D., and Vassilvitskii, S. (2007). K-means++ the advantages of careful seeding. In Proceedings of the eighteenth annual ACM-SIAM symposium on Discrete algorithms. pp. 1027–1035. https://dl.acm.org/doi/10.5555/1283383.1283494.
3. Zelnik-Manor, L., and Perona, P. (2004). Self-tuning spectral clustering. Advances in neural information processing systems *17*. https://proceedings.neurips.cc/paper_files/paper/2004/file-/40173ea48d9567f1f393b20c855bb40b-Paper.pdf.
4. Kuang, D., Yun, S., and Park, H. (2015). Symnmf: nonnegative low-rank approximation of a similarity matrix for graph clustering. Journal of Global Optimization *62*, 545–574. https://doi.org/10.1007/s10898-014-0247-2.
5. Cai, D., and Chen, X. (2014). Large scale spectral clustering via landmark-based sparse representation. IEEE transactions on cybernetics *45*, 1669–1680. https://doi.org/10.1109/TCYB.2014.2358564.
6. Bai, L., Liang, J., and Zhao, Y. (2022). Self-constrained spectral clustering. IEEE Transactions on Pattern Analysis and Machine Intelligence *45*, 5126–5138. https://doi.org/10.1109/TPAMI.2022.3188160.
7. You, C., Li, C.G., Robinson, D.P., and Vidal, R. (2016). Oracle based active set algorithm for scalable elastic net subspace clustering. In Proceedings of the IEEE conference on computer vision and pattern recognition. pp. 3928–3937. https://openaccess.thecvf.com/content_cvpr-_2016/papers/You_Oracle_Based_Active_CVPR_2016_paper.pdf.
8. Ji, P., Salzmann, M., and Li, H. (2014). Efficient dense subspace clustering. In IEEE Winter conference on applications of computer vision. IEEE pp. 461–468. https://doi.org/10.1109/WACV.2014.6836065.
9. Zhang, W., Wang, X., Zhao, D., and Tang, X. (2012). Graph degree linkage: Agglomerative clustering on a directed graph. In Computer Vision–ECCV 2012: 12th European Conference on Computer Vision, Florence, Italy, October 7-13, 2012, Proceedings, Part I 12. Springer pp. 428–441. https://doi.org/10.1007/978-3-642-33718-5_31.
10. Yang, J., Parikh, D., and Batra, D. (2016). Joint unsupervised learning of deep representations and image clusters. In Proceedings of the IEEE conference on computer vision and pattern recognition. pp. 5147–5156. https://www.cv-foundation.org/openaccess/content_cvpr_2016/papers/Yang_Joint_Unsupervised_Learning_CVPR-_2016_paper.pdf.
11. Ji, P., Zhang, T., Li, H., Salzmann, M., and Reid, I. (2017). Deep subspace clustering networks. Advances in neural information processing systems *30*. https://proceedings.neurips.cc/paper_files/paper/2017/file/e369853df766fa44e1ed0ff613f563bdPaper.pdf.
12. Zhou, P., Hou, Y., and Feng, J. (2018). Deep adversarial subspace clustering. In Proceedings of the IEEE conference on computer vision and pattern recognition. pp. 1596–1604. https://openaccess.thecvf.com/content_cvpr_2018/papers/Zhou_Deep_Adversarial_Subspa-ce_CVPR_2018_paper.pdf.
13. Li, F., Qiao, H., and Zhang, B. (2018). Discriminatively boosted image clustering with fully convolutional auto-encoders. Pattern Recognition *83*, 161–173. https://doi.org/10.1016/j.patcog.2018.05.019.
14. Jabi, M., Pedersoli, M., Mitiche, A., and Ayed, I.B. (2019). Deep clustering: On the link between discriminative models and k-means. IEEE transactions on pattern analysis and machine intelligence *43*, 1887–1896. https://doi.org/10.1109/TPAMI.2019.2962683.
15. Park, J., Lee, M., Chang, H.J., Lee, K., and Choi, J.Y. (2019). Symmetric graph convolutional autoencoder for unsupervised graph representation learning. In Proceedings of the IEEE/CVF international conference on computer vision. pp. 6519–6528. https://openaccess.thecvf.com/content_ICCV_2019/papers/Park_Symmetric_Graph_Convolutional-_Autoencoder_for_Unsupervised_Graph_Representation_Learning_ICCV_2019_paper.pdf.
16. McConville, R., Santos-Rodriguez, R., Piechocki, R.J., and Craddock, I. (2021). N2d:(not too) deep clustering via clustering the local manifold of an autoencoded embedding. In 2020 25th international conference on pattern recognition (ICPR). IEEE pp. 5145–5152. https://doi.org/10.1109/ICPR48806.2021.9413131.

17. Affeldt, S., Labiod, L., and Nadif, M. (2020). Spectral clustering via ensemble deep autoencoder learning (sc-edae). Pattern Recognition *108*, 107522. https://doi.org/10.1016/j.patcog.2020.107522.

18. Chen, C., Lu, H., Wei, H., and Geng, X. (2023). Deep subspace image clustering network with self-expression and self-supervision. Applied Intelligence *53*, 4859–4873. https://doi.org/10.1007/s10489-022-03654-6.

19. Chen, Z., Ding, S., and Hou, H. (2021). A novel self-attention deep subspace clustering. International Journal of Machine Learning and Cybernetics *12*, 2377–2387. https://doi.org/10.1007/s13042-021-01318-4.

20. Shi, Z., and Zhao, H. (2023). Deep multi-view clustering based on reconstructed self-expressive matrix. Applied Sciences *13*, 8791. https://doi.org/10.3390/app13158791.

21. Valanarasu, J.M.J., and Patel, V.M. (2021). Overcomplete deep subspace clustering networks. In Proceedings of the IEEE/CVF winter conference on applications of computer vision. pp. 746–755. https://openaccess.thecvf.com/content/WACV2021/papers/Valanarasu_Overcomplete_Deep_Subspace_Clustering_Networks_WACV_2021_paper.pdf.

22. Cai, J., Zhang, Y., Wang, S., Fan, J., and Guo, W. (2024). Wasserstein embedding learning for deep clustering: A generative approach. IEEE Transactions on Multimedia. https://doi.org/10.1109/TMM.2024.3369862.

23. Cai, J., Wang, S., Xu, C., and Guo, W. (2022). Unsupervised deep clustering via contractive feature representation and focal loss. Pattern Recognition *123*, 108386. https://doi.org/10.1016/j.patcog.2021.108386.

24. Tu, W., Zhou, S., Liu, X., Guo, X., Cai, Z., Zhu, E., and Cheng, J. (2021). Deep fusion clustering network. In Proceedings of the AAAI Conference on Artificial Intelligence vol. 35. pp. 9978–9987. https://doi.org/10.1609/aaai.v35i11.17198.

25. Cai, J., Fan, J., Guo, W., Wang, S., Zhang, Y., and Zhang, Z. (2022). Efficient deep embedded subspace clustering. In Proceedings of the IEEE/CVF Conference on Computer Vision and Pattern Recognition. pp. 1–10. https://doi.ieeecomputersociety.org/10.1109/CVPR52688.2022.00012.

26. Hou, H., Ding, S., and Xu, X. (2022). A deep clustering by multi-level feature fusion. International Journal of Machine Learning and Cybernetics *13*, 2813–2823. https://doi.org/10.1007/s13042-022-01557-z.

27. Wickramasinghe, C., Marino, D., and Manic, M. (2021). Deep embedded clustering with resnets. In 2021 14th International Conference on Human System Interaction (HSI). IEEE pp. 1–6. https://doi.org/10.1109/HSI52170.2021.9538747.

28. Wang, T., Zhang, X., Lan, L., and Luo, Z. (2022). Local-to-global deep clustering on approximate uniform manifold. IEEE Transactions on Knowledge and Data Engineering *35*, 5035–5046. https://doi.org/10.1109/TKDE.2022.3144952.

29. Wu, X., Yu, Y.F., Chen, L., Ding, W., and Wang, Y. (2024). Robust deep fuzzy k-means clustering for image data. Pattern Recognition *153*, 110504. https://doi.org/10.1016/j.patcog.2024.110504.

30. Hu, W., Chen, C., Ye, F., Zheng, Z., and Du, Y. (2021). Learning deep discriminative representations with pseudo supervision for image clustering. Information Sciences *568*, 199–215. https://doi.org/10.1016/j.ins.2021.03.066.

31. Wang, J., and Jiang, J. (2021). Unsupervised deep clustering via adaptive gmm modeling and optimization. Neurocomputing *433*, 199–211. https://doi.org/10.1016/j.neucom.2020.12.082.

32. Peng, B., and Zhu, W. (2021). Deep structural contrastive subspace clustering. In Asian Conference on Machine Learning. PMLR pp. 1145–1160. https://proceedings.mlr.press/v157/peng21a/peng21a.pdf.

33. Yu, Z., Zhang, Z., Cao, W., Liu, C., Chen, C.P., and Wong, H.S. (2020). Gan-based enhanced deep subspace clustering networks. IEEE Transactions on Knowledge and Data Engineering *34*, 3267–3281. https://doi.org/10.1109/TKDE.2020.3025301.

34. Li, J., Cheng, K., Wang, S., Morstatter, F., Trevino, R.P., Tang, J., and Liu, H. (2018). Feature selection: A data perspective. ACM Computing Surveys (CSUR) *50*, 94. https://doi.org/10.1145/3136625.

35. Nene, S.A., Nayar, S.K., and Murase, H. (1996). Columbia object image library. Columbia University. https://www.cs.columbia.edu/CAVE/software/softlib/coil-20.php.

36. Alcock, R.J., Manolopoulos, Y. et al. (1999). Time-series similarity queries employing a feature-based approach. In 7th Hellenic conference on informatics. pp. 27–29. https://machinelearning101.pbworks.com/f/TimeSeriesData10.1.1.79.1572.pdf.

37. Xiao, H., Rasul, K., and Vollgraf, R. (2017). Fashion-mnist: a novel image dataset for benchmarking machine learning algorithms. Preprint at arXiv. https://doi.org/10.48550/arXiv.1708.07747.

38. Anguita, D., Ghio, A., Oneto, L., Parra, X., and Reyes-Ortiz, J.L. (2013). A public domain dataset for human activity recognition using smartphones. In 21th European Symposium on Artificial Neural Networks, Computational Intelligence and Machine Learning, ESANN 2013. ESANN pp. 437–442. http://hdl.handle.net/2117/20897.

39. Fisher, R.A. (1936). The use of multiple measurements in taxonomic problems. Annals of eugenics *7*, 179–188. https://doi.org/10.1111/j.1469-1809.1936.tb02137.x.

40. LeCun, Y., Bottou, L., Bengio, Y., and Haffner, P. (1998). Gradient-based learning applied to document recognition. Proceedings of the IEEE *86*, 2278–2324. https://doi.org/10.1109/5.726791.

41. Samaria, F.S., and Harter, A.C. (1994). Parameterisation of a stochastic model for human face identification. In Proceedings of 1994 IEEE Workshop on Applications of Computer Vision. IEEE pp. 138–142. https://doi.org/10.1109/ACV.1994.341300.

42. Alimoglu, F., and Alpaydin, E. (1996). Combining multiple classifiers for pen-based handwritten digit recognition. TAINN Proceedings pp. 38–41. https://doi.org/10.1109/ICDAR.1997.620583.

43. Sim, T., Baker, S., and Bsat, M. (2002). The cmu pose, illumination, and expression (pie) database. In Proceedings of Fifth IEEE International Conference on Automatic Face Gesture Recognition. IEEE pp. 53–58. https://doi.org/10.1109/AFGR.2002.1004130.

44. Srinivasan, A. (1993). Statlog (Landsat Satellite). UCI Machine Learning Repository. . https://doi.org/10.24432/C55887.

45. Charytanowicz, M., Niewczas, J., Kulczycki, P., Kowalski, P., and Lukasik, S. (2010). Seeds. UCI Machine Learning Repository. . https://doi.org/10.24432/C5H30K.

46. Rizk, Y., and Awad, M. (2018). Sports articles for objectivity analysis. UCI Machine Learning Repository. . https://doi.org/10.24432/C5801R.

47. Graham, D.B., and Allinson, N.M. (1998). Characterising virtual eigensignatures for general purpose face recognition. In Face Recognition: From Theory to Applications pp. 446–456.. Springer pp. 446–456. https://link.springer.com/chapter/10.1007/978-3-642-72201-1_25.

48. Hull, J.J. (1994). A database for handwritten text recognition research. IEEE Transactions on pattern analysis and machine intelligence *16*, 550–554. https://doi.org/10.1109/34.291440.

49. Schur, J. (1905). Zur theorie der vertauschbaren matrizen. Journal für die reine und angewandte Mathematik *1905*, 66–76. https://doi.org/10.1515/crll.1905.130.66.

50. Golub, G.H., and Van Loan, C.F. (2013). Matrix computations. JHU press. https://epubs.siam.org/doi/abs/10.1137/1.9781421407944.

51. Rand, W.M. (1971). Objective criteria for the evaluation of clustering methods. Journal of the American Statistical association *66*, 846–850. https://doi.org/10.1080/01621459.1971.10482356.

52. Pfitzner, D., Leibbrandt, R., and Powers, D. (2009). Characterization and evaluation of similarity measures for pairs of clusterings. Knowledge and Information Systems *19*, 361–394. https://doi.org/10.1007/s10115-008-0150-6.

53. Rousseeuw, P.J. (1987). Silhouettes: a graphical aid to the interpretation and validation of cluster analysis. Journal of computational and applied mathematics *20*, 53–65. https://doi.org/10.1016/0377-0427(87)90125-7.

54. Kandel, E.R., Schwartz, J.H., and Jessell, T.M. (2013). Principles of Neural Science. 5th ed.. McGraw-Hill. https://neurology.mhmedical.com/book.aspx?bookid=3024.

55. Albin, R.L., Young, A.B., and Penney, J.B. (1989). The functional anatomy of basal ganglia disorders. Trends in Neurosciences *12*, 366–375. https://doi.org/10.1016/0166-2236(89)90074-X.

56. DeLong, M.R. (1990). Primate models of movement disorders of basal ganglia origin. Trends in Neurosciences *13*, 281–285. https://doi.org/10.1016/0166-2236(90)90110-V.

57. Schmahmann, J.D. (2019). The cerebellum and cognition. Neuroscience Letters *688*, 62–75. https://doi.org/10.1016/j.neulet.2018.07.005.

58. LeDoux, J. (2000). Emotion circuits in the brain. Annual Review of Neuroscience *23*, 155–184. https://doi.org/10.4324/9780203825266.

59. Squire, L.R., and Zola-Morgan, S. (1991). The medial temporal lobe memory system. Science *253*, 1380–1386. https://doi.org/10.1126/science.1896849.

60. Saper, C.B., Chou, T.C., and Scammell, T.E. (2001). The sleep switch: hypothalamic control of sleep and wakefulness. Trends in Neurosciences *24*, 726–731. https://doi.org/10.1016/S0166-2236(00)02002-6.

61. Obeso, J.A., Rodriguez-Oroz, M.C., Benitez-Temino, B. et al. (2008). Functional organization of the basal ganglia: Therapeutic implications for parkinson's disease. Movement Disorders *23*, S548–S559. https://doi.org/10.1002/mds.22062.

62. Zeisel, A., Muñoz-Manchado, A.B., Codeluppi, S., Lönnerberg, P., La Manno, G., Jureus, A., Marques, S., Munguba, H., He, L., Betsholtz, C., Rolny, C., Castelo-Branco, G., Hjerling-Leffler, J., and Linnarsson, S. (2015). Cell types in the mouse cortex and hippocampus revealed by single-cell rna-seq. Science *347*, 1138–1142. https://doi.org/10.1126/science.aaa1934.

63. Darmanis, S., Sloan, S.A., Zhang, Y., Enge, S., Caneda, C., Shuer, L., Gephart, M.G., and Barres, B.A. (2015). A survey of human brain transcriptome diversity at the single cell level. Proceedings of the National Academy of Sciences *112*, 7285–7290. https://doi.org/10.1073/pnas.1507125112.
