# Supplementary material for: Global understanding via local extraction for data clustering and visualization
Source: Patterns (N Y). 2025 May 19;6(9):101266. doi: 10.1016/j.patter.2025.101266 (PMC12485538; doi:10.1016/j.patter.2025.101266)
Supplement: Document S2. Article plus supplemental information [file mmc2.pdf]

Patterns

Article

Global understanding via local extraction for data clustering and visualization

Highlights

- GULE extracts weak local consistency from complex unlabeled data
- Two-layer architecture enhances class consistency through iterative self-learning
- Cell types and functions in RNA-seq data are accurately identified at high precision
- Topological structures within classes are preserved in data visualization

Authors

Zhenyue Zhang, Bingjie Li

Correspondence

zyzhang@zju.edu.cn (Z.Z.),
bjlistat@nus.edu.sg (B.L.)

In brief

Extracting meaningful patterns from unlabeled data remains challenging across scientific disciplines. Zhang and Li introduce GULE, a framework that identifies local class consistency and propagates it globally. GULE outperforms existing clustering methods across diverse datasets and achieves exceptional precision in identifying cell types from RNA-seq data. The method also preserves topological structures within classes during visualization, offering researchers deeper insights into complex data relationships.

Zhang & Li, 2025, Patterns *6*, 101266
September 12, 2025 © 2025 The Author(s). Published by Elsevier Inc.
https://doi.org/10.1016/j.patter.2025.101266

CellPress

Patterns

CellPress
OPEN ACCESS

Article

Global understanding via local extraction for data clustering and visualization

Zhenyue Zhang[1,2,*] and Bingjie Li[3,4,*]
[1]MSU-BIT-SMBU Joint Research Center of Applied Mathematics, Shenzhen MSU-BIT University, Shenzhen, China
[2]School of Mathematical Sciences, Zhejiang University, Hangzhou, China
[3]Department of Statistics and Data Science, National University of Singapore, Singapore, Singapore
[4]Lead contact
*Correspondence: zyzhang@zju.edu.cn (Z.Z.), bjlistat@nus.edu.sg (B.L.)
https://doi.org/10.1016/j.patter.2025.101266

THE BIGGER PICTURE The discovery of meaningful patterns in complex unlabeled data remains a significant challenge in science. Traditional clustering methods often struggle with real-world data that contain varied structures or densities or high dimensionality. Our GULE framework addresses these limitations by focusing on local relationships between data points and strengthening these connections to reveal global patterns. This approach is particularly valuable in biological applications such as single-cell RNA sequencing, where GULE can distinguish cell types with high precision. By preserving topological structure within each class, GULE also enhances data visualization, allowing researchers to understand relationships within groups. This robust framework adapts to each dataset's unique characteristics, potentially accelerating discoveries across multiple scientific domains.

SUMMARY

Retrieving latent class patterns from complex data is challenging. This paper focuses on the problem of retrieving latent classes from local connections of raw data without any assumptions regarding data structures or distributions. We propose a framework called GULE (global understanding via local extraction) to address this challenge through both local extraction of class consistency and global propagation of the identified consistency. This paper provides a series of theoretical analyses to show why the GULE algorithm can retrieve latent classes with high accuracy. GULE can also serve as a tool for data visualization to preserve class topology structures. Comprehensive testing demonstrates that GULE provides precise clustering and highly reliable visualizations, potentially offering insights into diverse applications, including biology and medicine.

INTRODUCTION

Finding latent class patterns in unlabeled complex data is challenging across many applications. The difficulties include heterogeneous data modalities (images, text, audio, time series, handwritten characters, biological sequences), structural complexities (non-uniform distributions, varying sampling densities, topological intricacies), inherent noise, and the curse of dimensionality. Unsupervised clustering and visualization are two commonly used tools for addressing the challenges: the former aims to uncover latent classes and enable automated pattern discovery, while the latter provides an intuitive means of understanding data structures. Traditionally, pattern identification techniques have been employed for tasks such as image segmentation,[1,2] object recognition,[3] speech separation,[4] and time series data recognition.[5] To handle these tasks, various algorithms have been developed, including k-means,[6] graph clustering,[7,8] matrix factorization,[9,10] nonlinear dimension reduction,[11–14] and agglomerative clustering.[15] With advances in deep learning, deep networks are now also applied to unsupervised clustering.[16,17] For visualization, techniques such as t-distributed stochastic neighbor embedding (t-SNE)[18] and uniform manifold approximation and projection (UMAP)[19] effectively simplify complex data to reveal class structures and relationships.

Unsupervised clustering and visualization are driving innovations in cutting-edge scientific research and clinical practice. For example, in RNA sequencing (RNA-seq) analysis, these methods have successfully characterized both cellular heterogeneity and dynamic transcriptional states within complex biological systems.[20–22] Such an analysis requires handling high-dimensional data, identifying rare cell populations, and mapping

© 2025 The Author(s). Published by Elsevier Inc.
This is an open access article under the CC BY license (http://creativecommons.org/licenses/by/4.0/).

gradual transitions between cell states. In clinical settings, these approaches have proven instrumental in identifying and visualizing disease subtypes, particularly distinct pathophysiological mechanisms in diabetes.[23] In physics, unsupervised learning techniques are applied to analyze data from high-energy particle experiments and to study large-scale structure formation in the universe.[24]

Traditional algorithms struggle with challenges in modern applications, which demand high accuracy despite involving high-dimensional, complex data structures. For instance, high dimensionality can lead to spurious correlations and make distance metrics less meaningful; class heterogeneity in size, distribution, and density complicates the identification of true structures; and the presence of noise and sparsity, especially prevalent in biological data, can obscure true patterns and relationships. Moreover, no single model performs well across all domains. Methods optimized for one field often exhibit suboptimal performance in others, thus necessitating tailored adaptations and innovations in clustering and visualization techniques. Each field has distinct characteristics and requirements, which further complicates the development of universal unsupervised learning approaches.

Although multidisciplinary datasets have varied structures and patterns, unsupervised clustering commonly relies on two fundamental assumptions:

(1) nearby points are more likely to belong to the same class, which we call local consistency;
(2) local consistency can be propagated globally to establish overall connectivity within classes.

We consider this the *first principle* for unsupervised clustering. It establishes a mechanism that works regardless of data representation, distribution, structure, or application domain. Local consistency shows a positive correlation between spatial proximity and class membership. It measures the class consistency between neighbors. Global propagation requires a reliable mechanism to connect local consistencies. However, implementing the first principle is a difficult task since it depends on two key factors: precision in estimating class-consistent neighbors and robustness in propagating the estimated class consistency. In practice, the first principle has been largely overlooked in the literature.

Applying this first principle, this paper aims to achieve a global understanding of class patterns through local extraction for data clustering and visualization. We focus on efficiently identifying local class consistency and propagating it globally. Starting with raw data without any class information, the local consistency is typically weak, requiring carefully chosen small neighborhoods where neighbors belong to consistent classes. Global propagation and its enhancement are essential for accurate global understanding, yet achieving this remains challenging.

We address local extraction with three techniques: estimating class-consistent neighbors, quantifying local consistency, and enhancing this quantification to build a credibility graph for propagation. We implement global propagation through the credibility graph using an adaptive cutting technique. The adaptive cutting successively improves the self-learned class consistency when this extraction-propagation procedure is repeated.

Based on these techniques, we propose a method called GULE (global understanding via local extraction) to implement the first principle. Initially, the GULE algorithm performs local extraction and global propagation on the raw data, yielding a low-dimensional projection of the data points in which points are more closely grouped with their class-consistent neighbors. In other words, class-consistent neighborhoods are extended and their connections are tighter. We then repeat this process on the projected data to further refine class consistency. The credibility graphs generated at each stage differ in structure, which poses challenges for conventional spectral methods. GULE's adaptive graph cutting addresses this by dynamically aligning graph connection strengths with the cutting strategy. This reinforces intra-class connections and enhances the efficiency of global propagation.

GULE finally produces low-dimensional points that project the raw data, with dimensions matching the number of classes. We prove theoretically that these projected points accurately reveal the ground-truth classes of the raw data. As the final step of GULE, we propose a customized clustering method for these projected points. It solves the problem of completely positive factorization (CPF)[25] and is therefore referred to as CPF clustering. CPF clustering achieves superior robustness and accuracy compared with traditional clustering methods that rely on assumptions of cluster shape or density. The projected points can improve visualization by clearly distinguishing different classes. We apply t-SNE to a coupled dataset that combines raw data points and projected points. This combined approach highlights category information from the original data and preserves each class's topology structure in the visualization.

METHODS

The GULE algorithm consists of three modules (Figure 1A):

(1) A two-layer self-learning network that reveals class structures in unlabeled data by: extracting local class consistency, propagating it globally through a credibility graph with adaptive graph cutting, and iteratively enhancing the learned consistency.
(2) A clustering method (CPF clustering) specially tailored to the projected points.
(3) A modified visualization that combines projected points with raw data to preserve topological structure.

Module A: Two-layer self-learning network

GULE's first task is to efficiently extract the weak local class consistency from raw data as accurately as possible. Its second task is to globally propagate the extracted class information. The former establishes a graph for propagation, while the latter requires an intelligent approach that matches the graph structure for efficient propagation. We adopt a two-layer network to strengthen the self-learning results.

A1: Local extraction

The local extraction process comprises two steps: estimating the class-consistent neighborhoods and quantifying the estimated consistency using a credibility function.

Using only a suitable distance measure $d(\cdot,\cdot)$ and the number of classes K, we select a small neighborhood set

CellPress
OPEN ACCESS

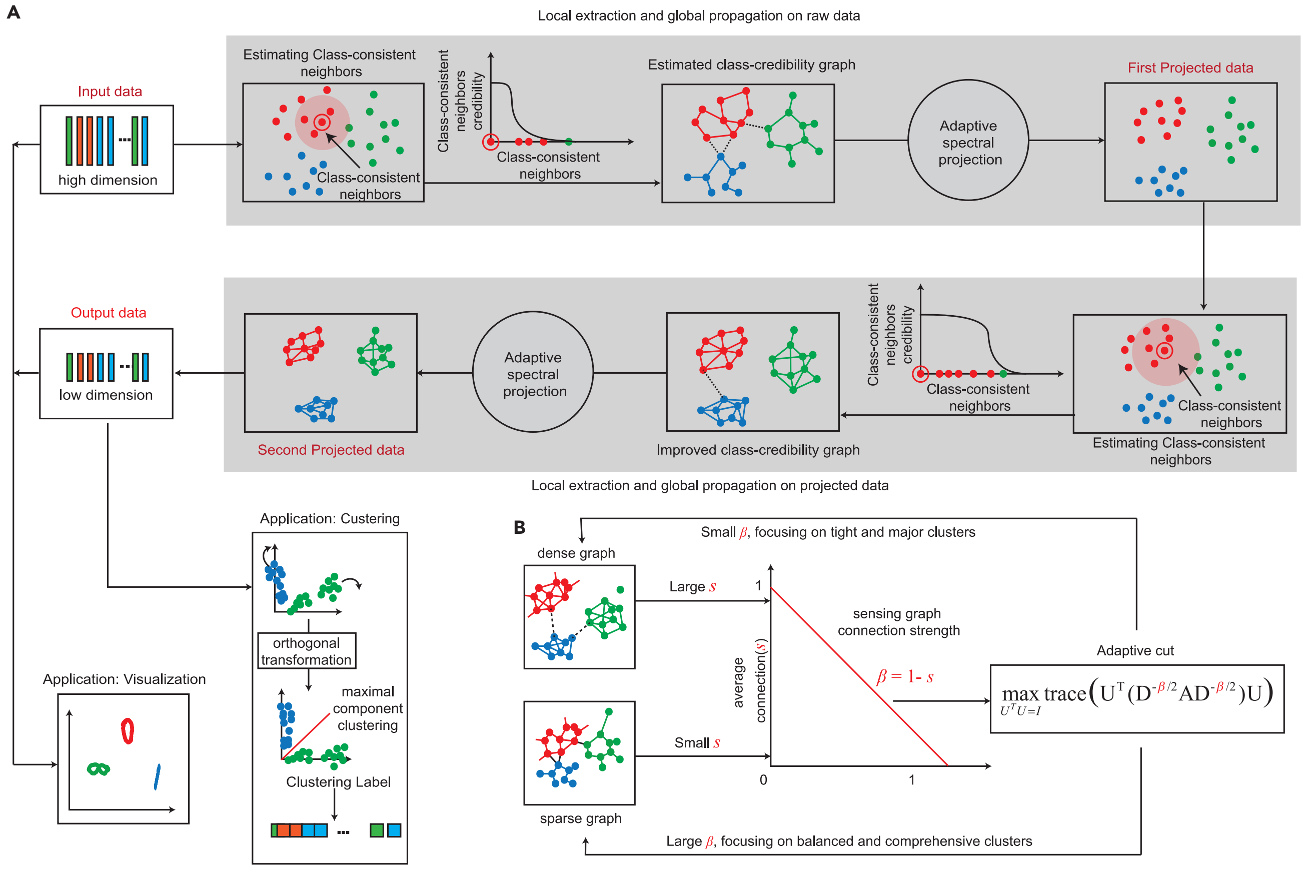

Figure 1. An overview of GULE

(A) Starting with raw data, GULE first estimates class-consistent neighbors, and calculates the local consistency. It then constructs a credibility graph and propagates the consistency using an adaptive graph cutting (Acut) technique, which yields a low-dimensional projection. GULE repeats this procedure on the projected data, ultimately producing a projection with dimensionality equal to the number of classes. GULE then offers two applications: CPF clustering via orthogonal transformation, and a visualization integrating both raw and projected data.

(B) Illustration of the Acut mechanism. When processing dense graphs with large average connection s, Acut employs smaller β values to focus on tight and major clusters, maximizing clustering accuracy. When processing sparse graphs, Acut utilizes larger β values to focus on balanced and comprehensive cluster structures, ensuring important data relationships are not overlooked.

$$N(x_i) = \{x_j : d(x_i, x_j) < \omega_i\}, \omega_i = d(x_i, x_{i_{k_c+1}}), \qquad \text{(Equation 1)}$$

for each point x_i in the raw dataset, taking the k_c nearest neighbors (including x_i itself) as class-consistent neighbors of x_i and the $(k_c + 1)$-st nearest neighbor $x_{i_{k_c+1}}$ class-inconsistent to x_i. The size k_c of $N(x_i)$ is a conservatively set integer:

$$k_c = k_0 + \lfloor \log_2(n / K) \rfloor, \qquad \text{(Equation 2)}$$

where k_0 is a small base number for avoiding an over-small neighbor set. We uniformly set $k_0 = 5$ across all datasets in our experiments. GULE is robust to k_0; for instance, it performs stably when k_0 ranges from 1 to 10 (Table S7).

In the second layer, operating on the nonlinearly projected points $\{y_i\}$, we measure spatial proximity using the cosine distance

$$d(y_i, y_j) = \sqrt{1 - \frac{\langle y_i, y_j \rangle}{\|y_i\|_2 \|y_j\|_2}}. \qquad \text{(Equation 3)}$$

For each point y_i, we define its estimated class-consistent neighborhood as

$$N(y_i) = \{y_j : d(y_i, y_j) < \omega_i\} = \{y_{i_1}, \ldots, y_{i_{k_i}}\}, \qquad \text{(Equation 4)}$$

where $\{y_{i_j}\}$ are the neighbors of y_i sorted by increasing distance and $\omega_i = d(y_i, y_{i_{k_i+1}})$ denotes the distance to the first excluded neighbor. Because of the global propagation of local consistency, y_i contains more class-consistent neighbors. The gathered neighbors have smaller distance gaps between them than with other points. To determine the number of neighbors k_i, we set

$$k_i = \min \left\{ \underset{j \geq k_c}{\arg\max} \left(d\left(y_i, y_{i_{j+1}}\right) - d\left(y_i, y_{i_j}\right), \left\lfloor \frac{n}{K} \right\rfloor \right\} . \qquad \text{(Equation 5)}$$

where $\lfloor \frac{n}{K} \rfloor$ is the average size of classes for restricting the size of each neighbor set.

An estimated neighbor set $N(x_i)$ may include points from different classes. Quantifying the class consistency of estimated neighbors is crucial to mitigate misestimation and ensure accurate propagation of class information.

Inspired by the relationship between a confidence interval and the standard deviation of a normal random variable, we treat an estimated neighbor set as a confidence set for class-consistent neighbors. Accordingly, we quantify the class consistency in $N(x_i) = \{x_j : d(x_j, x_i) < \omega_i\}$ with the credibility function

$$c_{ij} = \exp\left(- \frac{d^2(x_i, x_j)}{2(\omega_{ij}/\alpha)^2} \right), x_j \in N(x_i), \quad \text{(Equation 6)}$$

where $\omega_{ij} = \max\{\omega_i, \omega_j\}$ for $x_j \in N(x_i)$ or $x_i \in N(x_j)$. The parameter α tunes the confidence level of the neighbors' class consistency: A larger α credits credible consistency to fewer neighbors near the center, while a smaller α accepts the consistency of more neighbors. A similar formula also works for the projected points $\{y_i\}$.

We employ a relatively large α for the raw data points $\{x_i\}$ to conservatively estimate the credibility of initial neighbors. In our experiments, we set the parameter α as a constant

$$\alpha = \begin{cases} 9, & \frac{n}{K} \le 100; \\ 6, & \frac{n}{K} > 100, \end{cases} \quad \text{(Equation 7)}$$

which roughly depends on the average size of classes. For the projected points $\{y_i\}$, each set $N(y_i)$ of estimated neighbors contains more neighbors with improved class consistency. A smaller α is more reliable for further filtering the neighbors near the neighborhood boundary. We simply set $\alpha = 2$.

We enhance the previously defined credibility function (Equation 6) using a sigmoid transformation commonly employed in deep neural networks. Specifically, we modify the function c_{ij} to $\tilde{c}_{ij} = \text{sigm}(c_{ij})$. For simplicity, let $\gamma_{ij} = \frac{d(x_i, x_j)}{\omega_{ij}}$ (or $\gamma_{ij} = \frac{d(y_i, y_j)}{\omega_{ij}}$) denote the normalized distance between the raw data points x_i and x_j (or the projected points y_i and y_j). The credibility function c_{ij} can be represented as $c_{ij} = \exp\left(- \frac{(\alpha\gamma_{ij})^2}{2} \right)$. Hence, the enhanced credibility is defined by

$$\tilde{c}_{ij} = \frac{2c(\gamma_{ij}; \alpha)}{1 + c(\gamma_{ij}; \alpha)} = \frac{2}{1 + \exp\left((\alpha\gamma_{ij})^2 / 2 \right)}. \quad \text{(Equation 8)}$$

This transformation increases the credibility of neighbors near the center point in each neighborhood.

A2: Global propagation via credibility graph and adaptive graph cutting

To propagate local class consistency across the entire dataset, we construct an undirected credibility graph $G = (V, E)$ using the enhanced credibility function:

(1) Each vertex $v_i \in V$ represents a data point—either a raw point x_i or a projected point y_i.
(2) The edge between vertices v_i and v_j is weighted by

$$a_{ij} = \begin{cases} \frac{2}{1 + \exp\left((\alpha\gamma_{ij})^2 / 2 \right)}, & \text{if } \gamma_{ij} \le 1; \\ 0 & \text{otherwise,} \end{cases} \quad \text{(Equation 9)}$$

where $\gamma_{ij} = \frac{d(x_i, x_j)}{\omega_{ij}}$ for raw data or $\gamma_{ij} = \frac{d(y_i, y_j)}{\omega_{ij}}$ for projected points.

The two credibility graphs from $\{x_i\}$ or $\{y_i\}$ have different adjacency matrix structures due to their different neighborhood sizes and connection strengths. The classical spectral projection methods based on normalized cutting and ratio association for graph cutting[1] are not uniformly suitable for these different graphs.

In each layer, we use an adaptive strategy to cut the credibility graph A, which partitions the graph vertices into K clusters $\mathcal{C} = \{C_1, \ldots, C_K\}$ optimally (Figure 1B). The adaptive graph cutting (Acut) is modeled as the maximization problem

$$\max_{\mathcal{C}} \left\{ \text{Acut}(\mathcal{C}; \beta) = \sum_k \frac{\sum_{i,j \in C_k} a_{ij}}{\sum_{t \in C_k} d_t^\beta} \right\}, \quad \text{(Equation 10)}$$

where $d_i = \sum_j a_{ij}$ is the degree of vertex v_i, and $\beta = 1 - s$ is a constant that is adaptively set, depending on the average connection strength

$$s = \frac{1}{n} \sum_{k=1}^{K} \sum_{i \in C_k} \frac{\sum_{j \in C_k, j \ne i} a_{ij}}{|C_k| - 1}. \quad \text{(Equation 11)}$$

Clearly, $0 < s \le 1$. Here, $\frac{1}{|C_k| - 1} \sum_{j \in C_k, j \ne i} a_{ij}$ is the average connection strength of x_i to its class members. It can be estimated by $s_i = \frac{1}{|N_i| - 1} \sum_{j \ne i} a_{ij}$, where N_i is the neighborhood index set of y_i. Hence, we use

$$s \approx \frac{1}{n} \sum_i s_i = \frac{1}{n} \sum_i \frac{\sum_{j \ne i} a_{ij}}{|N_i| - 1}. \quad \text{(Equation 12)}$$

The graph cutting model Acut is tightly related to the traditional methods normalized cut (Ncut) and ratio association (RatioAssoc). Ncut minimizes $\sum_k \frac{\sum_{i \in C_k, j \notin C_k} a_{ij}}{\sum_{t \in C_k} d_t}$, the normalized connections between classes, and RatioAssoc maximizes $\sum_k \sum_{i \in C_k} d_i$, the connections within the classes. Acut adaptively optimizes both, as

$$\text{Acut} = \sum_k \left(\frac{\sum_{i \in C_k} d_i}{\sum_{t \in C_k} d_t^\beta} - \frac{\sum_{i \in C_k, j \in \bar{C}_k} a_{ij}}{\sum_{t \in C_k} d_t^\beta} \right) \quad \text{(Equation 13)}$$

with an adaptively chosen constant $\beta = 1 - s$. That is, it maximizes rescaled connections within the classes and minimizes rescaled connections between the classes. In the special case where s is small as in the first layer for the raw data with small neighbor sets, Acut approximates Ncut. When interior contacts are strong ($s \approx 1$), Acut is similar to RatioAssoc.

It is difficult to solve the discrete Acut model (Equation 10). To simplify the solution process, we relax it to the continuous model

$$\max_{U^T U = I} \text{trace}\left(U^T \left(D^{-\beta/2} A D^{-\beta/2} \right) U \right) \quad \text{(Equation 14)}$$

CellPress
OPEN ACCESS

The detailed derivation is provided in theoretical analysis, continuous relaxation of the discrete Acut. The continuous model (Equation 14) is equivalent to an eigenvalue problem whose solution is given by the eigenvectors $U = [u_1, \ldots, u_K]$ of $D^{-\beta/2}AD^{-\beta/2}$ corresponding to its K largest eigenvalues.

Module B: CPF clustering

The conventional spectral clustering approach is based on k-means to group projected data points. However, k-means has two major drawbacks. First, it assumes spherical clusters, which is rarely true for the row vectors of U in GULE. Second, it is highly sensitive to the initial selection of centroids, often yielding inconsistent results.

The dominant eigenvector matrix U has special structures, which help to retrieve the ground truth classes with high precision. We present a theoretical analysis that treats the credibility graph G as a perturbation of an ideal graph G_0. From G_0, we can directly retrieve ground truth classes through its dominant eigenvector matrix $U_0 = (u_{ik}^{(0)})$, using the simple labeling

$$\ell^*(x_i) = \arg\max_k u_{ik}^{(0)}. \qquad \text{(Equation 15)}$$

Our analysis gives a perturbation bound of G to G_0 (theoretical analysis, approximate block-diagonal structure of matrix G), showing that the perturbation error $E = G - G_0$ is small in norm if the local class consistency is propagated well. Using the perturbation theory of subspaces,[26] we further show the perturbation bound of U to the ideal U_0 within a suitable orthogonal transformation Q (theoretical analysis, nonnegative transformation of the eigenvectors of G).

The last task of the GULE algorithm for clustering is to determine the orthogonal transformation Q that guides U to the ideal but unknown U_0. That is, $UQ \approx U_0$. Since U_0 consists of the Perron vectors of the diagonal blocks of G_0, $UQ \approx U_0$ means that $UU^T \approx U_0U_0^T$. In other words, UU^T approximately has a CPF $U_0U_0^T$. It indicates that the ideal U_0 can be obtained by solving the approximate CPF of UU^T. We adopt the EPM algorithm proposed in Zhang and Li[25] to solve the CPF problem $A = BB^T$ with nonnegative factor B, given a completely positive matrix A. Here, the EPM iteratively solves the minimization problem

$$\min_{Q \in \mathbb{R}^{K\times K}} \frac{1}{4}\left\|QQ^T - I\right\|_F^2 + \frac{K}{n}\left\|(UQ)_-\right\|_F^2, \qquad \text{(Equation 16)}$$

given a low-rank factor UU^T of the CP matrix, where $(\cdot)_-$ refers to the negative part of a matrix.

As soon as the eigenvector matrix U is approximately transformed to a nonnegative matrix $\tilde{U} = UQ$ as U_0, the true class labels $\{\ell^*(x_i)\}$ can be estimated by

$$\tilde{\ell}(x_i) = \arg\max_k |\tilde{u}_{ik}|, i = 1, \ldots, n, \qquad \text{(Equation 17)}$$

as the ideal clustering approach (Equation 15). This method is referred to as CPF clustering. Theorem 1 in theoretical analysis, precision of CPF clustering guarantees that the CPF clustering has high precision.

Module C: Visualization

Visualization methods such as t-SNE[18] and UMAP[19] seek to find 2D or 3D points $\{z_i\}$ whose Euclidean distances match the original data distributions. While t-SNE uses asymmetric probabilities, UMAP employs a symmetric approach. Both methods iteratively optimize the solution starting with randomly selected points.

Our visualization method combines GULE projection with the above visualization techniques. Starting with the data points $\{x_i\}$, we first apply GULE to obtain the nonlinear low-dimensional projections $\{y_i\}$ of $\{x_i\}$. We then combine the normalized data distances with the cosine distances of the projected points using a convex combination. The GULE projection eliminates interclass connections through class consistency propagation. This increases inter-class distances while preserving intra-class distances. Hence, visualization with GULE projection highlights intra-class topological structures, making it more informative and discriminative.

Selection of distance metric

For textual data, the Spearman distance is particularly effective at extracting semantic information.[27] Hence, we consistently use it for all textual datasets. For non-textual data, no suitable distance metrics have been mentioned in existing literature. A straightforward—though inelegant—approach for non-textual data is to evaluate multiple distance metrics against the true class labels and select the best-performing one. However, since ground-truth labels are typically unavailable in real applications, we define a consensus score between two candidate distance metrics. Specifically, for any two metrics d and d', we define the consensus score as

$$c(d, d') = \mathrm{NMI}(\ell(d), \ell(d')),$$

where $\ell(d)$ and $\ell(d')$ are the clustering labels produced by GULE using metrics d and d', respectively, and NMI denotes normalized mutual information. We then compute the average consensus score for each metric:

$$\mathrm{Consensus}(d) = \frac{1}{|\mathcal{D}| - 1}\sum_{d' \neq d} c(d, d'),$$

and select the distance metric with the highest average score.

This selection method almost always identifies the optimal metric for non-textual datasets. Although the chosen metric may occasionally differ from the absolute best, the resulting clustering performance is nearly identical. We compare the selection rule and the optimal metric in Table S10. To demonstrate the efficiency of GULE, the results reported in this paper are based on the best-performing distance metric, which is consistent with the setting used for the comparison algorithms.

Comparison of GULE with traditional clustering methods

GULE fundamentally differs from traditional graph-based clustering methods in several ways: motivation (the first principle), technology (class-credibility graph, adaptive graph cutting, CPF clustering), universality (unrestricted data structures), and effectiveness (higher clustering accuracy (ACC) and topological-preserving visualization). Implementing the principle of achieving global understanding through local extraction and global propagation, GULE works with raw data first and then improves the initial results by continuously working with the projected data.

GULE does not make any assumptions about the data structures. It extracts local class consistency dynamically through adaptive neighborhood estimation and credibility quantification, then globally propagates this information via credibility graphs with adaptive spectral projection. In contrast, traditional graph-based methods were typically designed under special data structure assumptions, such as class-gathering of raw data points for k-means-like methods or subspace sampling for subspace-based methods. Based on these assumptions, traditional methods apply k-means clustering in different ways. Some apply it directly to raw data for class-gathered data. Others apply it to low-dimensional projected points obtained through various approaches: spectral methods (self-tuning spectral clustering [STSC][28] and sparse self-tuning spectral clustering [S-STSC][29]), penalized spectral methods (self-constrained spectral clustering [SCSC][30]), or normalized graph cutting (Ncut) with neighborhood Gaussian graphs. Some use modified approaches such as downsampling graphs (landmark-based spectral clustering [LSC][31]) or self-represented coefficient vectors sampled from subspaces (efficient dense subspace clustering [EDSC][32] and elastic net subspace clustering [EnSC][33]).

GULE can achieve high ACC via two-layer projections, focusing on class-consistent neighborhood estimation and the class-credibility graph. In addition to the credibility graph, the adaptive graph cutting method and the stable CPF clustering method also play an irreplaceable role in improving clustering effectiveness. Traditional algorithms cannot improve their results when repeated. This limitation stems from several factors: rough graph constructions, un-adaptable graph cutting on improved graphs, unstable k-means clustering for spectral projection algorithms, or irreversible merging procedures in graph degree linkage (GDL) and graph average linkage (GAL).[15]

RESULTS

GULE integrates multiple special techniques to extract class information, including estimating class-consistent neighbors, quantifying the consistency and strengthening reliable consistency, propagating the local consistency through the credibility graph with the adaptive graph cutting, and performing tailored CPF clustering for the iteratively enhanced projected points. These techniques significantly increase its ability to extract true class information. Since it focuses only on local relations, rather than data features, GULE has wide applications, not limited by data representations. On variant real-world datasets, GULE outperforms state-of-the-art clustering algorithms, including traditional methods and recently developed deep learning approaches for unsupervised clustering. GULE demonstrates superior ACC, competitive computational efficiency, improved extraction of class topological structures, and works well across diverse datasets.

Benchmark datasets and clustering algorithms

The experimental evaluation encompassed 22 diverse datasets, both classical and contemporary. These include object recognition, handwritten digit classification, facial analysis, fashion item categorization, text classification, time series analysis, satellite remote sensing, RNA-seq, and human activity recognition. A comprehensive breakdown of each dataset's characteristics, including application domain, sample size, feature dimensionality, number of classes, and class-size distribution, is provided in Note S2 and Tables S1 and S2.

Our comparative analysis covered two major categories of clustering algorithms: traditional approaches and deep-learning-based methods. The traditional algorithms encompass various approaches: spectral-clustering-related techniques (e.g., STSC,[28] S-STSC,[29] LSC,[31] and SCSC[30]), subspace-clustering methods (e.g., scalable EnSC[33] and EDSC[32]), graph-based clustering algorithms (e.g., GDL and GAL[15]), and the classical k-means algorithm.[34] The deep learning-based clustering techniques combine the representational power of deep neural networks with the strengths of traditional clustering methods, simultaneously optimizing feature extraction and clustering processes in an end-to-end manner. These approaches incorporate various innovative ideas, such as adversarial learning, subspace clustering, and graph convolutional networks, to enhance clustering performance and robustness.

We implemented all algorithms using the authors' recommended parameter settings and default configurations. See Note S1 for a brief description of the compared algorithms.

GULE has a wide applicability

Although existing clustering algorithms often excel in specific domains, they typically struggle with data from different fields exhibiting diverse representations, structures, and complexities. In contrast, GULE maintains its effectiveness, because it focuses on class consistency, rather than data features. This decreases the dependence on feature representation.

To demonstrate GULE's wide applicability, we evaluated GULE on 18 real-world datasets and three 2D synthetic datasets. The synthetic datasets were chosen to illustrate complicated data structures in various distributions, densities, relative locations of different classes, such as "compounded," "rounded," or "entangled" (as shown in the first column of Figure 2A, with a different color for each class).

GULE also remains robust even with complex class distributions. As shown in the second column of Figure 2A, the clusters extracted by GULE closely match the ground-truth classes. In contrast, the other traditional clustering algorithms displayed in the right columns in Figure 2A always failed to detect true classes, except GDL on the entangled set. The k-means and STSC favor separately gathered clusters such as spherical or ball-shaped clusters, while EnSC and EDSC can handle data approximately sampled from subspaces. The clustering results of these algorithms were likely based on spatial segmentation, completely ignoring the classes' different densities or distributions. The performances of S-STSC and SCSC were very similar, and so were those of LSC and GAL. These four algorithms partially focused on connectivity between points but neglected the density differences between classes. GDL favored neighborhood connectivity within classes but was also affected by different class densities.

GULE's effectiveness on complicated synthetic datasets suggests it is widely applicable to real-world datasets. GULE consistently performs very well on the 18 real-world datasets from various fields, as evaluated by the three widely adopted metrics: ACC, NMI, and adjusted rand index. The ACC results are presented in Figure 2B. Across these 18 real-world datasets,

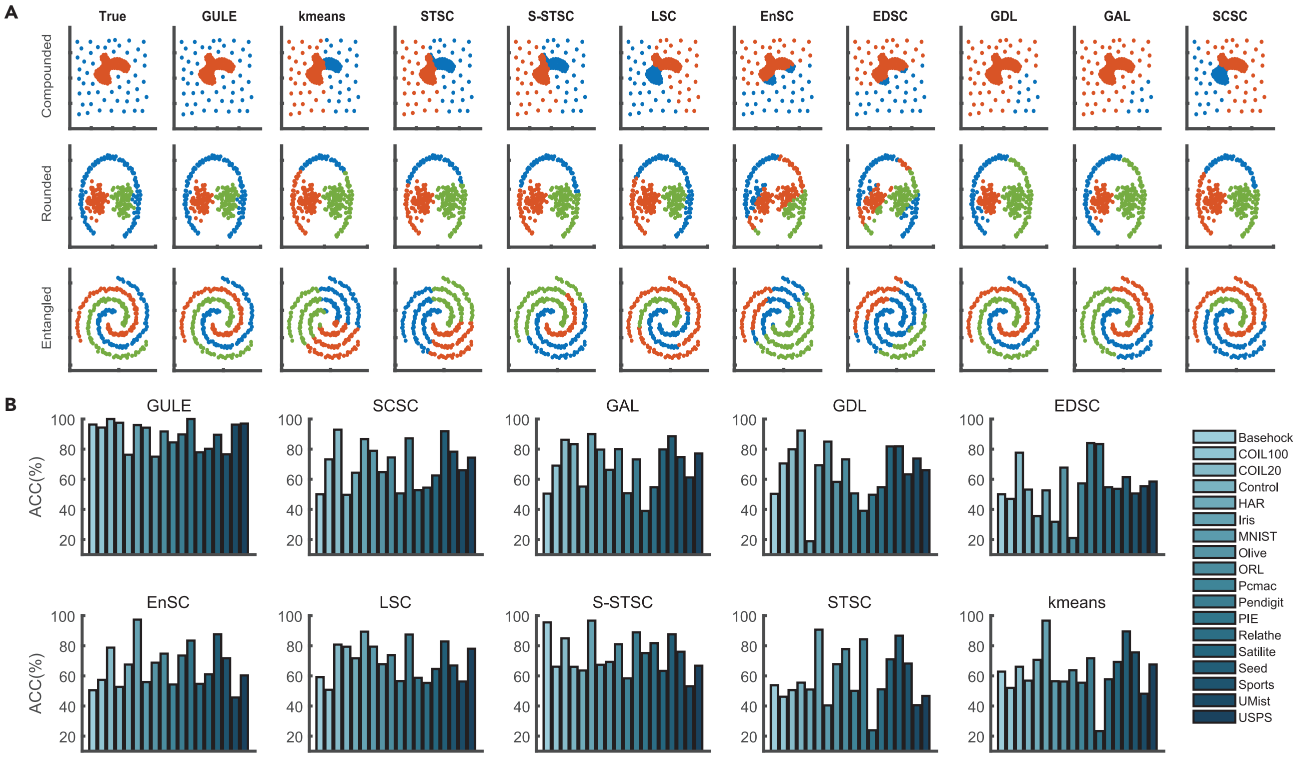

Figure 2. Comparison of GULE and state-of-the-art clustering methods on synthetic and real-world datasets

(A) Ground-truth class labels are shown in the first column of each 2D dataset, with different colors indicating different classes. The remaining columns display the clustering results obtained by GULE and nine benchmark algorithms, highlighting differences in class boundary identification and structural consistency.

(B) Barplots of clustering accuracy (ACC) distributions across 18 real-world datasets for each of the evaluated algorithms. GULE consistently achieves higher accuracy and lower variability, indicating robustness across heterogeneous data types.

GULE obtains a high average ACC of 89.6%, a small standard deviation of 8%. This performance is significantly better than that of the second-best method, S-STSC, which achieves an average ACC of 74.5% with a relatively larger standard deviation of 12%. This clearly demonstrates GULE's superiority, adaptability, and effectiveness across a wide range of data types. More detailed results for all evaluation metrics are provided in Tables S3–S5.

GULE outperforms traditional and deep learning clustering methods

To quantify GULE's improvements, we rank all algorithms according to their ACC values in Table 1. In most of the tested datasets, GULE significantly outperforms other algorithms: compared with the best of other algorithms, the relative improvement

$$\left(\frac{\text{GULE}}{\text{Best of Others}} - 1\right) \times 100\%$$

achieved by GULE on ACC is substantial. The second column in Table 1 lists the relative improvements in percentages. GULE achieves relative improvements of 20%–30% in five datasets, 10%–16% in three datasets, and 5.6% and 7.6% in two other datasets. For the remaining four datasets, GULE's ACC is slightly lower than the best-performing method, with relative decreases from 1.3%–2.6%.

GULE is also superior when benchmarked against leading deep-learning networks. Table S6 summarizes the performance of 24 deep learning algorithms for unsupervised clustering in ACC, and compares them with GULE. These deep learning algorithms may excel in specific scenarios but do not achieve GULE's uniformly high performance across diverse datasets.

GULE's role in exploring cellular functions and types

Traditional approaches for identifying biological functional regions have relied on diverse methodologies, including animal studies, neuroimaging, and clinical observations. Single-cell/nucleus RNA-seq has enabled high-resolution detection of functional regions through data analysis at the individual nucleus scale. However, this task requires unsupervised clustering methods with exceptional accuracy. GULE is thus well-suited to discovering different types and functions of cells.

In this section, we compare GULE with five classical clustering methods on three distinct RNA-seq datasets:

(1) GTEx brain dataset[35] covering the functional regions of the human brain. It contains 2,642 brain cells isolated from 8 different functional regions (cortex, basal ganglia, cerebellum, amygdala, hippocampus, hypothalamus, spinal cord, and substantia nigra) for cognitive processing, motor control, movement coordination, emotional processing,

Table 1. Performance comparison of GULE and competing clustering algorithms across multiple datasets

Data	Relative improvement (%)	Rank 1 Method	ACC (%)	Rank 2 Method	ACC (%)	Rank 3 Method	ACC (%)	Rank 4 Method	ACC (%)	Rank 5 Method	ACC (%)	Rank 6 Method	ACC (%)	Rank 7 Method	ACC (%)
Basehock	0.8	GULE	96.3	S-STSC	95.5	k-means	62.8	LSC	59.2	STSC	53.8	EnSC	50.5	GAL	50.5
COIL100	28.8	GULE	94.3	SCSC	73.2	GDL	70.5	GAL	69.1	S-STSC	66.1	EnSC	57.3	k-means	52.0
COIL20	7.6	GULE	100.0	SCSC	92.9	GAL	86.2	S-STSC	84.9	LSC	80.8	GDL	79.9	EnSC	78.8
Compounded	24.6	GULE	100.0	k-means	80.3	GDL	72.5	EnSC	71.8	LSC	66.9	EDSC	66.9	S-STSC	62.0
Control	5.6	GULE	97.5	GDL	92.3	GAL	83.3	LSC	79.3	S-STSC	66.0	k-means	56.8	STSC	55.5
Entangled	0.0	GULE	100.0	GDL	100.0	SCSC	76.6	S-STSC	70.8	LSC	58.0	GAL	51.9	STSC	43.3
HAR	6.4	GULE	76.3	LSC	71.7	k-means	70.6	EnSC	67.5	SCSC	64.3	S-STSC	63.6	GAL	55.2
Iris	−1.3	EnSC	97.3	k-means	96.7	S-STSC	96.7	GULE	96.0	STSC	90.7	GAL	90.0	LSC	89.3
MNIST	10.9	GULE	94.3	GDL	85.0	GAL	79.7	LSC	79.4	SCSC	78.9	S-STSC	67.3	k-means	56.5
Olive	8.3	GULE	75.0	S-STSC	69.3	EnSC	68.8	STSC	67.8	LSC	67.8	EDSC	67.8	GAL	66.3
ORL	13.3	GULE	91.8	S-STSC	81.0	GAL	80.0	STSC	77.8	EnSC	74.8	SCSC	74.5	LSC	73.8
Pcmac	44.8	GULE	84.5	S-STSC	58.4	EDSC	57.3	LSC	56.5	k-means	55.4	EnSC	54.3	GAL	50.7
Pendigit	1.0	GULE	89.8	S-STSC	88.9	LSC	87.5	SCSC	87.2	STSC	84.3	EDSC	84.0	EnSC	73.5
PIE	19.8	GULE	100.0	EnSC	83.4	EDSC	83.4	S-STSC	75.1	LSC	58.7	SCSC	52.8	GDL	49.7
Relathe	−4.6	S-STSC	81.7	GULE	77.9	k-means	57.7	LSC	55.3	EDSC	54.7	GDL	54.7	GAL	54.7
Rounded	12.5	GULE	99.3	LSC	88.3	GAL	82.3	S-STSC	81.0	GDL	80.7	STSC	78.7	SCSC	77.7
Satellite	−2.0	GDL	81.8	GULE	80.2	GAL	79.8	STSC	71.0	k-means	69.2	LSC	64.6	S-STSC	63.3
Seed	−3.6	SCSC	91.9	k-means	89.5	GULE	89.5	GAL	88.6	S-STSC	87.6	EnSC	87.6	STSC	86.7
Sports	−2.2	SCSC	78.3	GULE	76.6	S-STSC	76.0	k-means	75.6	GAL	74.7	EnSC	71.7	STSC	68.2
UMist	30.5	GULE	96.3	GDL	73.8	SCSC	66.0	GAL	61.2	LSC	56.3	EDSC	55.4	S-STSC	53.1
USPS	24.3	GULE	97.0	LSC	78.0	GAL	77.2	SCSC	74.4	k-means	67.6	S-STSC	66.7	GDL	66.0

memory formation, homeostasis regulation, signal transmission, and dopaminergic modulation, respectively.

(2) Zeisel dataset[36] covering the transcriptional diversities of cells from mouse brain. It contains 3,005 single cells from 7 different transcriptional types (astrocytes-ependymal, endothelial-mural, interneurons, microglia, oligodendrocytes, pyramidal CA1, and pyramidal SS).

(3) Darmanis dataset[37] covering the developmental states of adult and fetal human brains. It consists of 466 single cells, categorized into 9 types (oligodendrocyte precursor cells, astrocytes, endothelial cells, fetal quiescent and replicating cells, hybrid cells, microglia, neurons, and oligodendrocytes).

Detailed description of the datasets is given in Note S5.

Clustering performances of the six algorithms GULE, LSC, EnSC, EDSC, GAL, and GDL on the GTEx dataset are visualized through confusion matrices as shown in Figure 3A. These matrices display the distribution of cells from identical functional regions (rows) across clustered groups (columns). A diagonally dominant matrix indicates optimal functional separation, with off-diagonal elements representing misclassified cells. Similar confusion matrices of these algorithms on the Zeisel dataset and Darmanis dataset are given in Figures S1 and S2. These confusion matrices show that GULE performs significantly better than the other algorithms on these three RNA-seq datasets:

(1) GULE can completely detect the eight functionalities from the GTEx dataset—its confusion matrix is highly diagonally dominant with negligible misclassified cells. However, each of the other algorithms can only detect partial functionalities: LSC misclassifies almost half of the cells from amygdala, hippocampus, and spinal cord regions. GDL also misclassifies half of the cells from the hippocampus region. EnSC has 26% misdetection of cells in the basal ganglia region. EDSC loses many cells from basal ganglia, hippocampus, and spinal cord regions. GAL loses all cells in the four regions: amygdala, hippocampus, spinal cord, and substantia nigra.

(2) GULE's performance on the Zeisel dataset is much better than that of the other algorithms. It achieves an average recall of 94% and precision of 92% across all seven cell types, with particularly strong performance in identifying interneurons and oligodendrocytes where other methods struggle. In practice, the other algorithms have at least one cell type completely missing (GAL and GDL misclassify two cell types and four types, respectively).

(3) For the more challenging Darmanis dataset with rare cell populations, GULE maintains robust performance (average recall of 89% and precision of 86%) despite the dataset's high cell type heterogeneity and limited sample size. The other five methods struggle with fetal cell distinction and neuron classification, and also completely miss at least one type.

To demonstrate in detail how GULE performs better than the other algorithms, we list the recall and precision of the algorithms for eight functional regions in the GTEx brain dataset in Table 2. Similar comparisons on the Zeisel and Darmanis datasets are also given in Figures S1 and S2. This consistent performance

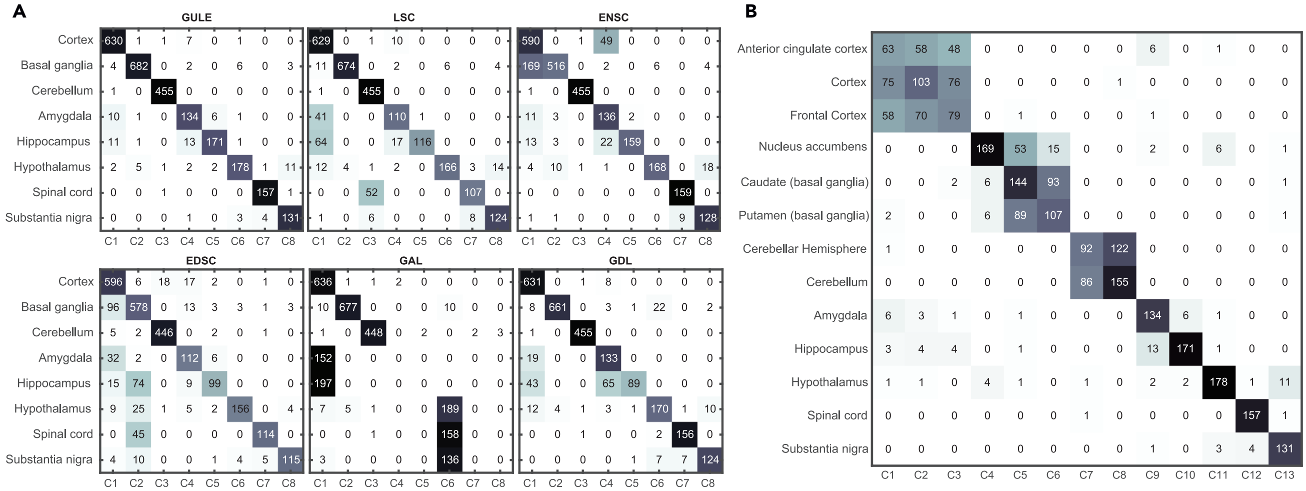

Figure 3. Clustering performance and anatomical resolution of brain region cell distributions

(A) Confusion matrices comparing the clustering results of six algorithms (GULE, LSC, EnSC, EDSC, GAL, and GDL) across eight functional brain regions. Each matrix reflects how cells from true brain regions (rows) are assigned to predicted clusters (columns), with stronger diagonal signals indicating higher region-specific accuracy.

(B) High-resolution confusion matrix from GULE clustering, showing detailed cell distribution across 13 anatomically distinct brain regions. The matrix reveals both within-region cell homogeneity (diagonal elements) and inter-region relationships (off-diagonal elements), capturing nuanced structure-function associations in the brain.

of GULE across species demonstrates GULE's robustness in handling heterogeneous single-cell RNA-seq (scRNA-seq) datasets.

The GTEx dataset was labeled in 13 groups to account for subdivisions, as the cortex region consists of the overlapped anterior cingulate cortex and frontal cortex, the basal ganglia contains the nucleus accumbens, caudate, and putamen, and the cerebellum is associated with the cerebellar hemispheres. We also applied GULE to this dataset using 13 classes with the confusion matrix shown in Figure 3B. As expected, cells from each of the first three regions could not be clearly separated, reflecting the inherent ambiguities in the subdivisions. Interestingly, GULE identified the nucleus accumbens as an independent cluster (C4). This aligns with its known functional specialization of serving as a crucial interface between the limbic and motor systems, as elaborated in Floresco,[38] where the nucleus accumbens acts as a translator while the caudate and putamen mainly control body movements. This indicates GULE's potential for revealing biological mechanisms. The results of the other five clustering algorithms were not as clear as GULE's.

GULE demonstrates competitive computational efficiency

GULE achieves exceptional performance at a competitive computational cost. In practice, compared with traditional algorithms on relatively large datasets, GULE without the visualization step is only slightly slower than LSC and S-STSC, and faster than others. LSC achieves faster processing through coarse downsampling, significantly reducing its effectiveness and stability, while S-STSC relies solely on a single spectral projection on a neighborhood Gaussian graph combined with k-means. Thus, GULE's slightly lower speed is acceptable as it employs double projections. Figure 4A illustrates the runtime of traditional algorithms in 18 datasets, sorted by increasing computational time.

Two main processes account for most of GULE's computational costs: constructing the credibility graph and computing the dominant eigenvectors of the credibility matrix for adaptive spectral projection. For the first layer, projection computation dominates due to the graph's sparsity from small neighborhoods. For the second layer, graph construction becomes more computationally intensive as neighborhoods expand. However, the projection cost decreases for this layer due to the approximate low-rank of the improved graph. The additional cost of CPF clustering is negligible. Figure 4B illustrates the distribution of GULE's computational costs for 3 datasets with 70,000 samples each. Using only CPUs, GULE efficiently performs clustering and yields competitive performance in less than 400 s.

GULE enhances data visualization by preserving topological structures

Real-world data in applications often contain intrinsic topological structures within classes. This phenomenon can be partially demonstrated using synthetic datasets (left column of Figure 5A): "compound" shows classes with varying density distributions, "rounded" features two central clusters and one curved cluster, and "entangled" consists of three classes with overlapping topological structures. Effective visualization methods must preserve and represent such inherent topological features.

GULE is primarily a data-preparation framework rather than a visualization algorithm. However, its superior class information extraction can enhance the performance of dimensionality reduction methods such as t-SNE[18] and UMAP[19] by combining the raw data with their GULE-projected points using these visualization techniques.

Table 2. Recall and precision of clustering algorithms across eight functional brain regions: CTX, BG, CB, AMG, HPC, HT, SC, and SN

Brain region	CTX	BG	CB	AMG	HPC	HT	SC	SN
Recall (%)								
GULE	98	98	100	88	87	88	99	94
LSC	98	97	100	72	59	82	67	89
EnSC	92	74	100	89	81	83	100	92
EDSC	93	83	98	74	50	77	72	83
GAL	99	97	98	0	0	94	0	0
GDL	99	95	100	88	45	84	98	89
Precision (%)								
GULE	96	99	99	84	96	94	97	90
LSC	83	99	88	78	99	97	91	87
EnSC	75	97	100	65	99	97	95	85
GAL	63	100	99	0	0	39	0	0
GDL	88	99	99	63	97	85	95	91

Cortex, CTX; basal ganglia, BG; cerebellum, CB; amygdala, AMG; hippocampus, HPC; hypothalamus, HT; spinal cord, SC; substantia nigra, SN.

Figure 5A shows t-SNE visualizations comparing raw data with the improved results after applying GULE's first- or second-layer projections. The comparison includes three synthetic datasets and three real-world datasets—COIL20, PIE, and MNIST. These topological structures are not well visualized by the classical t-SNE and UMAP on the raw data as shown in the top row of Figure 5A. Without coloring, classes and class topologies are difficult to distinguish. However, GULE accurately captures the topology of the synthetic datasets. It also clarifies the circular structure of COIL20, the linear patterns of PIE, and the uniform clustering characteristics of MNIST. The middle and bottom rows of Figure 5A show how topology preservation improves when raw data are combined with GULE's first- or second-layer projections. GULE successively improves class topology preservation as class separation increases after the first and second projections. For instance, GULE's first layer projection of the entangled data recovers the topology of one class, while preserving only the one-dimensional structure for the other two classes. The second layer further recovers the curvatures of these two classes, similar to how it recovered the first class. A similar improvement is observed in the real-world datasets PIE and MNIST. The topology recovery closely depends on the clustering precision. GULE's first projection achieved perfect clustering on the other three datasets. Therefore, it recovered topology equally well in both the first and second layers for these datasets. To further illustrate GULE's ability to preserve topology, we analyzed two similar car classes from the COIL20 dataset, which differed only in some subtle features (Figure 5B). Both classes exhibit circular topology due to their rotational image capture. Discriminating between these classes was particularly challenging, as it required high-resolution classification. Standard t-SNE visualization fails to differentiate these car classes, resulting in distorted topology, as shown in the left panel of Figure 5C. In contrast, GULE-based visualization effectively separates the classes while preserving their intrinsic circular topology. The middle and right panels of Figure 5C show the visualizations using the projections in the first and second layers, respectively. The two results are similar since GULE also provided perfect clustering in the first layer.

Theoretical analysis

The numerical analyses in the previous section have demonstrated GULE's ability to adapt to complex datasets and achieve high ACC. This outstanding performance can be attributed to the first principle underlying GULE and its efficient implementation. This section presents our theoretical contribution to the GULE framework:

1. (1) Continuous model of the discrete Acut, which relaxes the challenging discrete optimization to the simple computation of dominant eigenvectors of the rescaled credibility graph G, and aids theoretical analysis.
2. (2) Error analysis of the rescaled credibility graph deviating from an ideal graph whose dominant eigenvectors can retrieve the ground-truth classes. This analysis provides insight into the retrieval of ground-truth classes.
3. (3) Subspace perturbation, which links the eigenvectors of the rescaled credibility graph to the ideal but unknown eigenvectors of the ideal graph. The discovery of the CPF approximation of UU^T ensures that the ideal eigenvectors are computable via an orthogonal transformation that solves the CPF problem using our proposed EPM algorithm.[25]
4. (4) Precision estimation of the CPF clustering. The CPF clustering on the transformed eigenvectors of the rescaled graph is an approximation of the ideal clustering on the ideal eigenvectors of the ideal graph.

All proofs for this section can be found in Note S3.

Continuous relaxation of the discrete Acut

The discrete model (Equation 10) of Acut is difficult to solve. However, it can be relaxed to a continuous model to simplify the solution process. To this end, let $u_k = (u_{1k}, \dots, u_{nk})^T$ be the column vector with entries

$$u_{ik} = \begin{cases} \sqrt{\dfrac{d_i^\beta}{\sum_{t \in C_k} d_t^\beta}} & i \in C_k \\ 0 & i \in \overline{C}_k, \end{cases} \qquad \text{(Equation 18)}$$

where $U = [u_1, \dots, u_K]$ is orthonormal: $U^T U = I_K$. Since $u_{ik} d_i^{\beta/2} = \left(\sum_{t \in C_k} d_t^\beta\right)^{-1/2}$ for $i \in C_k$,

$$\sum_k \frac{\sum_{i,j \in C_k} a_{ij}}{\sum_{t \in C_k} d_t^\beta} = \sum_k \sum_{i,j \in C_k} u_{ik} d_i^{-\beta/2} a_{ij} d_j^{-\beta/2} u_{jk}$$
$$= \operatorname{trace}\left(U^T D^{-\beta/2} A D^{\beta/2} U\right).$$

Relaxing the discrete restriction in Equation 18 on $\{u_k\}$ while preserving their orthogonality and unit normalization, the discrete Acut (Equation 10) is transformed to its continuous version (Equation 14). As with classical spectral methods, the continuous version (Equation 14) is equivalent to the eigenvalue problem of computing K eigenvectors of $D^{-\beta/2} A D^{\beta/2}$ corresponding to K largest eigenvalues.

CellPress
OPEN ACCESS

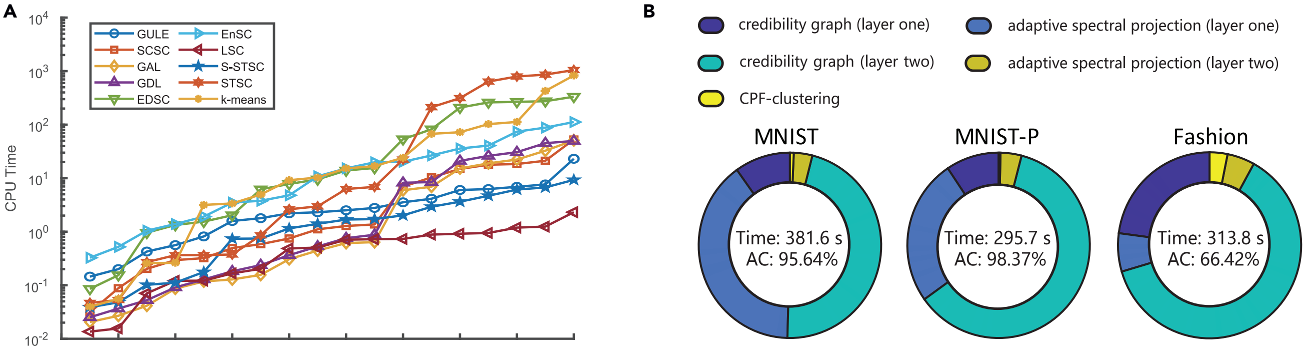

Figure 4. Computational efficiency of GULE compared to benchmark clustering algorithms

(A) Total runtime of each algorithm on the benchmark datasets, shown in ascending order to highlight differences in efficiency.

(B) Breakdown of GULE's runtime across its five computational phases—local consistency extraction, graph construction, adaptive cutting, spectral projection, and CPF clustering—on three large-scale datasets.

Approximate block-diagonal structure of matrix G

The rescaled matrix $G = D^{-\beta/2}AD^{-\beta/2}$ of the credibility matrix A is approximately block-diagonal, if we partition A into a $K \times K$ block-matrix according to the K ground-truth classes as

$$A = \begin{bmatrix} A_{11} & \cdots & A_{1K} \\ \vdots & \ddots & \vdots \\ A_{K1} & \cdots & A_{KK} \end{bmatrix}.$$

Because of the two-layer learning process that ensures class consistency, the off-diagonal blocks of A are relatively small in norm. In other words, A is approximately block-diagonal as

$$A_0 = \text{diag}(A_{11}, \ldots, A_{KK}).$$

The diagonal blocks $\{A_{kk}\}$ may not be connected; each can be further partitioned as a block-diagonal matrix of connected blocks. That is, without loss of generality, $A_{kk} = \text{diag}(A_1^{(k)}, \ldots, A_{p_k}^{(k)})$ with connected blocks $\{A_i^{(k)}\}$. The adaptive scaling in Acut strengthens the dominant sub-diagonal blocks in each A_{kk}. That is, partitioning the rescaled matrix $G = (G_{k\ell})$ as $A = (A_{k\ell})$, each G_{kk} has a dominant sub-diagonal block with stronger connections than others.

To show the approximate structure of G, let $r_i(A) = a_{i1} + \ldots + a_{in}$ and $r(A) = (r_1(A); \ldots; r_n(A))$, where the semicolon indicates a column link for simplicity, i.e.,

$$(r_1(A); \ldots; r_n(A)) = (r_1(A), \ldots, r_n(A))^T.$$

Then, $G_{k\ell} = D_k^{-\beta/2}A_{k\ell}D_\ell^{-\beta/2}$ with $D_k = \text{diag}(r(A_k))$ and $r(A_k) = (r_1(A_k); \ldots; r_{n_k}(A_k))$. The key idea is to take G as a perturbed matrix of a modified block-diagonal matrix G_0, rather than using the natural block-diagonal matrix $\text{diag}(G_{11}, \ldots, G_{KK})$ derived from G. That is,

$$G = G_0 + E, G_0 = \text{diag}(\overline{G}_{11}, \ldots, \overline{G}_{KK}), \overline{G}_{kk} = \overline{D}_k^{-\beta/2}A_{kk}\overline{D}_k^{-\beta/2},$$

with the diagonal matrix $\overline{D}_k = \text{diag}(r(A_{kk}))$. This is critical to the analysis. To bound the perturbation matrix E, let

$$\rho_k = \left(\frac{r_1(A_{kk}^c)}{r_1(A_{kk})}; \ldots; \frac{r_{n_k}(A_{kk}^c)}{r_{n_k}(A_{kk})}\right), \rho_k^{(\beta)} = \left(\frac{r_1(A_{kk}^c)}{r_1^\beta(A_{kk})}; \ldots; \frac{r_{n_k}(A_{kk}^c)}{r_{n_k}^\beta(A_{kk})}\right),$$

where $A_{kk}^c = [A_{k1}, \ldots, A_{k,k-1}, 0, A_{k,k+1}, \ldots, A_{kK}]$, the k-th row-block of A excluding the diagonal block A_{kk}. Note that $\rho_k^{(\beta)} = \rho_k$ if $\beta = 1$ and $\rho_k^{(\beta)} = (r_1(A_{kk}^c); \ldots; r_{n_k}(A_{kk}^c))$ if $\beta = 0$. Generally, $\rho_k^{(\beta)}$ and ρ_k are small if the class-consistent neighborhoods are estimated well. The following Lemma gives an upper bound of the perturbation matrix E in terms of the norm of $\rho^{(\beta)} = (\rho_1^{(\beta)}; \ldots; \rho_K^{(\beta)})$.

Lemma 1: The error matrix $E = G - G_0$ is bounded as $\|E\|_2 \le (1+\beta)\|\rho^{(\beta)}\|_\infty$.

Nonnegative transformation of the eigenvectors of G

Each $\overline{G}_{kk}$ is nonnegative and symmetric. By the Perron-Frobenius theorem,[39] it has a nonnegative eigenvector $\overline{u}_k$ corresponding to its largest eigenvalue $\lambda_1(\overline{G}_{kk})$. Furthermore, the eigenvector is guaranteed to be positive when $\overline{G}_{kk}$ is connected. Hence, the block diagonal G_0 has K sparse and nonnegative eigenvectors $\{u_k^{(0)}\}$, taking $\overline{u}_k$ as the nonzero piece of $u_k^{(0)}$ with the index set as that of A_{kk} in A. These vectors span the invariant subspace of G_0, that is, the invariant subspace takes $U_0 = [u_1^{(0)}, \ldots, u_K^{(0)}]$ as a basis matrix.

The invariant subspace perturbation theorem in Golub et al.[26] indicates that, as a perturbed matrix of G_0 shown by Lemma 1, G has an invariant subspace that approximates the subspace of G_0. Applying the subspace perturbation theorem, we have the following.

Lemma 2: Let $U = [u_1, \ldots, u_K]$ be the K eigenvectors of G corresponding to the K largest eigenvalues. Then there is an orthogonal matrix Q such that

$$\|U_0 - UQ\|_F \le \frac{2\sqrt{2}\sqrt{K}\|E\|_2}{\lambda_K(G_0) - \lambda_{K+1}(G_0)}. \quad \text{(Equation 19)}$$

It should be pointed out that the perturbation of U to U_0 depends on not only the graph error E, but also the gap between the K-th and $(K+1)$-th eigenvalues of G_0.

Precision of CPF clustering

Let $UQ = (\tilde{u}_{ik})$ and

$$\mathcal{I}_k = \left\{ i \in C_k^* : |\tilde{u}_{ik}| \le \max_{\ell \ne k} |\tilde{u}_{i\ell}| \right\}.$$

Only those vertices with indices in $\mathcal{I} = \mathcal{I}_1 \cup \ldots \cup \mathcal{I}_K$ may be mislabeled by Equation 17. Let $\delta_K(G_0)$ be the gap between the

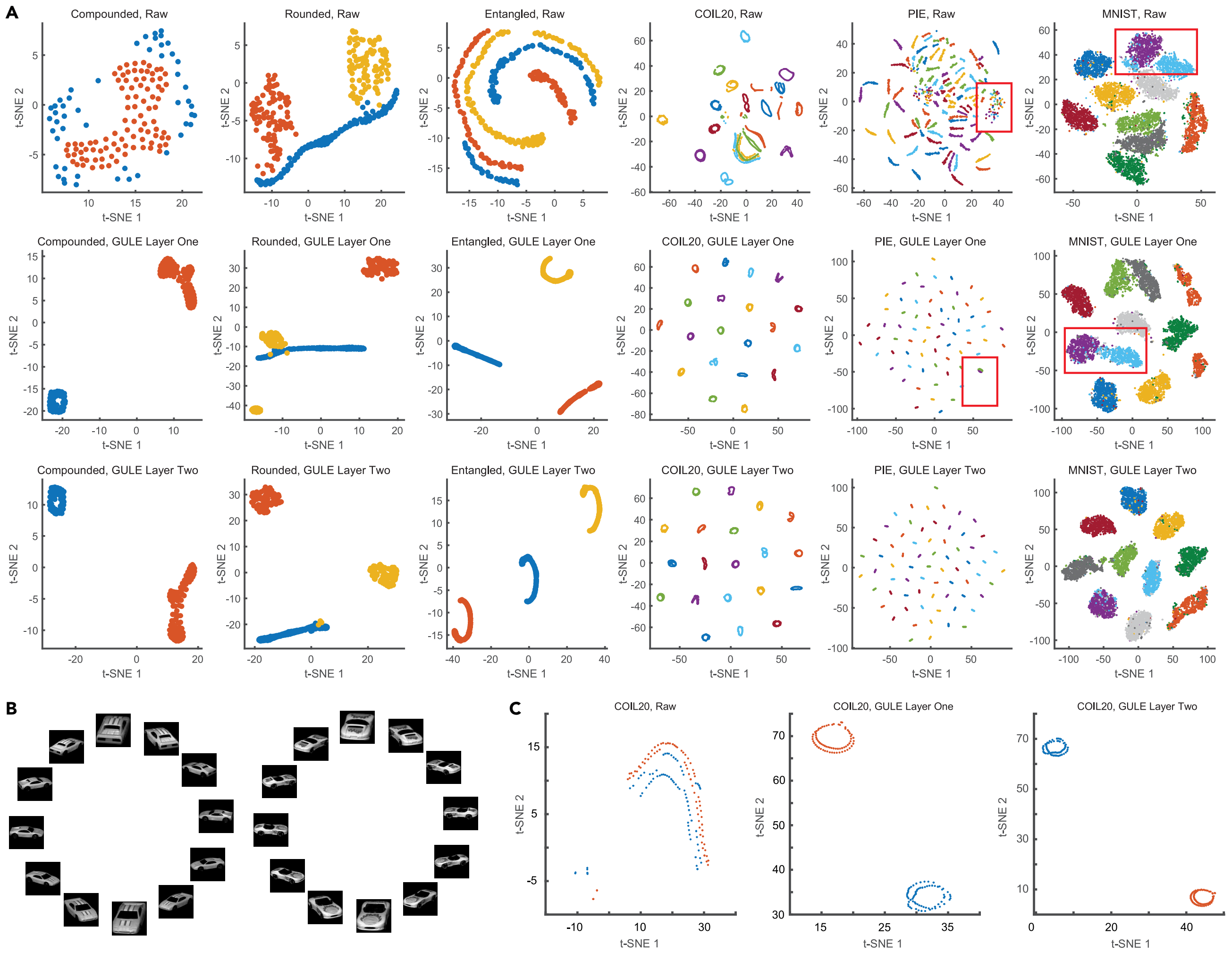

Figure 5. Topological enhancement of data visualization through GULE projections

(A) t-SNE visualizations of six datasets comparing three representations: raw data (top row), data after the first-layer projection by GULE (middle row), and data after the second-layer projection (bottom row).

(B) Representative image sequences from two car object classes in the COIL20 dataset, captured under a 360° rotation. The smooth pose transition within each class forms a continuous visual manifold.

(C) t-SNE plots of the two car classes shown in (B), based on raw data (left), first-layer GULE projection (middle), and second-layer GULE projection (right).

K-th and $(K+1)$-th eigenvalues of G_0, and let $\varepsilon = \beta\|\rho\|_F + \tau^{\beta/2}\|\rho^{(\beta)}\|_F$, with $\tau = \max_{ij}\frac{r_i(A)}{r_j(A)}$. By definition,

$$\sum_k \sum_{i\in\mathcal{I}_k} \tilde{u}_{ik}^2 \le \|U_0 - UQ\|_F^2 \le \frac{8\varepsilon^2}{\delta_K^2(G_0)}.$$

Hence, sorting the entries $\{\tilde{u}_{ik}^2\}$ as $\{\eta_p^2\}$ in ascending order, there are at most p points that are mislabeled, where

$$p = \mathrm{argmax}\left\{p : \eta_1^2 + \cdots + \eta_p^2 \le \frac{8\varepsilon^2}{\delta_K^2(G_0)}\right\}. \qquad \text{(Equation 20)}$$

The analysis is in the following theorem:

Theorem 1: If each $\overline{G}_{kk}$ is connected and $\delta_K(G_0) > 0$, the CPF clustering (Equation 17) has at most p points misclustered, with p defined in Equation 20.

Interpretability

Although traditional clustering approaches offer analogous methods, they cannot adequately replace GULE's specialized modules for complex unsupervised clustering. In practice, replacing any of these specialized techniques with conventional methods would significantly reduce GULE's effectiveness.

Figure 6 shows the results of ablation experiments that illustrate the unique contributions of each GULE component in synthetic or real-world datasets.

Significance of class-consistent credibility graph

Structurally, the symmetric credibility function (Equation 6) is similar to the self-tuning similarity defined in Zelnik-Manor and Perona[28] as $s_{ij} = \exp\left(-\frac{d^2(x_i,x_j)}{\sigma_i'\sigma_j'}\right)$ for $i \ne j$ if $d(x_i,x_j) < \max\{\sigma_i', \sigma_j'\}$, and $s_{ij} = 0$ for $i = j$, where $\sigma_i' = d(x_i, x_{i_{k'}})$ defines the distance of x_i from its k'-th (default $k' = 7$) closest neighbor. However,

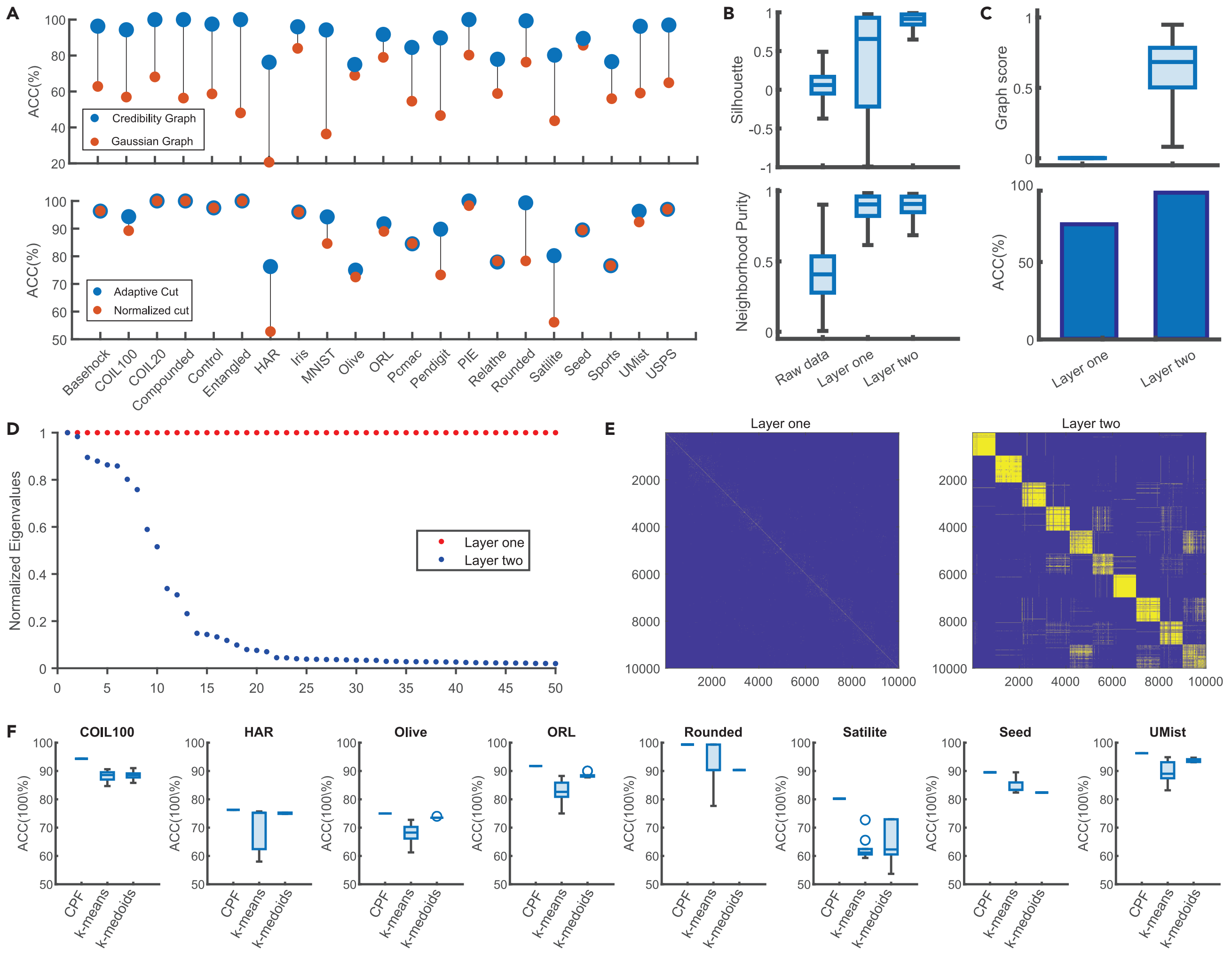

Figure 6. Component-wise analysis of the GULE framework and its performance contributions

(A) Comparison of clustering accuracy when using the credibility graph versus a traditional neighborhood Gaussian graph (top), and using the adaptive graph cut versus the classical normalized cut for spectral projection (bottom), across benchmark datasets.

(B) Boxplots showing silhouette coefficients (top) and neighborhood purity (bottom) for the raw MNIST data, and for projections obtained from the first and second layers of GULE.

(C) Improvements in graph score (top) and clustering accuracy (bottom) on MNIST across GULE's two projection layers.

(D) Eigenvalue spectra of the credibility graph at the two projection stages for MNIST. All eigenvalues are normalized by the largest eigenvalue to facilitate comparison.

(E) Sparsity patterns of the credibility graph at both stages for MNIST. Yellow regions denote non-zero values, while blue regions indicate zero entries, illustrating the evolution of graph structure and sparsification during learning.

(F) Clustering performance comparison between CPF (GULE's clustering module) and two classical methods (k-means and k-medoids) applied on GULE-projected data from 8 real-world datasets. Results are averaged over 20 repetitions.

there are two key differences between the credibility function and the self-tuning similarity:

(1) The credibility function focuses on class-consistent neighbors and the strength of connections among such neighbors. The size of estimated class-consistent neighborhoods may vary, particularly in different data environments. Connections within classes are necessary for highly accurate clustering. In contrast, the self-tuning similarity typically considers geometric neighborhoods of fixed size, ignoring the variance of data environments and the important consistency and connection.

(2) The credibility function contains a parameter α to provide additional control over the decay rate for neighbors near the neighborhood boundary, which facilitates differentiation between classes, although α is typically set to an approximate value. The credibility function also enhances the connectivity of neighbors close to the centers via the sigmoid transformation. In contrast, the self-tuning similarity employs a fixed decay rate for between-neighborhood similarities, and does not adequately adjust for class-specific local structures.

The top row of Figure 6A illustrates GULE's advantages (blue dots) over the Gaussian graph (red dots). GULE achieves an average ACC 30% higher than the Gaussian graph across all datasets, whereas the Gaussian graph achieves an ACC below 60% in over half of the instances examined. This underscores the critical role of the credibility graph in GULE's superior performance.

Effect of α and β

A high ACC of spectral methods requires a graph to have strong connections within classes and very weak connections between classes. The parameters α and β affect the constructed credibility graphs in GULE.

The role of α is to adjust the class-consistent credibility of neighbors near the neighborhood boundary. Increasing α can reduce the risk of mistakenly including these neighbors and increase the credibility of neighbors close to the center of the neighborhood. Conversely, a relatively small α allows for as many neighbors as possible to be considered credible neighbors belonging to the same class as the center point.

We suggest a relatively large α as in Equation 7 for the raw data in the first layer of GULE, resulting in a sparse credibility graph in which each point has strong connections to its close neighbors. The dependence of GULE on different values of α is shown in Table S8. In the second layer, the projected points have improved class distributions, and each point has larger and more credible neighbors. We use a relatively small $\alpha = 2$, which yields a credibility graph with strong connections within the classes. The settings may not be optimal but work well in numerical experiments on varied real-world datasets or complicated synthetic datasets reported in this paper.

The effect of β is a bit complicated. Basically, a suitable β helps to determine a good graph cutting for clustering. There are two goals for graph cutting: maximize the connections within classes and minimize the connections between classes. The adaptive graph cutting Acut maximizes the weighted connections within classes with a weight for each class that can tune the two goals. For a large $\beta \approx 1$, the maximization is approximately equivalent to minimizing the normalized connections between classes, ignoring the connections within classes. Meanwhile, a small $\beta \approx 0$ focuses on maximizing the connections within classes. Therefore, β serves as a parameter that tunes the relative importance of these two goals. If the graph is not ideal: block diagonal with K connected diagonal blocks, one may have to balance the two goals to achieve a clustering result as accurate as possible. The dependence of GULE on different values of β is shown in Table S9.

Effectiveness of adaptive spectral projection

In the two layers of GULE, the credibility graphs (or matrices) have significantly different structures. For raw data, the credibility matrix is sparse and of higher rank, whereas for projected points it is denser and of lower rank (Figures 6D and 6E). The classical spectral projection using Ncut is suited to raw data because the credibility matrix lacks clear block structures. However, Ncut does not improve clustering since its normalization strategy treats groups of different sizes fairly, leading it to mistakenly couple blocks from different classes when the graph has weakly connected or disconnected fragments in the same class.

Acut can adaptively match the varying structures of credibility matrices. To quantify and compare the effects of Acut and Ncut, we conducted an ablation experiment, replacing Acut with Ncut in GULE, as shown in the bottom part of Figure 6A. In every case, Acut's performance was either superior to or on par with Ncut's. This superiority was particularly pronounced in datasets such as HAR, MNIST, Pendigit, Rounded, and Satellite.

Progressive learning of class consistency

Starting with raw data lacking additional class information, GULE progressively refines its understanding of the data's class structure through incremental improvements, instead of aiming for a single significant enhancement.

This ability can be illustrated by improvements in the two layers of GULE on MNIST, evaluated using external indices: silhouette coefficient[40] (SC), neighborhood purity (NP), graph score (GS), and ACC. These definitions can be found in Note S4.

Figure 6B shows boxplots of SC and NP for raw data and projected points from the first and second layers of progressive learning. The upward shift in these boxplots highlights the effectiveness of progressive learning: local neighborhoods become more class consistent, clusters grow more cohesive internally, and they separate better from one another. Similar progressive improvements can be observed in the credibility graphs measured by GS or ACC as shown in Figure 6C.

Efficiency of CPF clustering

The classical clustering algorithms k-means and k-medoids are commonly used in data analysis. These methods typically initialize with randomly chosen starting centers, which can lead to unstable results due to sensitivity to initial conditions. In contrast, CPF clustering demonstrates superior stability and consistency.

Figure 6F illustrates the comparative performance of these clustering algorithms through ACC boxplots. The experiment was carried out on eight real-world datasets, with each algorithm run 20 times using random initialization. It should be noted that, on other datasets, there is no difference in the performance of the three algorithms. These results show that CPF clustering not only achieves higher accuracy, but also exhibits significantly lower variability in its results compared with k-means and k-medoids.

DISCUSSION

GULE has three significant advantages. First, it integrates class-consistent credibility graphs and adaptive spectral projection for complex unsupervised learning. This is particularly advantageous in datasets with complex or overlapping class structures, as the credibility graph captures subtle relationships that other methods miss. Second, the adaptively progressive learning aspect sets GULE apart from traditional clustering methods that often rely on static graph environments and single-pass optimizations. Third, GULE demonstrates broad applicability to diverse data across research fields, robustness in computation, efficient processing, and highly accurate capability to extract global class information. Its ability to cluster scRNA-seq data enables detailed analysis of cellular interactions and tissue functions, and broader applications in fields requiring high-precision data analysis.

However, despite its strong performance across various datasets, GULE has certain limitations at present:

(1) GULE could be developed to integrate partial meaningful labels, especially for complex data with weak connections

CellPress
OPEN ACCESS

to class-consistent neighbors. The main difficulty is detecting points with weak connections. Addressing this issue is also valuable when labeling is costly in applications.

(2) GULE currently sets α as almost a constant empirically and sets β in an adaptive way depending on the estimated connection strength. These settings work well in our experiments. Nevertheless, taking both α and β as tunable parameters and finding an intelligent solver could efficiently increase the ACC on more complicated data.

(3) GULE, like many clustering algorithms, requires prior specification of the cluster number. While this is a common constraint in clustering methods, we recognize the value of developing techniques that can automatically determine the optimal number of clusters. For future work, we aim to extend GULE to include robust mechanisms for estimating and validating the appropriate number of clusters when class structures are unknown *a priori*.

RESOURCE AVAILABILITY

Lead contact

Requests for further information and resources should be directed to and will be fulfilled by the lead contact, Bingjie Li (bjlistat@nus.edu.sg).

Materials availability

This study did not generate new materials.

Data and code availability

All original code supporting this study is publicly available. The full implementation of GULE can be accessed and downloaded from GitHub at https://github.com/bjli1992/GULE. A permanent version has also been archived on Zenodo at https://doi.org/10.5281/zenodo.15201785.[41]

ACKNOWLEDGMENTS

This work was supported in part by NSFC project 11971430 and the Major Scientific Research Project of Zhejiang Lab (no. 2019KB0AB01).

AUTHOR CONTRIBUTIONS

Conceptualization, Z.Z.; methodology, Z.Z. and B.L.; investigation, Z.Z. and B.L.; writing – original draft, Z.Z. and B.L.; writing – review & editing, Z.Z. and B.L.; funding acquisition, Z.Z.; resources, B.L.; supervision, Z.Z.

DECLARATION OF INTERESTS

The authors declare no competing interests.

SUPPLEMENTAL INFORMATION

Supplemental information can be found online at https://doi.org/10.1016/j.patter.2025.101266.

Received: November 16, 2024
Revised: March 23, 2025
Accepted: April 28, 2025
Published: May 19, 2025

REFERENCES

1. Shi, J., and Malik, J. (2000). Normalized cuts and image segmentation. IEEE Trans. Pattern Anal. Mach. Intell. *22*, 888–905. https://doi.org/10.1109/34.868688.
2. Wang, S., and Siskind, J.M. (2003). Image segmentation with ratio cut. IEEE Trans. Pattern Anal. Mach. Intell. *25*, 675–690. https://doi.org/10.1109/TPAMI.2003.1201819.
3. Leibe, B., Leonardis, A., and Schiele, B. (2008). Robust object detection with interleaved categorization and segmentation. Int. J. Comput. Vis. *77*, 259–289. https://doi.org/10.1007/s11263-007-0095-3.
4. Bach, F.R., and Jordan, M.I. (2006). Learning spectral clustering, with application to speech separation. J. Mach. Learn. Res. *7*, 1963–2001. http://jmlr.org/papers/v7/bach06b.html.
5. Warren Liao, T. (2005). Clustering of time series data—a survey. Pattern Recogn. *38*, 1857–1874. https://doi.org/10.1016/j.patcog.2005.01.025.
6. Lloyd, S. (1982). Least squares quantization in pcm. IEEE Trans. Inf. Theor. *28*, 129–137. https://doi.org/10.1109/TIT.1982.1056489.
7. Ng, A., Jordan, M., and Weiss, Y. (2001). On spectral clustering: Analysis and an algorithm. Adv. Neural Inf. Process. Syst. *14*. https://proceedings.neurips.cc/paper_files/paper/2001/file/801272ee79cfde7fa5960571fee36b9b-Paper.pdf.
8. Hagen, L., and Kahng, A. (1991). Fast spectral methods for ratio cut partitioning and clustering. In 1991 IEEE International Conference on Computer-Aided Design Digest of Technical Papers (IEEE Computer Society), pp. 10–11. https://doi.org/10.1109/ICCAD.1991.185177.
9. Wold, S., Esbensen, K., and Geladi, P. (1987). Principal component analysis. Chemometr. Intell. Lab. Syst. *2*, 37–52. https://doi.org/10.1016/0098-3004(93)90090-R.
10. Lee, D.D., and Seung, H.S. (1999). Learning the parts of objects by non-negative matrix factorization. Nature *401*, 788–791. https://doi.org/10.1038/44565.
11. Roweis, S.T., and Saul, L.K. (2000). Nonlinear dimensionality reduction by locally linear embedding. Science *290*, 2323–2326. https://doi.org/10.1126/science.290.5500.2323.
12. Zhang, Z., and Zha, H. (2004). Principal manifolds and nonlinear dimensionality reduction via tangent space alignment. SIAM J. Sci. Comput. *26*, 313–338. https://doi.org/10.1137/S1064827502419154.
13. Tenenbaum, J.B., de Silva, V., and Langford, J.C. (2000). A global geometric framework for nonlinear dimensionality reduction. Science *290*, 2319–2323. https://doi.org/10.1126/science.290.5500.2319.
14. Zha, H., and Zhang, Z. (2009). Spectral properties of the alignment matrices in manifold learning. SIAM Rev. *51*, 545–566. https://doi.org/10.1137/060676829.
15. Zhang, W., Wang, X., Zhao, D., and Tang, X. (2012). Graph degree linkage: Agglomerative clustering on a directed graph. In Computer Vision–ECCV 2012: 12th European Conference on Computer Vision, Florence, Italy, October 7-13, 2012, Proceedings, Part I 12, A. Fitzgibbon, S. Lazebnik, P. Perona, Y. Sato, and C. Schmid, eds. (Springer), pp. 428–441. https://doi.org/10.1007/978-3-642-33718-5_31.
16. Yang, J., Parikh, D., and Batra, D. (2016). Joint unsupervised learning of deep representations and image clusters. In Proceedings of the IEEE Conference on Computer Vision and Pattern Recognition (IEEE), pp. 5147–5156. https://openaccess.thecvf.com/content_cvpr_2016/papers/Yang_Joint_Unsupervised_Learning_CVPR_2016_paper.pdf.
17. Zhou, P., Hou, Y., and Feng, J. (2018). Deep adversarial subspace clustering. In Proceedings of the IEEE Conference on Computer Vision and Pattern Recognition (IEEE), pp. 1596–1604. https://openaccess.thecvf.com/content_cvpr_2018/papers/Zhou_Deep_Adversarial_Subspace_CVPR_2018_paper.pdf.
18. Van der Maaten, L., and Hinton, G. (2008). Visualizing data using t-SNE. J. Mach. Learn. Res. *9*, 2579–2605. https://jmlr.org/papers/v9/vandermaaten08a.html.
19. McInnes, L., Healy, J., and Melville, J. (2018). UMAP: Uniform manifold approximation and projection for dimension reduction. Preprint at arXiv. https://doi.org/10.48550/arXiv.1802.03426.
20. Kiselev, V.Y., Kirschner, K., Schaub, M.T., Andrews, T., Yiu, A., Chandra, T., Natarajan, K.N., Reik, W., Barahona, M., Green, A.R., and Hemberg, M.

(2017). SC3: consensus clustering of single-cell RNA-seq data. Nat. Methods *14*, 483–486. https://doi.org/10.1038/nmeth.4236.

21. Hao, Y., Hao, S., Andersen-Nissen, E., Mauck, W.M., Zheng, S., Butler, A., Lee, M.J., Wilk, A.J., Darby, C., Zager, M., et al. (2021). Integrated analysis of multimodal single-cell data. Cell *184*, 3573–3587.e29. https://doi.org/10.1016/j.cell.2021.04.048.
22. Yao, Z., Li, B., Lu, Y., and Yau, S.T. (2024). Single-cell analysis via manifold fitting: A framework for RNA clustering and beyond. Proc. Natl. Acad. Sci. USA *121*, e2400002121. https://doi.org/10.1073/pnas.2400002121.
23. Schön, M., Prystupa, K., Mori, T., Zaharia, O.P., Bódis, K., Bombrich, M., Möser, C., Yurchenko, I., Kupriyanova, Y., Strassburger, K., et al. (2024). Analysis of type 2 diabetes heterogeneity with a tree-like representation: insights from the prospective German Diabetes Study and the LURIC cohort. Lancet Diabetes Endocrinol. *12*, 119–131. https://doi.org/10.1016/S2213-8587(23)00329-7.
24. Blance, A., and Spannowsky, M. (2021). Unsupervised event classification with graphs on classical and photonic quantum computers. J. High Energy Phys. *2021*, 170. https://doi.org/10.1007/JHEP08(2021)170.
25. Zhang, Z., and Li, B. (2021). Exterior point method for completely positive factorization. Preprint at arXiv. https://doi.org/10.48550/arXiv.2102.08048.
26. Golub, G.H., and Van Loan, C.F. (2013). Matrix Computations (JHU Press).
27. Arsov, N., Dukovski, M., Evkoski, B., and Cvetkovski, S. (2019). A measure of similarity in textual data using spearman's rank correlation coefficient. Preprint at arXiv. https://doi.org/10.48550/arXiv.1911.11750.
28. Zelnik-Manor, L., and Perona, P. (2004). Self-tuning spectral clustering. Adv. Neural Inf. Process. Syst. *17*. https://proceedings.neurips.cc/paper_files/paper/2004/file/40173ea48d9567f1f393b20c855bb40b-Paper.pdf.
29. Kuang, D., Yun, S., and Park, H. (2015). SymNMF: nonnegative low-rank approximation of a similarity matrix for graph clustering. J. Glob. Optim. *62*, 545–574. https://doi.org/10.1007/s10898-014-0247-2.
30. Bai, L., Liang, J., and Zhao, Y. (2023). Self-constrained spectral clustering. IEEE Trans. Pattern Anal. Mach. Intell. *45*, 5126–5138. https://doi.org/10.1109/TPAMI.2022.3188160.
31. Cai, D., and Chen, X. (2015). Large scale spectral clustering via landmark-based sparse representation. IEEE Trans. Cybern. *45*, 1669–1680. https://doi.org/10.1109/TCYB.2014.2358564.
32. Ji, P., Salzmann, M., and Li, H. (2014). Efficient dense subspace clustering. In IEEE Winter Conference on Applications of Computer Vision (IEEE), pp. 461–468. https://doi.org/10.1109/WACV.2014.6836065.
33. You, C., Li, C.G., Robinson, D.P., and Vidal, R. (2016). Oracle based active set algorithm for scalable elastic net subspace clustering. In Proceedings of the IEEE Conference on Computer Vision and Pattern Recognition (IEEE), pp. 3928–3937. https://openaccess.thecvf.com/content_cvpr_2016/papers/You_Oracle_Based_Active_CVPR_2016_paper.pdf.
34. Arthur, D., and Vassilvitskii, S. (2007). K-means++: the advantages of careful seeding. In Proceedings of the Eighteenth Annual ACM-SIAM Symposium on Discrete Algorithms (Society for Industrial and Applied Mathematics), pp. 1027–1035.
35. GTEx Consortium (2020). The GTEx Consortium atlas of genetic regulatory effects across human tissues. Science *369*, 1318–1330. https://doi.org/10.1126/science.aaz1776.
36. Zeisel, A., Muñoz-Manchado, A.B., Codeluppi, S., Lönnerberg, P., La Manno, G., Juréus, A., Marques, S., Munguba, H., He, L., Betsholtz, C., et al. (2015). Cell types in the mouse cortex and hippocampus revealed by single-cell RNA-seq. Science *347*, 1138–1142. https://doi.org/10.1126/science.aaa1934.
37. Darmanis, S., Sloan, S.A., Zhang, Y., Enge, M., Caneda, C., Shuer, L.M., Hayden Gephart, M.G., Barres, B.A., and Quake, S.R. (2015). A survey of human brain transcriptome diversity at the single cell level. Proc. Natl. Acad. Sci. USA *112*, 7285–7290. https://doi.org/10.1073/pnas.1507125112.
38. Floresco, S.B. (2015). The nucleus accumbens: an interface between cognition, emotion, and action. Annu. Rev. Psychol. *66*, 25–52. https://doi.org/10.1146/annurev-psych-010213-115159.
39. Schur, J. (1905). Zur theorie der vertauschbaren matrizen. J. für die Reine Angewandte Math. (Crelle's J.) *1905*, 66–76. https://doi.org/10.1515/crll.1905.130.66.
40. Rousseeuw, P.J. (1987). Silhouettes: a graphical aid to the interpretation and validation of cluster analysis. J. Comput. Appl. Math. *20*, 53–65. https://doi.org/10.1016/0377-0427(87)90125-7.
41. Li, B., and Zhang, Z. (2025). Code for the article "global understanding via local extraction for data clustering and visualization". Zenodo. https://doi.org/10.5281/zenodo.15201786.

Patterns, Volume *6*

Supplemental information

Global understanding via local extraction for data clustering and visualization

Zhenyue Zhang and Bingjie Li

Note S1. Compared Algorithms

The compared algorithms can be divided into six categories: K-means clustering, low-rank approximation, spectral method, subspace learning, agglomerative clustering, and deep networks.

K-means clustering. Lloyd's approach[1] of classic K-means on data points is widely used. It converges to a local optimum generally. The algorithm K-means++[2] uses a heuristic to choose the centroid seeds. It improves the running time and the solution of Lloyd's algorithm successfully,

Spectral methods. We compare four spectral methods: self-tuning spectral clustering (STSC)[3], sparse self-tuning spectral clustering (S-STSC)[4], landmark-based spectral clustering (LSC)[5], and self-constrained spectral clustering (SCSC)[6]. The STSC and S-STSC construct a dense self-tuned Gaussian graph and a sparse self-tuned Gaussian graph, respectively. The LSC choose p landmark points $\{u_1, \cdots, u_p\}$ with $p \ll n$ at first. Then represent each data point x_j as a p-dimensional weight vector $w_i = (w_{1i}, \cdots, w_{pj})^T$ with the normalized Gaussian weights

$$w_{ij} = \frac{g_{ij}}{\sum_k g_{kj}}, \quad g_{ij} = \begin{cases} \exp(-\frac{\|u_i - x_j\|^2}{2\sigma^2}), & i \in I_i; \\ 0, & i \notin I_i, \end{cases}$$

where I_j is the index set of r landmark points nearest to x_j and $\sigma^2 = \frac{1}{n^2}\sum_{ij}\|x_i - x_j\|^2$ is the mean of all the squared pair-wise distances of the data points. The two parameters $r \leq p \ll n$ should be carefully turned for each data set. The p-dimensional points are further projected on the n_c-dimensional dominant subspace of the range space of $\{y_j\}$, and the clustering is obtained by K-means on the n_c-dimensional projected points $\{z_j\}$. Self-constrained spectral clustering (SCSC)[6] extends the traditional spectral clustering objective function with pairwise and label self-constrained terms. This approach simultaneously learns clustering results and constraints without prior information, effectively guiding the clustering process in unsupervised settings. An iterative method solves the resulting optimization problem, enabling the discovery of high-quality cluster structures in datasets.

Subspace learning. Assuming that the data points are approximately sampled from several subspaces, subspace learning aims to learn the latent subspaces by segmenting the data points into several classes corresponding to the subspaces. A self-expression approach is commonly adopted to linearly represent each point by other points that likely come from the same subspace. Hence, a similarity graph can be constructed by the representation weights. The Scalable Elastic Net Subspace Clustering (EnSC)[7] penalizes the coefficient vector in both the ℓ_1-norm and ℓ_2-norm in the self-expression, and the Efficient Dense Subspace Clustering (EDSC)[8] looks for the self-expression on a noise-free matrix of data points to decrease the interference of data noise.

Agglomerative clustering. The agglomerative clustering merges a pair of small clusters successively until all the small clusters are merged into K large ones. In[9], the initial clusters are chosen as the small k-NN neighborhoods with $k \leq 2$. A direct Gaussian graph of a bit larger neighborhoods is also used to define the linkage degrees between clusters and merge the pair of current clusters having the largest linkage to a larger one. This algorithm is named as GDL (graph degree linkage), or GAL (graph average linkage) that uses the symmetric form of the graph.

Deep networks. Twenty four algorithms based on deep networks for unsupervised learning are compared: Joint Unsupervised Learning (JULE)[10], Deep Subspace Clustering Networks (DSC)[11], Deep Adversarial Subspace Clustering (DASC)[12], Discriminatively boosted clustering (DBC)[13], Deep discriminative model (DDM)[14], Graph convolutional autoencoder using Laplacian smoothing and sharpening (GALA)[15], Not Too Deep Clustering model (N2D)[16], Spectral clustering via ensemble deep autoencoder learning (EDAE)[17], Deep Subspace Image Clustering Network with Self-expression and Self-supervision (DSCNSS)[18], self-attention deep subspace clustering (SADSC)[19], deep multi-view clustering approach based on the reconstructed self-expressive matrix (DCRSM)[20], Over-complete Deep Subspace Clustering Networks (ODSC)[21], Wasserstein embedding clustering (WEC)[22], deep clustering with contractive representation learning and focal loss (DCCF)[23], Deep Fusion Clustering Network (DFCN)[24], Efficient Deep Embedded Subspace Clustering (EDESC)[25], Deep Clustering by Multi-level Feature fusion (DCMF)[26], Deep Embedded Clustering (DEC)[27], local-to-global deep clustering method based on approximate uniform manifold (LGC-AUM)[28], deep fuzzy -means clustering (RD-FKC)[29], Discriminative Pseudo Supervision Clustering (DPSC)[30], Deep Gaussian Mixture Model (DeepGMM)[31], Deep Structural Contrastive Subspace Clustering (DSCSC)[32], and deep subspace clustering via dual adversarial generative networks (DSC-DAG)[33]. As known, algorithms based on deep networks need to turn a lot of parameters involved in the network construction for each data set and cost much for computation. Generally, these deep network algorithms can achieve higher clustering accuracy than the classical methods.

Note S2. Data Information

All the tested databases reported mentioned in this paper can be downloaded online. Table S1-S2 lists the sources of these databases, together with the distance metric used to show the efficiency of the GULE in this paper. The real-world data sets come from six categories. Brief descriptions of these databases are provided below.

- Basehock[34]. This is a text dataset consisting of 1993 instances, each with 4862 features. It has 2 classes, where the maximum class size is 999, and the minimum is 994.
- COIL-20 and COIL-100[35]. These datasets were created at Columbia University. COIL-20 contains 1440 grayscale images of 20 objects, while COIL-100 has 7200 images of 100 objects. They are widely used for object recognition and pose estimation tasks. The images were taken at pose intervals of 5 degrees, resulting in 72 images per object.
- Control[36]. This is a set of time series of length 60, synthetically generated by the process described in Alcock's work . It contains 600 examples of six control charts: normal, cyclic, increasing trend, decreasing trend, upward shift, and downward shift. Each chart accounts for 100 samples.
- Fashion-MNIST[37]. This is a dataset of 28x28 grayscale images, created as a drop-in replacement for the original MNIST dataset by Zalando Research . It contains 70,000 examples of ten fashion item categories: t-shirt/top, trouser, pullover, dress, coat, sandal, shirt, sneaker, bag, and ankle boot. Each category accounts for 7,000 samples, evenly split into 6,000 training and 1,000 test images.
- HAR (Human Activity Recognition)[38]. This dataset was collected from smartphone accelerometer and gyroscope readings. It contains measurements from 30 subjects performing six activities (walking, walking upstairs, walking downstairs, sitting, standing, laying).
- Iris[39]. One of the oldest and most famous datasets in pattern recognition, the Iris dataset was introduced by Ronald Fisher in 1936. This data set has 3 classes of 50 instances each. Each class refers to a type of iris plant, and each sample is represented by a 4D attribute including sepal length, sepal width, petal length, and petal width.
- MNIST[40]. Created by Yann LeCun and colleagues, MNIST (Modified National Institute of Standards and Technology database) consists of 70,000 handwritten digit images, split into 60,000 training images and 10,000 test images. In this experiment, we use both the whole set (MNIST70K), the testing set(MNIST), and the preprocessed data set (MNIST-PT).
- Olive Face (ORL)[41].This is a set of facial images from the Olivetti faces database, originally compiled by AT&T Laboratories Cambridge. It comprises 400 grayscale images of 40 distinct subjects, with each subject represented by 10 samples. These samples capture various facial expressions, lighting conditions, and facial details (e.g., with/without glasses). In our experiment, we utilize two versions of this dataset: Olive and ORL, which differ only in resolution. The Olive version consists of 64x64 pixel images, while the ORL version maintains the original 92x112 pixel resolution. Both variants provide a standardized format for facial recognition tasks and algorithm development, allowing for comparative analysis across different image resolutions.
- Pendigit[42]. This is a set of 10992 16-dimensional vectors. Each is a pen-based trace of 8 temporally sampled (x, y)-coordinates of a handwritten digit from one of 44 writers. As a set of handwritten digits, it also has 10 classes.
- Pcmac[34]. This is a text classification dataset with 1943 instances and 3289 features divided into 2 classes, derived from documents related to PCs and Macs.
- PIE[43]. This data set has 2856 face images of 68 persons in the small size 32×32. Each person has 42 facial images under different lighting and illumination conditions.
- Relathe[34]. Another text classification dataset with 1427 instances and 4322 features divided into 2 classes. It is used for evaluating text classification algorithms.
- Satellite[44]. This is a set of 6435 multi-spectral points of seven soil cover states: red soil, cotton crop, grey soil, damp grey soil, soil with vegetation stubble, mixture class, and very damp grey soil. Each point is a 36-dimensional vector, consisting of 4 spectral values of each pixel in a 3×3 region of a satellite image.
- Seeds[45]. This is a data set of 210 wheat seeds divided into 3 classes. Each sample contains 7 attributes: area, perimeter, compactness, kernel length, kernel width, asymmetry coefficient, and length of kernel groove.

- Sports[46]. This is a text dataset of sports news articles, collected from various online sources. It contains 1,000 examples of two sports categories. The articles are in English and vary in length, with an average of approximately 400 words per article.
- UMist[47]. This data set contains 565 images of 20 persons. Each person covers a range of poses from profile to frontal views and has various numbers of images.
- USPS[48]. This is a set of normalized gray-scale images of handwritten digits in size 16 × 16, automatically scanned from envelopes by the U.S. Postal Service. It contains 7291 training examples and 2007 test examples. We use the whole set, with a total of 9298 images.

Note S3. Mathematical Analysis of Graph Perturbations and Theorems

Assume that we are given a neighbor set N_i for each vertex v_i, and the neighbors of v_i are likely class-consistent with the centroid v_i. Under this assumption, the block partition of A,

$$A = \begin{bmatrix} A_{11} & \cdots & A_{1K} \\ \vdots & \ddots & \vdots \\ A_{K1} & \cdots & A_{KK} \end{bmatrix}$$

has sparse and relatively small off-diagonal blocks $A_{k\ell}$ with $\ell \neq k$. That is, A approximates the block-diagonal matrix

$$A_0 = \text{diag}(A_{11}, \ldots, A_{KK}).$$

The diagonal blocks $\{A_{kk}\}$ may be not connected, that is, each A_{kk} can be further partitioned as $A_{kk} = \text{diag}\big(A_1^{(k)}, \ldots, A_{p_k}^{(k)}\big)$ without loss of generalities, where each $A_i^{(k)}$ is connected.

If the neighborhoods are small, the diagonal blocks $\{A_t^{(k)}\}$ of A, are sparse and the connection within blocks may be very weak. Diagonal rescaling such as $D^{-1/2}AD^{-1/2}$ in the normalized cutting can strengthen the small diagonal blocks in the spectral projection, where D is the diagonal matrix of the column vector

$$r(A) = (r_1(A), \ldots, r_n(A))^T \quad \text{with} \quad r_i(A) = a_{i1} + \cdots + a_{in}.$$

However, if the neighborhoods are relatively large, each A_{kk} has a dominant block, say $A_1^{(k)}$, that covers most of class-consistent points. The diagonal rescaling may overly strengthen very small blocks and depress the dominant block. Based on the rescaled graph, the spectral method may result in a wrong projection.

To match the neighborhood size suitably, we suggest an adaptive scaling to the adjacency matrix A as

$$G = D^{-\beta/2}AD^{-\beta/2},$$

where the parameter $\beta \in [0, 1]$ tunes the diagonal scaling to match the neighborhood size: the larger the neighborhood size is, the smaller β is. Basically, β tunes the distributions of the K eigenvectors of the rescaled graph G as show below.

The property of the spectral projection on G will be exploited, utilizing a useful structure behind the block-partition of $G = (G_{k\ell})$ under the partition of $A = (A_{k\ell})$ and $A_k = [A_{k1}, \ldots, A_{kK}]$, where

$$G_{k\ell} = D_k^{-\beta/2} A_{k\ell} D_\ell^{-\beta/2}, \quad D_k = \text{diag}(r(A_k)),$$

and $r(A_k) = (r_1(A_k), \ldots, r_{n_k}(A_k))^T$. The key ideal is to take G as an approximate of the block diagonal matrix

$$G_0 = \text{diag}(\bar{G}_{11}, \ldots, \bar{G}_{KK}), \text{ where } \bar{G}_{kk} = \bar{D}_k^{-\beta/2} A_{kk} \bar{D}_k^{-\beta/2}, \quad \bar{D}_k = \text{diag}(r(A_{kk})),$$

due to the two observations: Good approximation of G to G_0, and spatial structures of the eigenvectors U_0 of G_0 corresponding to the K largest eigenvector. These two properties help us to exploit the class information from the K largest eigenvectors (the eigenvectors corresponding to the largest eigenvalues) of the graph G, via a special clustering approach that will be given in the later section. Below, we will show these propositions.

Approximation

The following term will be used in the error estimation.

$$A_k^c = [A_{k1}, \ldots, A_{k,k-1},\ 0,\ A_{k,k+1}, \ldots, A_{kK}],$$

$$\rho = (\rho_1; \ldots; \rho_K), \quad \rho_k = \Big(\frac{r_1(A_k^c)}{r_1(A_{kk})}; \ldots; \frac{r_{n_k}(A_k^c)}{r_{n_k}(A_{kk})}\Big),$$

$$\rho^{(\beta)} = \big(\rho_1^{(\beta)}; \ldots; \rho_K^{(\beta)}\big), \quad \rho_k^{(\beta)} = \Big(\frac{r_1(A_k^c)}{r_1^\beta(A_{kk})}; \ldots; \frac{r_{n_k}(A_k^c)}{r_{n_k}^\beta(A_{kk})}\Big).$$

where the semicolon means a column link. Clearly, $\rho_k^{(\beta)} = \rho_k$ when $\beta = 1$; or $\rho_k^{(\beta)} = (r_1(A_k^c); \ldots; r_{n_k}(A_k^c))$ if $\beta = 0$. Generally, $\rho_k^{(\beta)}$ and ρ_k are small if the class-consistent neighborhoods are estimated well.

Lemma 1. *The error matrix $E = G - G_0$ is bounded as $\|E\|_2 \le (1+\beta)\|\rho^{(\beta)}\|_\infty$.*

Proof. Representing the vector $r(A_k)$ as $r(A_k) = r(A_{kk}) + r(A_{kk}^c) = r(A_{kk}) \odot (1 + \rho_k)$,

$$D_k = \bar{D}_k J_k, \quad J_k = I + \mathrm{diag}(\rho_k).$$

and $G_{kk} = J_k^{-\beta/2} \bar{G}_{kk} J_k^{-\beta/2}$. Write $J_k^{-\beta/2} = I - \Delta_k$ with $\Delta_k = I - J_k^{-\beta/2}$ for simplicity. Then,

$$G_{kk} - \bar{G}_{kk} = (I - \Delta_k)\bar{G}_{kk} J_k^{-\beta/2} - \bar{G}_{kk} = \bar{G}_{kk}(J_k^{-\beta/2} - I) - \Delta_k \bar{G}_{kk} J_k^{-\beta/2} = -\bar{G}_{kk}\Delta_k - \Delta_k \bar{G}_{kk} J_k^{-\beta/2}.$$

Hence, by $\|J_k^{-\beta/2}\|_2 \le 1$ and using $\lambda_1(AB) = \lambda_1(BA)$ and $\lambda_1(C) \le \|r(C)\|_\infty$, we see that

$$\begin{aligned}
\|G_{kk} - \bar{G}_{kk}\|_2 &\le 2\|\Delta_k \bar{G}_{kk}\|_2 = 2\|\Delta_k \bar{D}^{-\beta/2} A_{kk} \bar{D}^{-\beta/2}\|_2 \\
&= 2\lambda_1\big(\Delta_k \bar{D}^{-\beta/2} A_{kk} \bar{D}^{-\beta/2}\big) = 2\lambda_1\big(\Delta_k \bar{D}^{-\beta} A_{kk}\big) \le 2\|r(\Delta_k \bar{D}^{-\beta} A_{kk})\|_\infty \\
&= 2\|\big(I - (I + \mathrm{diag}(\rho_k))^{-\beta/2}\big)\mathrm{diag}(r(A_{kk}))^{-\beta} r(A_{kk})\|_\infty.
\end{aligned}$$

By $0 \le 1 - (1+t)^{-\beta/2} \le \frac{\beta}{2} t$, it follows that

$$\|G_{kk} - \bar{G}_{kk}\|_2 \le \beta \max_i (\rho_k)_i (r_i(A_{kk}))^{1-\beta} = \beta \max_i (\rho_k^{(\beta)})_i = \beta \|\rho_k^{(\beta)}\|_\infty.$$

Hence, the block diagonal part E_0 of E is bounded as

$$\|E_0\|_2 = \max_k \|G_{kk} - \bar{G}_{kk}\|_2 \le \beta \|\rho^{(\beta)}\|_\infty$$

We also have the estimation on the off-block diagonal part E_0^c:

$$\|E_0^c\|_2 = \|D^{-\beta/2}[A_1^c; \cdots; A_K^c] D^{-\beta/2}\|_2 = \lambda_1(D^{-\beta}[A_1^c; \cdots; A_K^c]) \le \max_{k,i} \frac{r_i(A_k^c)}{r_i^\beta(A_k)} = \|\rho^{(\beta)}\|_\infty$$

Therefore, $\|E\|_2 \le \|E_0\|_2 + \|E_0^c\|_2 \le (1+\beta)\|\rho^{(\beta)}\|_\infty$. □

Structure of the eigenvectors of G

The block-diagonal G_0 has K sparse and nonnegative eigenvectors. Practically, since $\bar{G}_{kk}$ is nonnegative, by Perron-Frobenius theorem[49], it has a nonnegative eigenvector $\bar{u}_k$ corresponding to its largest eigenvalue $\lambda_1(\bar{G}_{kk})$. The eigenvector can be positive when $\bar{G}_{kk}$ is connected. Hence, let $u_k^{(0)}$ be the the sparse vector taking $\bar{u}_k$ as its nonzero piece with the index set as that of A_{kk} in A. Then $\{(\lambda_1(\bar{G}_{kk}), u_k^{(0)})\}$ are K eigen-pairs of G. Hence, $U_0 = [u_1^{(0)}, \ldots, u_K^{(0)}]$ has K row-blocks $\{u_k^{(0)} e_k^T\}$, where e_k is the k-th column of the identity matrix of order K.

For the K eigenvectors U of G corresponding to the K largest eigenvalues, the following lemma shows that there is an orthogonal matrix Q such that UQ has the structure as U_0 approximately.

Lemma 2. *Let $U = [u_1, \ldots, u_K]$ be the K eigenvectors of G corresponding to the K largest eigenvalues. Then there is an orthogonal matrix Q such that*

$$\|U_0 - UQ\|_F \le \frac{2\sqrt{2}\min\{\sqrt{K}\|E\|_2, \|E\|_F\}}{\lambda_K(G_0) - \lambda_{K+1}(G_0)}. \tag{1}$$

Generally, if $\min_k \lambda_1(\bar{G}_{kk}) > \max_k \lambda_2(\bar{G}_{kk})$, then

$$\lambda_K(G_0) - \lambda_{K+1}(G_0) = \min_k \lambda_1(\bar{G}_{kk}) - \max_k \lambda_2(\bar{G}_{kk}).$$

We omit the proof since it is an application of the perturbation theorem of invariant subspace given in[50] for symmetric matrices.

Clustering and accuracy

If each A_{kk} is connected, all the entries of $\bar{u}_k$ are positive. Hence, the ground-truth classes $\{C_k^*\}$ can be exactly separated according to the labeling

$$\ell_i^* = \arg\max_k u_{ik}^{(0)}.$$

That is, $C_k^* = \{i : \ \ell_i^* = k\}$. Thus, as soon as Q is available, the true classes can also be well estimated as $C_k = \{i : \ \ell_i = k\}$ according to the clustering rule

$$\ell_i = \arg\max_k |(UQ)_{ik}|, \quad i = 1, \cdots, n. \tag{2}$$

The following theorem gives an estimation to the accuracy according to above clustering rule.

Theorem 1. *Let $\varepsilon = \beta\|\rho\|_F + \tau^{\beta/2}\|\rho^{(\beta)}\|_F$, $\{\eta_p^2\}$ are the ordered squared entries of UQ in ascending order, and*

$$p = \arg\max\Big\{p : \ \eta_1^2 + \cdots + \eta_p^2 \le \frac{8\varepsilon^2}{\delta_K^2(G_0)}\Big\}. \tag{3}$$

If each $\bar{G}_{kk}$ is connected and $\delta(G_0) > 0$, then there are at most p points miss-clustered according to the clustering rule (2),

Proof. Let $UQ = (\tilde{u}_{ik})$ and $\mathcal{I}_k = \big\{i \in C_k^* : \ |\tilde{u}_{ik}| \le \max_{\ell\neq k} |\tilde{u}_{i\ell}|\big\}$. Only those vertices with indices in $\mathcal{I} = \cup_k \mathcal{I}_k$ may be miss-labeld by (2). By definition,

$$\sum_k \sum_{i\in\mathcal{J}_k} \tilde{u}_{ik}^2 \le \|U_0 - UQ\|_F^2 \le \frac{8\varepsilon^2}{\delta_K^2(G_0)}.$$

Since only the smallest p squared entries have a sum not lager than $\frac{8\varepsilon^2}{\delta_K^2(G_0)}$, we conclude that $|\mathcal{J}| = \sum_k |\mathcal{I}_k| \le p$, completing the proof. □

Note S4. Evaluation metrics

Clustering Evaluation metrics

We used three commonly used clustering accuracy metrics, including Accuracy (ACC) , Adjusted Rand Index (ARI)[51], and Normalized Mutual Information (NMI)[52]. These metrics assess the similarity between the clustering results and the real label of each dataset.

Given a set S of n elements, and two clusters or partitions of these elements, namely $X = \{X_1, \cdots, X_r\}$ and $Y = \{Y_1, \cdots, Y_s\}$ with $r \ge s$. We defined $n_{ij} = \text{card}\{X_i \bigcap Y_j\}$, $n_{i\cdot} = \sum_{j=1}^{s} n_{ij} = \text{card}\{X_i\}$, and $n_{\cdot j} = \sum_{i=1}^{r} n_{ij} = \text{card}\{Y_j\}$, where card$\{\cdot\}$ denotes the size of a set. The ACC measures the proportion of correctly assigned data points and is calculated as the average of the maximum number of data points assigned to the same cluster:

$$\text{ACC} = \frac{1}{n}\sum_{i=1}^{r} \max_j(n_{ij}). \tag{4}$$

The calculation of the Adjusted Rand Index (ARI) involves the use of the combination number $\binom{n}{2}$, which represents the total number of possible pairs that can be formed from a set of n data points. Specifically, the ARI is calculated as:

$$\text{ARI} = \frac{\sum_{i=1}^{r}\sum_{j=1}^{s}\binom{n_{ij}}{2} - \Big[\sum_{i=1}^{r}\binom{n_{i\cdot}}{2}\sum_{j=1}^{s}\binom{n_{\cdot j}}{2}\Big]/\binom{n}{2}}{\frac{1}{2}\Big[\sum_{i=1}^{r}\binom{n_{i\cdot}}{2} + \sum_{j=1}^{s}\binom{n_{\cdot j}}{2}\Big] - \Big[\sum_{i=1}^{r}\binom{n_{i\cdot}}{2}\sum_{j=1}^{s}\binom{n_{\cdot j}}{2}\Big]/\binom{n}{2}}. \tag{5}$$

Finally, the definition of NMI is the normalization of Mutual Information(MI). NMI takes into account the entropy between partitions to improve the accuracy of the comparison as

$$H(X) = -\frac{1}{n}\sum_{i=1}^{r} n_{i\cdot}\log\Big(\frac{n_{i\cdot}}{n}\Big) \quad H(Y) = -\frac{1}{n}\sum_{j=1}^{s} n_{\cdot j}\log\Big(\frac{n_{\cdot j}}{n}\Big), \tag{6}$$

and the definition of NMI is

$$\text{NMI} = \frac{2\sum_{i=1}^{r}\sum_{j=1}^{s} n_{ij}\left[\log(nn_{ij}) - \log(n_{i\cdot}n_{\cdot j})\right]}{n(H(X) + H(Y))}. \tag{7}$$

Visualization Evaluation metrics

To assess the quality of dimensionality reduction and visualization techniques, we employed the Silhouette Coefficient[53]. While primarily used for clustering evaluation, SC can also assess the quality of dimensionality reduction.

The Silhouette Coefficient for a single sample is given as:

$$s(i) = \frac{b(i) - a(i)}{\max\{a(i), b(i)\}} \tag{8}$$

where $a(i)$ is the mean distance between a sample and all other points in the same class (intra-class distance), and $b(i)$ is the mean distance between a sample and all other points in the next nearest cluster (nearest-neighbor inter-class distance).

The Silhouette Coefficient for a set of samples is given as the mean of the Silhouette Coefficient for each sample:

$$SC = \frac{1}{n}\sum_{i=1}^{n} s(i) \tag{9}$$

where n is the total number of samples.

The Silhouette Coefficient ranges from -1 to 1, where a higher value indicates that the object is well-matched to its own cluster and poorly-matched to neighboring clusters. This metric provides insight into both the cohesion within clusters and the separation between clusters in the low-dimensional representation.

Graph quality metrics

Graph score is a metric for evaluating the quality of weighted graph structures, combining precision and recall to measure the consistency between graph connections, their weights, and node categories. For a weighted graph $G(V, E, A)$, where V is the set of nodes, E is the set of edges, A is the set of edge weights, and C is the set of node categories, the graph score is defined as:

$$\text{Score}(G) = \text{Precision}(G) \odot \text{Recall}(G)$$

where $\odot$ represents element-wise multiplication (Hadamard product),

$$\text{Precision}(G)_i = \frac{\left[\sum_j A_{ij} \cdot \mathbb{I}(C(i) = C(j))\right]}{\left[\sum_j A_{ij}\right]}$$

$$\text{Recall}(G)_i = \frac{\left[\sum_j A_{ij} \cdot \mathbb{I}(C(i) = C(j))\right]}{\left[\sum_j \mathbb{I}(C(i) = C(j))\right]}$$

Here, A_{ij} is the element in the adjacency matrix representing the weight of edge (i, j), $\mathbb{I}(\cdot)$ is the indicator function, $C(i)$ represents the category of node i, and $[\,]/[\,]$ denotes element-wise division. Score(G) is a vector where each element corresponds to the score of a node.

Graph score is a metric for detecting the quality of weighted graphs, assessing the consistency between the graph structure and node category labels. A high score indicates that the graph's connection patterns highly align with the distribution of node categories: nodes of the same category tend to be closely connected by high-weight edges, while connections between different categories are weaker or absent. This metric combines precision (category consistency of existing connections) and recall (completeness of intra-category connections), providing a comprehensive measure of the graph's overall quality. It is particularly suitable for evaluating graphs with evident community structures or for validating the effectiveness of graph construction algorithms.

Note S5. RNA-seq data sets

GTEx Brain data set

Below is a description of the eight anatomical classes, with each paragraph discussing the role of the regions included in that class. The original data were labeled with 13 anatomical regions, but these regions were

reclassified into 8 broader categories. Note that the cells in the original 13 anatomical regions may originate from different donors. Some regions, such as parts of the cortex, overlap with areas grouped under other categories. Similarly, there is overlap between the Cerebellar Hemisphere and the Cerebellum. Due to these overlaps, making a strict separation between such regions is often not possible and may not be meaningful in the context of single-cell analysis.

- **Cortex:** This group includes regions such as the Anterior Cingulate Cortex and the Frontal Cortex. These cortical areas are fundamental for high-level cognitive processes including decision making, emotional regulation, attention, and conflict monitoring. The anterior cingulate cortex is especially known for its role in error detection and regulating emotional responses, while the frontal cortex is pivotal for planning, executive control, and social behavior[54].
- **Basal Ganglia:** In this group, key regions include the Putamen (basal ganglia) along with other dorsal striatal structures such as the Caudate. The basal ganglia are central to the initiation and control of voluntary movements, habit formation, and aspects of reward processing. Although these regions share similar cell types (mainly GABAergic medium spiny neurons), the Putamen is particularly involved in motor planning and execution. It differs from regions like the nucleus accumbens (not included in this group) by its primary role in motor function rather than in reward and motivational processing[55,56].
- **Cerebellum:** This class comprises both the Cerebellar Hemisphere and the Cerebellum proper. The cerebellum is traditionally recognized for coordinating movement, balance, and motor learning, and it also contributes to cognitive processes such as timing and error correction. The cerebellar hemisphere is mainly associated with lateralized motor and cognitive functions, while the midline structures play a key role in regulating axial and postural control[57].
- **Amygdala:** The Amygdala is a core limbic structure essential for processing emotional stimuli, particularly fear and anxiety, and for forming emotional memories. It interacts closely with cortical and hippocampal networks to assign affective significance to sensory inputs and to guide behavior based on emotional cues[58].
- **Hippocampus:** The Hippocampus is vital for the formation and consolidation of new memories and plays an essential role in spatial navigation. Its complex circuitry—including subfields such as CA1, CA3, and the dentate gyrus—underpins its capacity for neuroplasticity, which is fundamental to learning and memory processes[59].
- **Hypothalamus:** The Hypothalamus regulates a variety of homeostatic processes including hunger, thirst, body temperature, and circadian rhythms. It is also critical for controlling the sleep-wake cycle through its coordination of autonomic and endocrine responses. Various hypothalamic nuclei work together to maintain the body's internal balance and modulate responses to stress and environmental changes[60].
- **Spinal Cord:** Although not part of the brain, the Spinal Cord is an essential component of the central nervous system. It acts as the primary pathway for transmitting motor commands from the brain to the body and relaying sensory information upward. It plays a key role in reflex actions and in the execution of coordinated movement patterns.
- **Substantia Nigra:** The Substantia Nigra, located in the midbrain, is a major source of dopamine for the basal ganglia circuitry. Its dopaminergic neurons are critical for modulating motor control, influencing both movement initiation and execution. Dysfunction in this region is closely associated with movement disorders such as Parkinson's disease[61].

The Zeisel Dataset

The Zeisel dataset, as reported in[62], contains transcriptomic profiles for 3005 single cells isolated from the mouse cortex and hippocampus. In this study, the cells were classified into seven distinct cell types based on their gene expression profiles. Below is a description of each of these seven classes and the roles they play:

- **astrocytes_ependymal:** This class includes both astrocytes and ependymal cells. Astrocytes provide metabolic support, modulate synaptic activity, and help maintain the extracellular environment, while ependymal cells are involved in the production and circulation of cerebrospinal fluid.
- **endothelial-mural:** This group is composed of endothelial cells along with associated mural cells (such as pericytes and smooth muscle cells). These cells are essential for forming and maintaining the blood-brain barrier and regulating cerebral blood flow.

- **interneurons:** Interneurons are primarily inhibitory neurons that regulate local circuit activity. They play a critical role in balancing excitation and inhibition within neural networks, thereby ensuring proper signal modulation.
- **microglia:** Microglia are the resident immune cells of the central nervous system. They act as the primary mediators of immune responses in the brain by clearing debris, pathogens, and damaged cells, and by modulating inflammatory processes.
- **oligodendrocytes:** Oligodendrocytes are responsible for myelinating axons in the central nervous system. Myelination increases the speed and efficiency of electrical signal conduction along neuronal processes.
- **pyramidal CA1:** This class represents the excitatory pyramidal neurons found in the CA1 region of the hippocampus. These neurons are critical for memory formation and spatial navigation, as they integrate and transmit information within hippocampal circuits.
- **pyramidal SS:** Pyramidal neurons in this group are located in the somatosensory (SS) cortex. They play an important role in processing sensory information and are key components of cortical excitatory circuits.

The Darmanis Dataset

The Darmanis dataset, as described in Darmanis et al. (2015)[63], comprises transcriptomic profiles for 466 single cells isolated from the human brain. In this study, the cells were classified into nine distinct cell types. Below is a description of each cell type and their general functional roles:

- **OPC:** Oligodendrocyte Progenitor Cells (OPCs) are precursor cells that give rise to oligodendrocytes. They play a critical role in the development and maintenance of myelination in the brain.
- **astrocytes:** Astrocytes are glial cells that provide metabolic and structural support to neurons, regulate extracellular ion balance, and modulate synaptic transmission and plasticity.
- **endothelial:** Endothelial cells form the lining of blood vessels and are crucial for maintaining the blood-brain barrier, thus controlling the passage of substances between the bloodstream and the brain.
- **fetal_quiescent:** This category includes fetal brain cells that are in a quiescent (non-dividing) state. These cells are thought to represent a more mature, differentiated state within the developing brain.
- **fetal_replicating:** In contrast, the fetal_replicating cells are actively dividing. They represent a proliferative population in the fetal brain, contributing to brain development.
- **hybrid:** The hybrid class comprises cells that exhibit mixed or intermediate gene expression profiles. These cells may represent transitional states between defined cell types.
- **microglia:** Microglia are the resident immune cells of the brain. They monitor the environment for signs of infection or injury and play key roles in inflammation and tissue repair.
- **neurons:** Neurons are the primary excitatory cells in the brain responsible for transmitting electrical signals, processing information, and forming complex neural networks.
- **oligodendrocytes:** Oligodendrocytes are specialized glial cells responsible for producing myelin, the insulating sheath that increases the speed and efficiency of electrical signal transmission along axons.

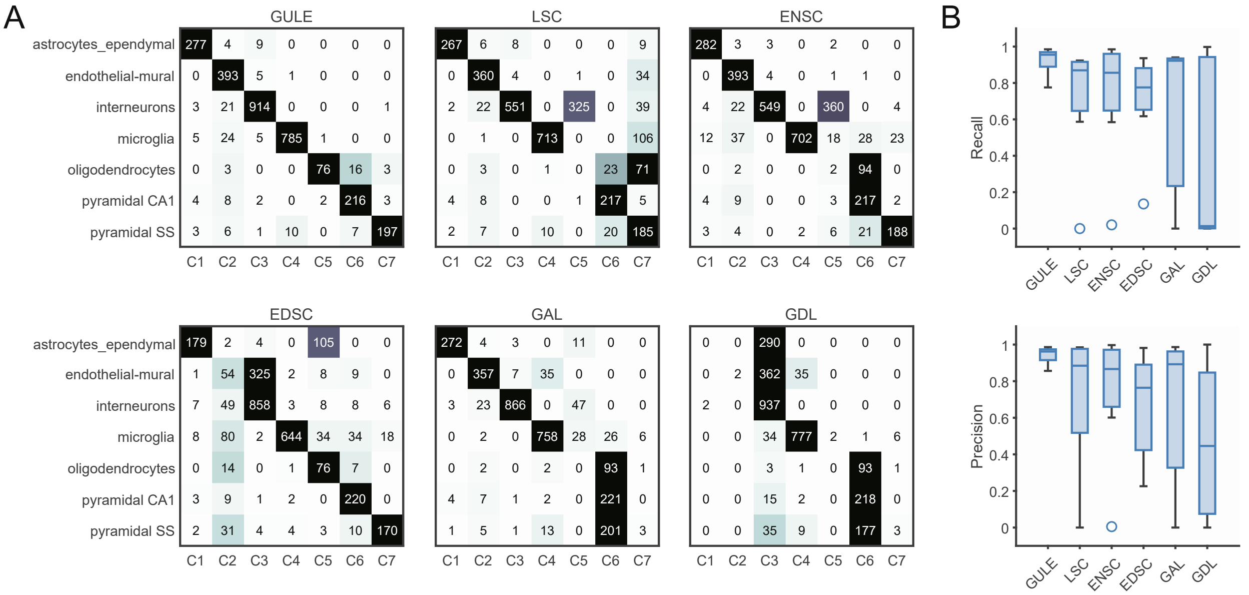

Figure S1: **Performance comparison of brain cell clustering methods on the Zeisel mouse brain dataset.** (A). Confusion matrices of different clustering methods on the Zeisel mouse brain dataset containing 3005 cells from seven major cell types. The rows represent true cell types and columns represent predicted clusters. (B) Recall and precision of each method.

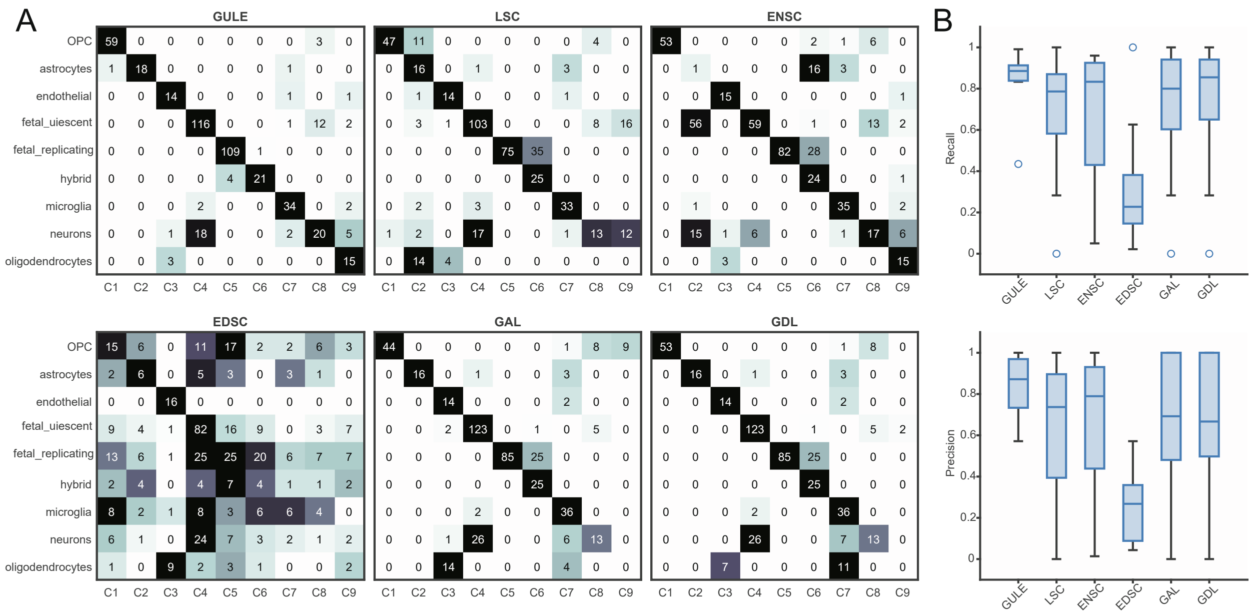

Figure S2: **Performance comparison of brain cell clustering methods on the Darmanis dataset.** (A). Confusion matrices of different clustering methods on the Darmanis human brain dataset containing 466 cells from nine major cell types. The rows represent true cell types and columns represent predicted clusters. (B) Recall and precision of each method.

Table S1: Characteristics of Datasets Used in Experimental Evaluation. Max CS = Maximum class size, Min CS = Minimum class size.

Data Set	Field	# Instance	# Features	# Classes	Max CS	Min CS	Distance Metric
Basehock	Text	1993	4862	2	999	994	Euclidean
COIL100	Object Image	7200	1024	100	72	72	Cityblock
COIL20	Object Image	1440	1024	20	72	72	Cityblock
Compounded	Synthetic	142	2	2	92	50	Cityblock
Control	Time seris	600	60	6	100	100	Euclidean
Entangled	Synthetic	312	2	3	106	101	Cityblock
Fashion	Fashion Image	70000	784	10	7000	7000	Spearman
HAR	Activity Recognition	10299	561	6	1944	1406	Euclidean
Iris	Plant	150	4	3	50	50	Euclidean
MNIST	Handwritten Digit	10000	784	10	1135	892	Cosine
MNIST70K	Handwritten Digit	70000	784	10	7877	6313	Cosine
MNIST-PT	Handwritten Digit	70000	500	10	7877	6313	Cosine
Olive	Face	400	4096	40	10	10	Euclidean
ORL	Face	400	10304	40	10	10	Euclidean
Pcmac	Text	1943	3289	2	982	961	Euclidean
Pendigit	Text	10992	16	10	1144	1055	Cityblock
PIE	Face	2856	1024	68	42	42	Cosine
Relathe	Text	1427	4322	2	779	648	Cityblock
Rounded	Synthetic	300	2	3	110	93	Cityblock
Satilite	remote sensing	6435	36	6	1533	626	Euclidean
Seed	Plant	210	7	3	70	70	Euclidean
Sports	Text	1000	59	2	635	365	Euclidean
UMist	Face	565	644	20	48	19	Cityblock
USPS	Handwritten Digit	9298	256	10	1553	708	Euclidean

Table S2: Web Sources of Datasets Used in Experimental Evaluation

Data Set	Website
Basehock	https://jundongl.github.io/scikit-feature/algorithms.html
COIL100	https://www.cs.columbia.edu/CAVE/software/softlib/coil-100.php
COIL20	https://www.cs.columbia.edu/CAVE/software/softlib/coil-20.php
Compounded	http://cs.joensuu.fi/sipu/datasets/
Control	https://archive.ics.uci.edu/ml/datasets/
Fashion	https://github.com/zalandoresearch/fashion-mnist
Entangled	http://cs.joensuu.fi/sipu/datasets/
HAR	https://archive.ics.uci.edu/ml/datasets/
Iris	https://archive.ics.uci.edu/ml/datasets/
MNIST	http://yann.lecun.com/exdb/mnist/
MNIST-P	http://vision.jhu.edu/code/
Olive	http://cam-orl.co.uk/facedatabase.html
ORL	http://cam-orl.co.uk/facedatabase.html
Pcmac	https://jundongl.github.io/scikit-feature/algorithms.html
Pendigit	https://archive.ics.uci.edu/ml/datasets/
PIE	http://www.cs.cmu.edu/afs/cs/project/PIE/MultiPi/Multi-Pie/Home.html
Relathe	https://jundongl.github.io/scikit-feature/algorithms.html
Rounded	http://cs.joensuu.fi/sipu/datasets/
Satilite	https://archive.ics.uci.edu/ml/datasets/
Seed	https://archive.ics.uci.edu/ml/datasets/
Sports	https://archive.ics.uci.edu/ml/datasets/
UMist	https://cs.nyu.edu/\textasciitilde roweis/data.html
USPS	https://web.stanford.edu/\textasciitilde hastie/StatLearnSparsity_files/DATA/zipcode.html

Table S3: Clustering performance (measured by ACC) of GULE and compared methods

Data Sets	GULE	SCSC	GAL	GDL	EDSC	EnSC	LSC	S-STSC	STSC	kmeans
Basehock	96.3	50.1	50.5	50.3	50.1	50.5	59.2	95.5	53.8	62.8
COIL100	94.3	73.2	69.1	70.5	47.0	57.3	50.7	66.1	46.1	52.0
COIL20	100.0	92.9	86.2	79.9	77.6	78.8	80.8	84.9	50.6	66.0
Control	97.5	49.7	83.3	92.3	53.2	52.7	79.3	66.0	55.5	56.8
HAR	76.3	64.3	55.2	18.9	35.6	67.5	71.7	63.6	51.0	70.6
Iris	96.0	86.7	90.0	69.3	52.7	97.3	89.3	96.7	90.7	96.7
MNIST	94.3	78.9	79.7	85.0	31.8	55.9	79.4	67.3	40.4	56.5
Olive	75.0	64.8	66.3	58.3	67.8	68.8	67.8	69.3	67.8	56.3
ORL	91.8	74.5	80.0	73.3	21.0	74.8	73.8	81.0	77.8	63.8
Pcmac	84.5	50.6	50.7	50.6	57.3	54.3	56.5	58.4	50.0	55.4
Pendigit	89.8	87.2	73.2	39.1	84.0	73.5	87.5	88.9	84.3	71.7
PIE	100.0	52.8	39.0	49.7	83.4	83.4	58.7	75.1	23.9	23.3
Relathe	77.9	54.4	54.7	54.7	54.7	54.7	55.3	81.7	51.1	57.7
Satilite	80.2	62.5	79.8	81.8	53.6	61.1	64.6	63.3	71.0	69.2
Seed	89.5	91.9	88.6	81.9	61.4	87.6	82.9	87.6	86.7	89.5
Sports	76.6	78.3	74.7	63.3	50.6	71.7	66.9	76.0	68.2	75.6
UMist	96.3	66.0	61.2	73.8	55.4	45.7	56.3	53.1	40.5	48.1
USPS	97.0	74.4	77.2	66.0	58.5	60.3	78.0	66.7	46.6	67.6
Average	89.6	69.6	70.0	64.4	55.3	66.4	69.9	74.5	58.7	63.3

Table S4: Clustering performance (measured by NMI) of GULE and compared methods

Data Sets	GULE	SCSC	GAL	GDL	EDSC	EnSC	LSC	S-STSC	STSC	kmeans
Basehock	77.3	0.1	0.4	0.2	0.1	0.2	9.1	75.3	0.4	5.0
COIL100	97.7	88.8	87.1	90.2	73.6	81.4	75.3	86.1	72.9	76.3
COIL20	100.0	95.6	91.3	90.7	86.2	91.2	88.4	91.4	68.8	78.8
Control	92.6	58.1	76.6	87.1	63.4	63.2	72.4	70.5	70.2	74.2
HAR	71.1	65.6	59.8	0.1	33.2	71.5	73.7	65.5	56.7	67.6
Iris	87.0	74.6	78.7	59.7	57.9	91.3	74.0	89.8	77.9	89.8
MNIST	87.8	79.8	76.0	81.8	24.4	61.3	74.7	73.4	41.5	54.2
Olive	86.4	80.4	81.6	77.8	83.3	83.9	81.6	81.7	82.1	71.6
ORL	94.9	86.1	91.4	88.7	38.3	88.0	88.1	90.5	89.2	82.3
Pcmac	38.0	0.1	0.2	0.1	1.5	3.2	1.8	7.4	0.2	1.7
Pendigit	87.6	83.8	77.4	39.8	76.8	74.5	82.8	85.2	79.3	67.5
PIE	100.0	65.9	61.9	71.7	94.8	96.1	79.1	88.3	48.1	51.9
Relathe	23.7	0.1	0.0	0.2	0.2	0.0	2.5	33.9	0.0	1.2
Satilite	64.9	61.8	64.3	64.8	41.7	55.5	61.4	64.7	58.6	61.1
Seed	69.1	73.3	69.5	60.7	27.2	65.4	58.3	65.4	65.4	70.0
Sports	22.2	24.6	17.5	0.1	0.0	19.4	11.4	23.2	16.3	17.6
UMist	96.4	79.3	78.7	86.5	71.2	66.2	77.7	75.5	64.9	70.2
USPS	92.1	75.6	81.7	70.2	63.5	69.4	79.9	81.5	52.5	61.3
Average	77.2	60.7	60.8	53.9	46.5	60.1	60.7	69.4	52.5	55.7

Table S5: Clustering performance (measured by ARI) of GULE and compared methods

Data Sets	GULE	SCSC	GAL	GDL	EDSC	EnSC	LSC	S-STSC	STSC	kmeans
Basehock	85.9	0.0	0.0	0.0	0.0	0.0	3.3	82.9	0.5	6.5
COIL100	92.6	62.9	64.6	66.9	38.2	51.7	43.0	59.7	38.6	46.2
COIL20	100.0	90.4	82.6	72.2	72.7	77.4	75.1	81.3	42.3	61.8
Control	94.1	44.1	68.3	83.9	51.4	49.3	61.6	58.6	59.4	61.7
HAR	61.9	57.4	52.0	0.0	23.1	60.7	66.7	59.3	39.4	62.8
Iris	88.6	68.4	74.6	56.1	44.3	92.2	72.9	90.4	75.8	90.4
MNIST	88.0	73.5	71.2	79.2	12.4	45.2	68.3	61.9	26.1	42.2
Olive	65.7	50.1	53.5	43.8	56.7	59.3	53.9	57.4	56.0	37.0
ORL	85.8	61.6	75.3	67.8	5.9	67.9	67.6	74.0	71.3	54.2
Pcmac	47.6	0.0	0.0	0.0	2.1	0.7	1.7	2.8	0.0	1.1
Pendigit	81.5	75.3	62.3	20.2	70.9	61.7	76.9	79.3	70.1	57.0
PIE	100.0	21.8	22.6	34.4	82.9	86.1	49.7	68.7	13.4	13.9
Relathe	31.1	0.0	0.1	0.1	0.1	0.0	0.8	40.1	0.0	2.2
Satilite	65.8	53.1	66.9	68.0	36.8	49.0	51.5	54.1	49.4	53.6
Seed	72.1	77.7	70.2	56.6	27.5	67.6	55.7	67.0	65.0	71.7
Sports	28.2	31.9	24.2	0.0	0.0	18.7	11.3	27.0	12.9	25.9
UMist	93.7	52.8	56.2	69.8	41.0	35.9	48.5	47.7	31.9	40.4
USPS	94.1	62.4	73.3	63.9	47.8	50.1	73.2	67.7	34.7	53.6
Average	76.5	49.1	51.0	43.5	34.1	48.5	49.0	60.0	38.2	43.5

Table S6: Clustering accuracy ACC (%) of GULE and the deep networks methods. Blank in the table indicate that the dataset was not tested by the corresponding method.

Method	COIL-20	COIL-100	PIE	ORL	UMist	USPS	MNIST-T	Pendigit	MNIST	Fashion
JULE	100.0	91.6	100.0		80.9	95.0	96.1			
DSC	94.9	69.0		86.0						
DBC	79.3	77.5				74.3				
DDM			85.8			97.9	87.1		96.9	
GALA	82.3						74.3			
N2D						95.8	94.8	88.5		
DASC	96.4			88.3	76.9					
EDAE						87.3		87.3		
DSCNSS	96.3	71.4		89.2						
SADSC	97.5	84.5		91.3			88.3			
DCRSM	81.2	77.0								69.7
ODSC	97.5			88.0			81.2			
WEC	84.0								96.7	62.3
DCCF						85.5			97.4	62.1
DFCN						79.5				
EDESC										63.1
DCMF						79.7	87.8		90.2	61.7
DEC									84.3	60.0
LGC-AUM						97.5			98.7	65.4
RD-FKC	73.5		37.7	65.4		76.1				61.2
DPSC			82.4			80.0	97.6		97.6	
DeepGMM	94.6	86.3	95.7			98.5			98.7	63.5
DSCSC	97.9			90.8	81.8				85.1	
DSC-DAG	97.3	72.1		91.1	78.1					
GULE	100.0	96.0	100.0	93.0	96.5	97.1	94.5	89.8	95.6	66.4

Table S7: Stability of parameter k_0 on clustering accuracy

Data Sets	$k_0 = 0$	1	2	3	4	5	6	7	8	9	10
Basehock	95.6	95.9	96.2	96.6	96.7	96.3	96.6	96.8	96.8	96.5	96.9
COIL100	94.5	94.8	93.4	94.8	95.6	94.3	96.8	94.8	89.9	96.3	83.7
COIL20	99.7	100.0	93.5	100.0	100.0	100.0	100.0	100.0	100.0	99.9	100.0
Compounded	85.2	99.3	100.0	61.3	100.0	100.0	100.0	100.0	100.0	100.0	100.0
Control	67.0	76.7	69.3	90.0	96.3	97.5	97.7	98.3	98.0	97.8	97.2
Entangled	100.0	100.0	100.0	100.0	100.0	100.0	100.0	100.0	100.0	100.0	95.2
HAR	81.4	80.4	81.9	79.7	78.1	76.3	76.5	76.4	75.2	74.2	72.4
Iris	96.0	96.0	96.0	96.0	96.0	96.0	96.7	90.7	90.0	96.7	96.0
MNIST	93.6	93.8	93.9	93.9	94.2	94.3	94.4	93.0	91.4	90.2	90.4
Olive	65.5	71.5	70.0	73.3	75.0	75.0	74.0	72.8	73.0	72.5	74.0
ORL	73.8	83.3	84.5	88.0	89.0	91.8	92.0	86.5	87.3	87.5	87.5
Pcmac	81.7	83.4	84.7	85.3	84.4	84.5	85.3	85.7	85.9	86.3	86.1
Pendigit	89.6	89.7	89.6	89.8	89.9	89.8	89.9	89.8	90.0	89.8	89.8
PIE	94.5	95.1	97.5	99.2	99.9	100.0	100.0	100.0	100.0	100.0	98.5
Relathe	78.8	78.4	78.8	77.8	77.6	77.9	77.7	78.0	77.4	77.2	77.2
Rounded	71.0	98.3	90.3	82.3	72.0	99.3	88.7	88.7	88.7	88.7	88.7
Satilite	60.1	62.1	78.4	75.3	81.2	80.2	79.3	80.9	79.9	69.2	65.7
Seed	79.5	77.1	87.6	87.6	88.6	89.5	89.5	89.5	88.6	87.6	88.1
Sports	76.2	76.3	76.2	76.6	76.6	76.6	76.7	76.5	76.0	76.1	76.4
UMist	82.3	86.5	88.7	87.1	90.3	96.3	97.2	93.5	95.9	93.5	96.5
USPS	96.8	96.7	96.8	97.0	97.0	97.0	96.9	96.9	96.8	96.8	96.9
Average	83.9	87.4	88.0	87.2	89.4	91.1	90.8	89.9	89.6	89.4	88.4

Table S8: Stability of parameter α on clustering accuracy

Data Sets	$\alpha = 0$	1	2	3	4	5	6	7	8	9	10	11	12	13	14	15
Basehock	95.9	96.0	96.2	95.9	97.1	97.1	96.3	94.8	86.7	67.8	57.0	51.9	50.4	52.9	51.0	50.7
COIL100	68.2	66.7	69.9	74.6	87.1	91.4	94.7	95.9	94.3	94.3	96.0	87.3	78.0	59.7	44.0	24.3
COIL20	84.5	84.4	86.5	88.4	92.4	100.0	100.0	100.0	100.0	100.0	100.0	87.1	99.4	86.9	73.9	65.9
Compounded	57.0	57.0	57.7	57.0	56.3	100.0	100.0	100.0	100.0	100.0	100.0	100.0	100.0	98.6	97.9	98.6
Control	54.3	70.2	71.2	71.0	73.5	80.7	83.2	90.2	98.5	97.5	35.0	43.5	35.5	30.7	31.2	33.5
Entangled	38.8	46.8	65.7	67.6	76.3	100.0	100.0	100.0	100.0	100.0	100.0	100.0	100.0	100.0	100.0	99.7
HAR	70.4	70.3	70.6	72.3	74.8	76.0	76.3	78.2	75.6	61.1	42.0	50.4	39.1	41.4	45.3	31.7
Iris	90.0	90.0	90.7	90.7	92.7	96.0	96.0	96.0	96.0	96.0	96.0	96.0	96.0	64.7	75.3	73.3
MNIST	81.9	80.6	87.4	70.2	78.9	83.4	94.3	94.7	92.9	91.3	35.0	24.3	24.6	23.9	24.5	24.4
Olive	67.0	67.3	69.3	70.5	72.3	73.8	75.3	75.5	75.5	75.0	68.0	56.3	56.0	54.0	53.0	53.3
ORL	78.3	79.5	81.3	83.0	84.3	87.0	88.5	88.8	88.3	91.8	78.8	62.5	64.3	65.3	61.0	57.5
Pcmac	72.7	73.5	79.7	83.2	88.3	86.4	84.5	82.9	74.9	73.3	51.3	50.3	51.0	51.2	51.0	50.0
Pendigit	88.5	89.0	88.8	89.6	89.4	89.5	89.8	89.8	89.6	89.7	88.7	74.1	47.0	43.5	36.5	26.6
PIE	98.9	99.1	99.2	99.8	100.0	100.0	100.0	100.0	100.0	100.0	96.5	99.4	99.7	82.5	53.2	40.2
Relathe	77.6	78.8	80.2	80.7	81.0	80.5	77.9	73.4	59.1	78.8	54.2	50.1	51.2	51.4	50.7	51.2
Rounded	80.0	80.0	80.7	84.3	99.0	96.0	98.3	98.7	99.0	99.3	98.0	98.0	98.3	98.0	98.0	88.3
Satilite	65.7	65.4	65.6	66.4	61.5	61.5	80.2	56.5	71.1	63.5	55.5	40.6	34.7	34.2	32.2	38.8
Seed	92.9	92.4	92.4	91.9	91.4	91.0	90.0	89.5	89.5	89.5	89.5	89.5	71.0	66.2	44.8	53.8
Sports	76.5	76.2	76.2	76.0	76.5	76.3	76.6	76.8	75.4	74.5	73.4	56.6	53.3	53.7	51.6	51.3
UMist	56.8	58.8	63.0	72.0	82.8	83.4	88.7	90.6	91.0	96.3	95.4	93.8	80.5	69.2	48.7	45.3
USPS	78.6	78.6	78.8	79.8	80.8	96.7	97.0	96.7	80.0	96.4	69.6	35.6	31.9	31.4	35.2	27.7
Average	75.0	76.2	78.6	79.3	82.7	87.9	89.9	89.0	87.5	87.4	75.2	68.9	64.9	60.0	55.2	51.7

Table S9: Stability of parameter β on clustering accuracy

Data Sets	$\beta = 0$	0.1	0.2	0.3	0.4	0.5	0.6	0.7	0.8	0.9	1
Basehock	96.3	96.3	96.3	96.3	96.3	96.3	96.3	96.3	96.3	96.3	96.4
COIL100	94.3	94.6	94.7	94.6	94.8	94.8	94.4	94.0	92.9	92.2	89.4
COIL20	100.0	100.0	100.0	100.0	100.0	100.0	100.0	100.0	100.0	100.0	100.0
Compounded	100.0	100.0	100.0	100.0	100.0	100.0	100.0	100.0	100.0	100.0	100.0
Control	97.5	97.5	97.5	97.3	97.3	97.3	97.5	97.5	97.5	97.5	97.5
Entangled	100.0	100.0	100.0	100.0	100.0	100.0	100.0	100.0	100.0	100.0	100.0
HAR	76.3	76.2	76.2	76.2	76.1	76.1	76.1	76.1	76.0	73.1	52.8
Heart	71.6	71.6	71.6	71.6	71.6	71.6	71.6	71.6	71.6	71.6	54.8
Iris	96.0	96.0	96.0	96.0	96.0	96.0	96.0	96.0	96.0	96.0	96.0
MNIST	94.3	94.2	94.2	94.3	94.2	94.3	94.2	94.3	94.3	90.8	84.6
Olive	75.0	75.0	75.0	76.3	75.3	75.3	73.5	73.3	73.3	73.0	72.5
ORL	91.8	91.8	91.8	91.8	91.0	91.0	91.0	89.0	89.0	89.0	89.0
Pcmac	84.5	84.5	84.5	84.5	84.5	84.5	84.6	84.6	84.6	84.6	84.6
Pendigit	89.8	89.8	89.8	89.8	89.8	89.8	89.8	89.8	89.8	90.0	73.3
PIE	100.0	100.0	100.0	100.0	100.0	100.0	100.0	100.0	100.0	100.0	100.0
Relathe	78.0	78.0	78.1	78.1	78.1	78.1	78.1	78.1	78.3	78.3	78.3
Rounded	99.3	99.3	99.3	99.3	99.3	99.3	99.3	99.3	99.3	78.3	78.3
Satilite	80.2	80.3	80.3	68.5	68.5	68.4	68.4	67.7	56.1	55.7	56.2
Seed	89.5	89.5	89.5	89.5	89.5	89.5	89.5	89.5	89.5	89.5	89.5
Sports	76.6	76.6	76.6	76.6	76.6	76.6	76.5	76.5	76.6	76.6	76.6
UMist	95.6	95.6	95.8	95.8	95.8	95.8	95.8	92.4	92.4	92.4	92.4
USPS	97.0	97.0	97.0	97.0	97.0	97.0	97.0	97.0	97.0	97.0	97.0
Average	90.2	90.2	90.2	89.7	89.6	89.6	89.5	89.2	88.7	87.4	84.5

Table S10: Performance of distance selection. NSE = normalized squared Euclidean. NE = normalized Euclidean.

Data	Euclidean	NSE	Cityblock	Spearman	NE	Best Accuracy	Selected Accuracy	Gap to Best	Selected Distance
COIL100	0.86	0.86	0.94	0.81	0.88	0.94	0.94	0	Cityblock
COIL20	0.73	0.9	1	0.76	0.92	1	1	0	Cityblock
Control	0.98	0.75	0.92	0.66	0.98	0.98	0.98	0	Euclidean
HAR	0.65	0.76	0.47	0.46	0.76	0.76	0.76	0	NE
Iris	0.96	0.85	0.96	0.79	0.86	0.96	0.96	0	Euclidean
MNIST	0.88	N/A	0.92	0.92	0.94	0.94	0.94	0	NE
Olive	0.69	0.75	0.73	0.72	0.68	0.75	0.69	0.07	Euclidean
ORL	0.92	0.81	0.87	0.83	0.81	0.92	0.92	0	Euclidean
Pendigit	0.89	0.87	0.9	0.85	0.89	0.9	0.89	0.01	Euclidean
PIE	0.77	1	0.66	0.96	1	1	1	0	NSE
Satilite	0.8	0.57	0.68	0.45	0.5	0.8	0.8	0	Euclidean
Seed	0.81	0.9	0.8	0.61	0.88	0.9	0.9	0	NSE
UMist	0.87	0.86	0.96	0.88	0.79	0.96	0.87	0.09	Euclidean
USPS	0.97	0.96	0.8	0.79	0.97	0.97	0.97	0	Euclidean
Average	0.84	0.83	0.83	0.75	0.85	0.91	0.9	0.01	

References

1. Lloyd, S. (1982). Least squares quantization in pcm. IEEE transactions on information theory *28*, 129–137. https://doi.org/10.1109/TIT.1982.1056489.

2. Arthur, D., and Vassilvitskii, S. (2007). K-means++ the advantages of careful seeding. In Proceedings of the eighteenth annual ACM-SIAM symposium on Discrete algorithms. pp. 1027–1035. https://dl.acm.org/doi/10.5555/1283383.1283494.

3. Zelnik-Manor, L., and Perona, P. (2004). Self-tuning spectral clustering. Advances in neural information processing systems *17*. https://proceedings.neurips.cc/paper_files/paper/2004/file-/40173ea48d9567f1f393b20c855bb40b-Paper.pdf.

4. Kuang, D., Yun, S., and Park, H. (2015). Symnmf: nonnegative low-rank approximation of a similarity matrix for graph clustering. Journal of Global Optimization *62*, 545–574. https://doi.org/10.1007/s10898-014-0247-2.

5. Cai, D., and Chen, X. (2014). Large scale spectral clustering via landmark-based sparse representation. IEEE transactions on cybernetics *45*, 1669–1680. https://doi.org/10.1109/TCYB.2014.2358564.

6. Bai, L., Liang, J., and Zhao, Y. (2022). Self-constrained spectral clustering. IEEE Transactions on Pattern Analysis and Machine Intelligence *45*, 5126–5138. https://doi.org/10.1109/TPAMI.2022.3188160.

7. You, C., Li, C.G., Robinson, D.P., and Vidal, R. (2016). Oracle based active set algorithm for scalable elastic net subspace clustering. In Proceedings of the IEEE conference on computer vision and pattern recognition. pp. 3928–3937. https://openaccess.thecvf.com/content_cvpr-_2016/papers/You_Oracle_Based_Active_CVPR_2016_paper.pdf.

8. Ji, P., Salzmann, M., and Li, H. (2014). Efficient dense subspace clustering. In IEEE Winter conference on applications of computer vision. IEEE pp. 461–468. https://doi.org/10.1109/WACV.2014.6836065.

9. Zhang, W., Wang, X., Zhao, D., and Tang, X. (2012). Graph degree linkage: Agglomerative clustering on a directed graph. In Computer Vision–ECCV 2012: 12th European Conference on Computer Vision, Florence, Italy, October 7-13, 2012, Proceedings, Part I 12. Springer pp. 428–441. https://doi.org/10.1007/978-3-642-33718-5_31.

10. Yang, J., Parikh, D., and Batra, D. (2016). Joint unsupervised learning of deep representations and image clusters. In Proceedings of the IEEE conference on computer vision and pattern recognition. pp. 5147–5156. https://www.cv-foundation.org/openaccess/content_cvpr_2016/papers/Yang_Joint_Unsupervised_Learning_CVPR-_2016_paper.pdf.

11. Ji, P., Zhang, T., Li, H., Salzmann, M., and Reid, I. (2017). Deep subspace clustering networks. Advances in neural information processing systems *30*. https://proceedings.neurips.cc/paper_files/paper/2017/file/e369853df766fa44e1ed0ff613f563bdPaper.pdf.

12. Zhou, P., Hou, Y., and Feng, J. (2018). Deep adversarial subspace clustering. In Proceedings of the IEEE conference on computer vision and pattern recognition. pp. 1596–1604. https://openaccess.thecvf.com/content_cvpr_2018/papers/Zhou_Deep_Adversarial_Subspa-ce_CVPR_2018_paper.pdf.

13. Li, F., Qiao, H., and Zhang, B. (2018). Discriminatively boosted image clustering with fully convolutional auto-encoders. Pattern Recognition *83*, 161–173. https://doi.org/10.1016/j.patcog.2018.05.019.

14. Jabi, M., Pedersoli, M., Mitiche, A., and Ayed, I.B. (2019). Deep clustering: On the link between discriminative models and k-means. IEEE transactions on pattern analysis and machine intelligence *43*, 1887–1896. https://doi.org/10.1109/TPAMI.2019.2962683.

15. Park, J., Lee, M., Chang, H.J., Lee, K., and Choi, J.Y. (2019). Symmetric graph convolutional autoencoder for unsupervised graph representation learning. In Proceedings of the IEEE/CVF international conference on computer vision. pp. 6519–6528. https://openaccess.thecvf.com/content_ICCV_2019/papers/Park_Symmetric_Graph_Convolutional-_Autoencoder_for_Unsupervised_Graph_Representation_Learning_ICCV_2019_paper.pdf.

16. McConville, R., Santos-Rodriguez, R., Piechocki, R.J., and Craddock, I. (2021). N2d:(not too) deep clustering via clustering the local manifold of an autoencoded embedding. In 2020 25th international conference on pattern recognition (ICPR). IEEE pp. 5145–5152. https://doi.org/10.1109/ICPR48806.2021.9413131.

17. Affeldt, S., Labiod, L., and Nadif, M. (2020). Spectral clustering via ensemble deep autoencoder learning (sc-edae). Pattern Recognition *108*, 107522. https://doi.org/10.1016/j.patcog.2020.107522.

18. Chen, C., Lu, H., Wei, H., and Geng, X. (2023). Deep subspace image clustering network with self-expression and self-supervision. Applied Intelligence *53*, 4859–4873. https://doi.org/10.1007/s10489-022-03654-6.

19. Chen, Z., Ding, S., and Hou, H. (2021). A novel self-attention deep subspace clustering. International Journal of Machine Learning and Cybernetics *12*, 2377–2387. https://doi.org/10.1007/s13042-021-01318-4.

20. Shi, Z., and Zhao, H. (2023). Deep multi-view clustering based on reconstructed self-expressive matrix. Applied Sciences *13*, 8791. https://doi.org/10.3390/app13158791.

21. Valanarasu, J.M.J., and Patel, V.M. (2021). Overcomplete deep subspace clustering networks. In Proceedings of the IEEE/CVF winter conference on applications of computer vision. pp. 746–755. https://openaccess.thecvf.com/content/WACV2021/papers/Valanarasu_Overcomplete_Deep_Subspace_Clustering_Networks_WACV_2021_paper.pdf.

22. Cai, J., Zhang, Y., Wang, S., Fan, J., and Guo, W. (2024). Wasserstein embedding learning for deep clustering: A generative approach. IEEE Transactions on Multimedia. https://doi.org/10.1109/TMM.2024.3369862.

23. Cai, J., Wang, S., Xu, C., and Guo, W. (2022). Unsupervised deep clustering via contractive feature representation and focal loss. Pattern Recognition *123*, 108386. https://doi.org/10.1016/j.patcog.2021.108386.

24. Tu, W., Zhou, S., Liu, X., Guo, X., Cai, Z., Zhu, E., and Cheng, J. (2021). Deep fusion clustering network. In Proceedings of the AAAI Conference on Artificial Intelligence vol. 35. pp. 9978–9987. https://doi.org/10.1609/aaai.v35i11.17198.

25. Cai, J., Fan, J., Guo, W., Wang, S., Zhang, Y., and Zhang, Z. (2022). Efficient deep embedded subspace clustering. In Proceedings of the IEEE/CVF Conference on Computer Vision and Pattern Recognition. pp. 1–10. https://doi.ieeecomputersociety.org/10.1109/CVPR52688.2022.00012.

26. Hou, H., Ding, S., and Xu, X. (2022). A deep clustering by multi-level feature fusion. International Journal of Machine Learning and Cybernetics *13*, 2813–2823. https://doi.org/10.1007/s13042-022-01557-z.

27. Wickramasinghe, C., Marino, D., and Manic, M. (2021). Deep embedded clustering with resnets. In 2021 14th International Conference on Human System Interaction (HSI). IEEE pp. 1–6. https://doi.org/10.1109/HSI52170.2021.9538747.

28. Wang, T., Zhang, X., Lan, L., and Luo, Z. (2022). Local-to-global deep clustering on approximate uniform manifold. IEEE Transactions on Knowledge and Data Engineering *35*, 5035–5046. https://doi.org/10.1109/TKDE.2022.3144952.

29. Wu, X., Yu, Y.F., Chen, L., Ding, W., and Wang, Y. (2024). Robust deep fuzzy k-means clustering for image data. Pattern Recognition *153*, 110504. https://doi.org/10.1016/j.patcog.2024.110504.

30. Hu, W., Chen, C., Ye, F., Zheng, Z., and Du, Y. (2021). Learning deep discriminative representations with pseudo supervision for image clustering. Information Sciences *568*, 199–215. https://doi.org/10.1016/j.ins.2021.03.066.

31. Wang, J., and Jiang, J. (2021). Unsupervised deep clustering via adaptive gmm modeling and optimization. Neurocomputing *433*, 199–211. https://doi.org/10.1016/j.neucom.2020.12.082.

32. Peng, B., and Zhu, W. (2021). Deep structural contrastive subspace clustering. In Asian Conference on Machine Learning. PMLR pp. 1145–1160. https://proceedings.mlr.press/v157/peng21a/peng21a.pdf.

33. Yu, Z., Zhang, Z., Cao, W., Liu, C., Chen, C.P., and Wong, H.S. (2020). Gan-based enhanced deep subspace clustering networks. IEEE Transactions on Knowledge and Data Engineering *34*, 3267–3281. https://doi.org/10.1109/TKDE.2020.3025301.

34. Li, J., Cheng, K., Wang, S., Morstatter, F., Trevino, R.P., Tang, J., and Liu, H. (2018). Feature selection: A data perspective. ACM Computing Surveys (CSUR) *50*, 94. https://doi.org/10.1145/3136625.

35. Nene, S.A., Nayar, S.K., and Murase, H. (1996). Columbia object image library. Columbia University. https://www.cs.columbia.edu/CAVE/software/softlib/coil-20.php.

36. Alcock, R.J., Manolopoulos, Y. et al. (1999). Time-series similarity queries employing a feature-based approach. In 7th Hellenic conference on informatics. pp. 27–29. https://machinelearning101.pbworks.com/f/TimeSeriesData10.1.1.79.1572.pdf.

37. Xiao, H., Rasul, K., and Vollgraf, R. (2017). Fashion-mnist: a novel image dataset for benchmarking machine learning algorithms. Preprint at arXiv. https://doi.org/10.48550/arXiv.1708.07747.

38. Anguita, D., Ghio, A., Oneto, L., Parra, X., and Reyes-Ortiz, J.L. (2013). A public domain dataset for human activity recognition using smartphones. In 21th European Symposium on Artificial Neural Networks, Computational Intelligence and Machine Learning, ESANN 2013. ESANN pp. 437–442. http://hdl.handle.net/2117/20897.

39. Fisher, R.A. (1936). The use of multiple measurements in taxonomic problems. Annals of eugenics *7*, 179–188. https://doi.org/10.1111/j.1469-1809.1936.tb02137.x.

40. LeCun, Y., Bottou, L., Bengio, Y., and Haffner, P. (1998). Gradient-based learning applied to document recognition. Proceedings of the IEEE *86*, 2278–2324. https://doi.org/10.1109/5.726791.

41. Samaria, F.S., and Harter, A.C. (1994). Parameterisation of a stochastic model for human face identification. In Proceedings of 1994 IEEE Workshop on Applications of Computer Vision. IEEE pp. 138–142. https://doi.org/10.1109/ACV.1994.341300.

42. Alimoglu, F., and Alpaydin, E. (1996). Combining multiple classifiers for pen-based handwritten digit recognition. TAINN Proceedings pp. 38–41. https://doi.org/10.1109/ICDAR.1997.620583.

43. Sim, T., Baker, S., and Bsat, M. (2002). The cmu pose, illumination, and expression (pie) database. In Proceedings of Fifth IEEE International Conference on Automatic Face Gesture Recognition. IEEE pp. 53–58. https://doi.org/10.1109/AFGR.2002.1004130.

44. Srinivasan, A. (1993). Statlog (Landsat Satellite). UCI Machine Learning Repository. . https://doi.org/10.24432/C55887.

45. Charytanowicz, M., Niewczas, J., Kulczycki, P., Kowalski, P., and Lukasik, S. (2010). Seeds. UCI Machine Learning Repository. . https://doi.org/10.24432/C5H30K.

46. Rizk, Y., and Awad, M. (2018). Sports articles for objectivity analysis. UCI Machine Learning Repository. . https://doi.org/10.24432/C5801R.

47. Graham, D.B., and Allinson, N.M. (1998). Characterising virtual eigensignatures for general purpose face recognition. In Face Recognition: From Theory to Applications pp. 446–456.. Springer pp. 446–456. https://link.springer.com/chapter/10.1007/978-3-642-72201-1_25.

48. Hull, J.J. (1994). A database for handwritten text recognition research. IEEE Transactions on pattern analysis and machine intelligence *16*, 550–554. https://doi.org/10.1109/34.291440.

49. Schur, J. (1905). Zur theorie der vertauschbaren matrizen. Journal für die reine und angewandte Mathematik *1905*, 66–76. https://doi.org/10.1515/crll.1905.130.66.

50. Golub, G.H., and Van Loan, C.F. (2013). Matrix computations. JHU press. https://epubs.siam.org/doi/abs/10.1137/1.9781421407944.

51. Rand, W.M. (1971). Objective criteria for the evaluation of clustering methods. Journal of the American Statistical association *66*, 846–850. https://doi.org/10.1080/01621459.1971.10482356.

52. Pfitzner, D., Leibbrandt, R., and Powers, D. (2009). Characterization and evaluation of similarity measures for pairs of clusterings. Knowledge and Information Systems *19*, 361–394. https://doi.org/10.1007/s10115-008-0150-6.

53. Rousseeuw, P.J. (1987). Silhouettes: a graphical aid to the interpretation and validation of cluster analysis. Journal of computational and applied mathematics *20*, 53–65. https://doi.org/10.1016/0377-0427(87)90125-7.

54. Kandel, E.R., Schwartz, J.H., and Jessell, T.M. (2013). Principles of Neural Science. 5th ed.. McGraw-Hill. https://neurology.mhmedical.com/book.aspx?bookid=3024.

55. Albin, R.L., Young, A.B., and Penney, J.B. (1989). The functional anatomy of basal ganglia disorders. Trends in Neurosciences *12*, 366–375. https://doi.org/10.1016/0166-2236(89)90074-X.

56. DeLong, M.R. (1990). Primate models of movement disorders of basal ganglia origin. Trends in Neurosciences *13*, 281–285. https://doi.org/10.1016/0166-2236(90)90110-V.

57. Schmahmann, J.D. (2019). The cerebellum and cognition. Neuroscience Letters *688*, 62–75. https://doi.org/10.1016/j.neulet.2018.07.005.

58. LeDoux, J. (2000). Emotion circuits in the brain. Annual Review of Neuroscience *23*, 155–184. https://doi.org/10.4324/9780203825266.

59. Squire, L.R., and Zola-Morgan, S. (1991). The medial temporal lobe memory system. Science *253*, 1380–1386. https://doi.org/10.1126/science.1896849.

60. Saper, C.B., Chou, T.C., and Scammell, T.E. (2001). The sleep switch: hypothalamic control of sleep and wakefulness. Trends in Neurosciences *24*, 726–731. https://doi.org/10.1016/S0166-2236(00)02002-6.

61. Obeso, J.A., Rodriguez-Oroz, M.C., Benitez-Temino, B. et al. (2008). Functional organization of the basal ganglia: Therapeutic implications for parkinson's disease. Movement Disorders *23*, S548–S559. https://doi.org/10.1002/mds.22062.

62. Zeisel, A., Muñoz-Manchado, A.B., Codeluppi, S., Lönnerberg, P., La Manno, G., Jureus, A., Marques, S., Munguba, H., He, L., Betsholtz, C., Rolny, C., Castelo-Branco, G., Hjerling-Leffler, J., and Linnarsson, S. (2015). Cell types in the mouse cortex and hippocampus revealed by single-cell rna-seq. Science *347*, 1138–1142. https://doi.org/10.1126/science.aaa1934.

63. Darmanis, S., Sloan, S.A., Zhang, Y., Enge, S., Caneda, C., Shuer, L., Gephart, M.G., and Barres, B.A. (2015). A survey of human brain transcriptome diversity at the single cell level. Proceedings of the National Academy of Sciences *112*, 7285–7290. https://doi.org/10.1073/pnas.1507125112.
